# Therapeutic Neurostimulation in Obsessive-Compulsive and Related Disorders: A Systematic Review

**DOI:** 10.3390/brainsci11070948

**Published:** 2021-07-19

**Authors:** Nicola Acevedo, Peter Bosanac, Toni Pikoos, Susan Rossell, David Castle

**Affiliations:** 1 Centre for Mental Health, Swinburne University of Technology, John Street, Melbourne, VIC 3122, Australia; tpikoos@swin.edu.au (T.P.); srossell@swin.edu.au (S.R.); 2 St. Vincent’s Hospital Melbourne, 41 Victoria Parade, Melbourne, VIC 3065, Australia; peter.bosanac@svha.org.au (P.B.); david.castle@camh.ca (D.C.); 3 Department of Psychiatry, University of Melbourne, Melbourne, VIC 3010, Australia; 4 Centre for Addiction and Mental Health, 252 College Street, Toronto, ON M5T 1R7, Canada

**Keywords:** neurostimulation, neuromodulation, obsessive-compulsive disorder (OCD), neurocircuitry, electroconvulsive therapy (ECT), transcranial direct current stimulation (tDCS), transcranial magnetic stimulation (TMS), deep brain stimulation (DBS)

## Abstract

Invasive and noninvasive neurostimulation therapies for obsessive-compulsive and related disorders (OCRD) were systematically reviewed with the aim of assessing clinical characteristics, methodologies, neuroanatomical substrates, and varied stimulation parameters. Previous reviews have focused on a narrow scope, statistical rather than clinical significance, grouped together heterogenous protocols, and proposed inconclusive outcomes and directions. Herein, a comprehensive and transdiagnostic evaluation of all clinically relevant determinants is presented with translational clinical recommendations and novel response rates. Electroconvulsive therapy (ECT) studies were limited in number and quality but demonstrated greater efficacy than previously identified. Targeting the pre-SMA/SMA is recommended for transcranial direct current stimulation (tDCS) and transcranial magnetic stimulation (TMS). TMS yielded superior outcomes, although polarity findings were conflicting, and refinement of frontal/cognitive control protocols may optimize outcomes. For both techniques, standardization of polarity, more treatment sessions (20), and targeting multiple structures are encouraged. A deep brain stimulation (DBS) ‘sweet spot’ of the striatum for OCD was proposed, and CBT is strongly encouraged. Tourette’s patients showed less variance and reliance on treatment optimization. Several DBS targets achieved consistent, rapid, and sustained clinical response. Analysis of fiber connectivity, as opposed to precise neural regions, should be implemented for target selection. Standardization of protocols is necessary to achieve translational outcomes.

## 1. Introduction

Obsessive-compulsive disorder (OCD) is characterized by distressing thoughts (obsessions) and repetitive mental or behavioral acts (compulsions). The most recent edition of the Diagnostic and Statistical Manual (DSM) for Mental Disorders [1] broadened OCD and related disorders to encompass: body dysmorphic disorder (BDD), i.e., a preoccupation with perceived flaws of defects in physical appearance or body build with repetitive behaviors or mental acts in response to these; hoarding disorder (HD), i.e., persistent difficulty and distress associated with discarding possessions, with excessive accumulation and/or acquisition of the latter; trichotillomania, i.e., recurrent, obsessive hair-pulling; and excoriation disorder, i.e., recurrent skin-picking with resultant skin lesions. Whilst Tourette Syndrome (TS) is classified as a tic rather than an OCRD in the DSM-5, significant obsessive-compulsive symptomatology is present in almost half of affected people [2]. Moreover, there is considerable genetic contribution in obsessive-compulsive symptomatology, Tourette’s Syndrome, and hoarding, with covariation of phenotypes ranging from 50.4% to 61.1% [3]. The syndromal aggregation of disorders in the DSM-5 is based on a natural history of symptom similarities outweighing differences.

There is a substantial need for novel effective treatment interventions for OCD and related disorders. Even when the best available treatments are applied, approximately 30–40% of people experiencing OCD do not respond adequately [4], and 10% remain severely afflicted [5,6]. Treatment-refractoriness in these disorders is variably defined, but usually centers on suboptimal response to adequate therapeutic trials of serotonergic reuptake inhibitor antidepressants (selective serotonin reuptake inhibitors, (SSRIs) or clomipramine), as well as evidence-based psychological treatments such as cognitive-behavioral therapy (CBT) undertaken by experienced clinicians.

### 1.1. Psychopathology

Despite inconsistencies in structural and functional neuroimaging findings, hyperactivity of cortico-striato-thalamo-cortical (CSTC) circuits involving a central role of the basal ganglia is the prevailing neurobiological model of OCD pathogenesis [7,8,9]. Further explication of this model includes the orbitofrontal cortex (OFC), that integrates limbic and emotional information into behavioral responses, and the anterior cingulate cortex (ACC), that is integrally involved in motivated behaviors. Prefrontal goal-orientated behaviors and reward processing are thought to be modulated by the Nucleus Accumbens (NAc), which is at the motor-limbic interface of cortico-subcortical circuits. The NAc may also integrate contextual information from the hippocampus and emotional information from the amygdala. In OCD, there is hyperactivity of the caudate nuclei and OFC, as evidenced by increased metabolism in PET scanning [10]. Impairments in the neuropsychological function of executive control, memory, visuospatial abilities, attention, and processing speed are prominent in OCD and related to frontostriatal dysfunction [11]. Despite avoidance of anxiety provoking stimuli and engagement in compulsive rituals, classical conditioning mechanisms can maintain or exacerbate obsessive-compulsive symptomology [12]. Thus, OCD has a complex clinical profile involving bio-psycho-social determinants. Moreover, the central role of the prefrontal cortex in OCD has led to this region being identified both as a functional neuroimaging marker of the disorder and a therapeutic target [13].

Whilst altered functioning of the CSTC circuits and the ACC have been consistently highlighted in OCD, frontostriatal hyperactivity may also be associated with obsessive thoughts and compulsive behaviors in BDD [14]. The ACC is also involved in the neurocircuitry of BDD, along with right OFC, thalami, hippocampi, and amygdalae [15]. Unlike OCD, occipital hypofunction may contribute to core psychopathology in BDD [14]. There is also preliminary evidence of reduced local amygdala connectivity in BDD [16]. TS also involves altered frontostriatal circuitry [17,18], but predominantly involving the motor and premotor cortices and dorsal striatum [17], potentially explaining why motor tics are more common in TS than the other OCRD. Similarly, trichotillomania and skin picking disorder involve altered frontal-striatal circuits and decreased prefrontal control, as well as motor and reward regions [19].

### 1.2. Neurostimulation

Given that a significant proportion of people with OCRD are refractory to extant evidence-based treatments, the etiology and neurocircuitry of OCD and related disorders support consideration of neurostimulation as a means of targeting particular areas of the brain via activation or inhibition, and, in turn, modulation of neurocircuitry and neuroplasticity farther afield [20]. Furthermore, neurostimulation has the potential to personalize treatments based on patient-specific neural substrates and the complex interactions among different neural circuits.

**Figure 1:**
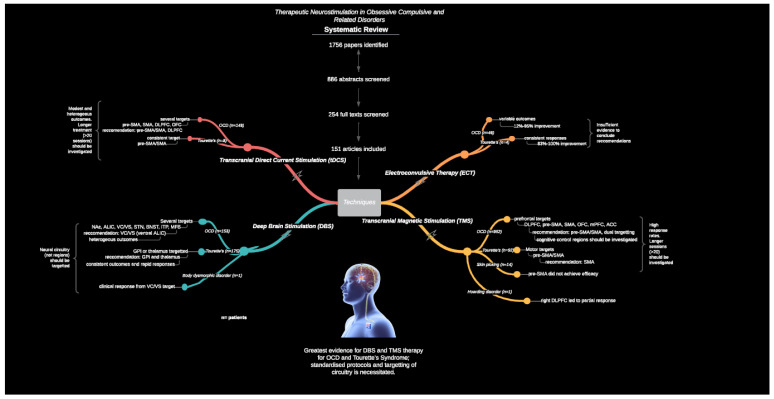
Graphical abstract.

In light of the evolving knowledge of neurocircuitry in OCRD and the ongoing treatment gap for these disorders, we systematically reviewed neurostimulation modalities in these disorders, and herein address their current and potential role in the continuum of care. We specifically included OCD, BDD, trichotillomania, excoriation disorder, HD, and TS in order to test for between-disorder similarities and differences. Reviewed data were collated with the aims of (1) reviewing the clinical significance of neurostimulation therapies for OCD and OCRD; (2) making clinical recommendations for optimal stimulation protocols; (3) reporting on limitations and strengths of current neurostimulation protocols in order to (4) identify future directions for greater consistency and validity of treatment effects. Figure 1 shows a graphical abstract in the form of a flow diagram of the techniques, conditions, and neurostimulation targets identified. A brief summary for each technique, and all investigations is provided. 

## 2. Methods

### 2.1. Protocol

The Preferred Reporting Items for Systematic Reviews and Meta-analyses (PRISMA) statement was followed for the review process [21]. The protocol was registered with the international prospective register of systematic reviews (PROSPERO) registration number: CRD42020171054.

### 2.2. Search

The databases PubMED and CINAHL were searched with the following terms: (obsessive compulsive disorder OR body dysmorph* OR hoarding disorder OR skin picking disorder OR excoriation disorder OR trichotillomania OR hair-pulling OR Tourette*) AND (neurostimulation OR deep brain stimulation (DBS) OR electroconvulsive therapy (ECT) OR transcranial direct current stimulation tDCS OR vagus nerve stimulation (VNS) OR transcranial magnetic stimulation (TMS)). The search was conducted with no specified start date up until 1 January 2020. Citations of identified articles and recent systematic reviews [22,23,24,25,26,27,28,29,30,31,32] were checked for additional articles.

### 2.3. Inclusion and Exclusion Criteria

Inclusion criteria:

(1) Investigation of any neurostimulation intervention (DBS, ECT, TMS, tDCS) in patients with a primary diagnosis of OCD or OCRD (BDD, trichotillomania, excoriation disorder, HD) or TS, with no adjunct treatment except pharmacology.
(2) Assessment at pre- and post- treatment using a standardized outcome.
(3) English language literature.
(4) Peer-reviewed article with primary data including randomized control trials (RCTs), open-label (OL) trials, multisite studies, case studies and letters to the editor.

If stimulation parameters, diagnostic method, or response criteria were not stated, or a retrospective design was implemented, this was not an exclusion but a limitation, and was considered in the quality assessment.

Exclusion criteria:

(1) Investigation of patients that did not have a primary diagnosis of OCD or OCRD, or the primary diagnosis was unclear.
(2) Comorbid severe psychiatric condition including schizophrenia, catatonia, bipolar or psychosis. Common comorbidities that did not warrant exclusion included major depressive disorder (MDD), anxiety, attention-deficit- hyperactivity- disorder (ADHD), and personality disorder.
(3) Investigation of adjunct behavioral therapy or additional neurostimulation intervention.

As per the PRISMA method, a total of 1756 records were identified, and an additional 68 were identified through reference lists and previous review articles. After duplicates were removed, 886 records were screened by title and abstract by one author (T.P. or N.A.); 254 were deemed eligible. These 254 full text articles were reviewed for eligibility by two authors (T.P. or N.A.), and 153 were included in the final qualitative synthesis, at which stage 101 were excluded (see Supplementary File S1, S1 PRISMA diagram). Any discrepancies were resolved by a third author (S.R.). Across each neurostimulation technique, insufficient or no protocols were comparable, and thus, quantitative synthesis was not appropriate.

### 2.4. Data Extraction

The following data were extracted from the selected articles: clinical characteristics, demographics, methodology, stimulation protocols, outcome measures, clinical and statistical significance, and adverse events. We also provided commentary on the limitations and strengths of each study.

### 2.5. Risk of Bias Assessment

Items from the Cochrane Collaboration Risk of Bias (RoB) tool [33] that were relevant were used to evaluate risk of bias, and are presented in Supplementary File S2, S2 Risk of Bias Table.

### 2.6. Quality Assessment

No quality assessment tool previously implemented in a neurostimulation review that was appropriate for the current protocol was identified. The Oxford Quality Scoring System [34] and the Cochrane Grading of Recommendations Assessment, Development and Evaluation (GRADE) tool [35] were thus adapted to develop a tool in line with the current protocol, variables, and outcomes, and are presented in Supplementary File S3, S3 Quality assessment table.

### 2.7. Reporting of Data

The primary clinical outcomes were reported and discussed for each neurostimulation technique. The primary outcome for OCD articles was the Yale Brown Obsessive Compulsive Scale (YBOCS), which is a clinician rated scale that provides a symptom severity score for obsessions (0–20), compulsions (0–20), and an overall score (0–40). The primary outcome for TS was the Yale Global Tic Severity Scale (YGTSS), which is a semi-structured interview that assesses motor tics, phonic tics, total impairment, and total severity. The YGTSS provides a tic severity score (motor tic severity + phonic tic severity, 0–50), and/or a global severity score (tic severity+ tic impairment, 0–100). The primary outcome for BDD was the Body Dysmorphic Disorder- YBOCS (BDD-YBOCS), adapted from the YBOCS scale to assess preoccupation, insight, and avoidance, as well as obsessions and compulsions; it generates an overall score (0–48). The primary outcome for skin picking was the Neurotic Excoriation- YBOCS (NE-YBOCS), which provides a score for thoughts and behavior related to tics (0–20) and a total score (0–40). The primary outcome for hoarding disorder was the Saving Inventory- Revised (SI-R), which assess difficulty discarding, clutter and acquisition; it generates sub-scores and an overall score (0–92). All scales assess symptoms in the previous 7 days, or 7–10 days in the case of the YGTSS.

Findings were considered in the context of clinical rather than statistical significance, as the two often do not correspond and the former is more sensitive and ecologically valid. Pallanti defines full treatment response as ≥35% reduction in YBOCS, and a partial treatment response as ≥25% reduction in YBOCS [36]. If a different treatment response criterion was reported within articles, then this was used to report responders; if not, the widely accepted criterion was used [36]. In the case of DBS, a full response is most often categorized as ≥40% reduction in YBOCS, which was implemented in the absence of a defined treatment response.

Results were categorized for each technique, then condition and target, followed by a discussion, before reporting results on the consecutive technique. Prior to reporting the results of each technique, a synopsis on the proposed mechanisms of action, important considerations for stimulation protocols, and methodological limitations were described. This structure was implemented in order to present the data coherently, and for ease of interpretation, i.e., to present important theoretical and technical insights; to discuss parameters of stimulation protocols; to allow for transdiagnostic interpretation; and to account for disparities and limitations in methodology which were specific to each technique. There were a range of targeted regions in the TMS and DBS studies; thus, the results were discussed in the context of each target as well as common themes identified for these techniques. Lastly, a general discussion provides clinical recommendations across all techniques and conditions within the context of CSTC circuitry. Due to the breadth of conditions and techniques, an in-depth analysis of secondary clinical outcomes was beyond the scope of the review. Rather, notable findings of depression and anxiety in relation to neurostimulation-mediated change in obsessive-compulsive (OC) symptoms were reported.

## 3. Results and Discussion

A brief report of cohort studies is provided herein. Refer to the supplementary results for an in-depth report of all protocols for tDCS, TMS and DBS, including methodology aspects (i.e., blinding, placebo effects, treatment dose and titration), stimulation parameters, anatomy, and individual response patterns, which are integral to the discussion.

### 3.1. ECT Results

ECT involves the application of an electric charge via electrodes placed on the scalp to induce transient generalized seizures for therapeutic benefit [37]. Beyond the induction of generalized seizures, ECT does not target specific areas of the brain.

Thirty-six articles were screened for eligibility, and 10 were included in the final synthesis: 6 for OCD and 4 for TS. Of the 26 articles excluded, 15 lacked a standardized outcome measure of primary symptoms; in eight, the primary diagnosis was not an OCRD or this was unclear; in a further two, both limitations were present; finally, in one investigation two neurostimulation therapies were applied. Of the included articles, OCD articles consisted of one retrospective review of medical records, four case series, and one case report, whilst TS articles consisted of four case reports. No RCT or prospective investigation of a cohort with greater than five patients was identified (across included and excluded articles), and most articles reported on a single patient. Further, clinicaltrials.gov was searched for potential RCTs that had not yet been published or identified, but no results were obtained. The final sample therefore included 46 OCD and four TS patients. Table 1 shows summary results of ECT investigations for OCD and Table 2 shows summary results of ECT investigations for TS.

#### 3.1.1. ECT Results for OCD

The only cohort study of ECT was a retrospective review reporting a mean symptom improvement of 42% following treatment, and 35% improvement 12 months later [38]. Pooled together, the case studies for OCD revealed a response rate of 79% (11/14); within those who responded, symptom improvement of 43–95% was achieved [39,40,41,42,43].

Quantitative follow-up outcomes were reported in 43% of case studies (6/14 patients). In one study [41], three out of five patients were responders following treatment; only one remained a responder at 3- and 6-month follow-up, according to the clinical global impression (CGI) scale (not YBOCS). Another patient relapsed to baseline twice following consecutive cycles of ECT [42].

Depression symptoms showed clinically significant improvements of 48–62% in two investigations [38,40], and were not reported in the other articles.

#### 3.1.2. ECT Results for TS

Improvement rates between 83–100% were reported; thus, all four patients achieved clinical response [44,45,46,47]. Qualitative follow-up outcomes were reported in three cases: all experienced complete remission with no relapse up to 8 months following treatment [44,45,46], yet no studies reported quantitative (i.e., YGTSS) outcomes at follow-up. Depression outcomes were not reported, despite two patients reportedly experiencing comorbid depression.

### 3.2. ECT Discussion

We identified variable response rates to ECT in OCD patients, with a lack of quantitative evidence of long-term efficacy. In contrast, TS patients consistently showed substantial response and remission rates, even though only four patients were reported on. The risk of bias was low for all articles (S2); the quality assessment rated no studies as good, seven as moderate, and three as poor (S3).

Quality ratings were impacted by a lack of RCTs and cohort studies, poor reporting of clinical demographics and stimulation parameters, and a lack of quantitative follow-up. Transient effects of general fatigue and short-term memory loss were reported for some patients.

OCD patients had heterogeneous symptom profiles, including obsessions of sexuality, persecution, checking, cleaning, slowness, contamination, and pathological doubt. Illness duration varied from abrupt onset of 2.5 months to 13 years, and treated individuals were between 18–47 years of age, (demographics missing for some patients). TS patients had similar complex motor and vocal tics, coprolalia, and self-injurious behaviors among other impairments; illness duration was 8–30 years, and age at ECT was between 18–36 years.

#### 3.2.1. Pattern of Response

Within OCD studies, 79% of case reports were responders, with change between 43–95%; and within a single cohort, 42% change was achieved. Previous reviews have reported ‘positive response’ rates of 60% [26] and 73% [24] for ECT in OCD but acknowledged that only 17% of studies employed standardized assessments. There was a lack of evidence beyond subjective accounts of long-term efficacy. It was stated that patients remained ‘symptom free’ or ‘maintained response’ up to 4 years following treatment, in the absence of quantitative evidence. In line with the current findings, previous reviews also demonstrated a lack of standardized follow-up outcomes, which were often not reported (50–66%). When follow-up was reported, relapse or deterioration occurred in 35–55% of cases [24,26]. This highlights the vagueness and subjectivity of clinical findings, as a ‘positive response’ could indicate that the patient subjectively reported they felt better, or it could mean the patient had a 95% improvement assessed by a clinical tool; these disparate scenarios cannot be grouped and interpreted as proportional.

The only cohort study [38] reported that improvement was maintained in 42% of patients’ post-treatment to 35% at 12-month follow-up; only 16% (5/32) received maintenance ECT due to deterioration, which indicates a greater level of sustained efficacy than reported in case reports. Within the cohort study, all patients resumed CBT, which may have maintained the therapeutic effect of ECT. The primary outcome was the Maudsley Obsessional Compulsive Scale (MOCI), as opposed to the YBOCS. Nevertheless, the cohort was highly treatment resistant and chronic, with an average disease history of 27 years, and seven medication trials. Thus, with adequate clinical follow up and adjunct treatment, highly resistant OCD patients may achieve long-term symptom improvement from ECT.

TS patients responded consistently to ECT (100% responded and 75% reached remission), and there was no reported deterioration following treatment cessation. However, this conclusion is in the absence of follow-up standardized assessments and is based on only four patients.

There were no predictors of treatment response due to inconsistencies in OCD patients and a ceiling effect of clinical efficacy in TS patients. Although ECT had a differential effect on depression based on response, comorbid depression was not correlated with YBOCS response [38].

#### 3.2.2. Treatment Dose

A treatment-efficacy dose relationship could not be estimated due to the very small sample size, as well as the fact that some studies did not specify doses. The number of sessions in the OCD and TS cohort varied from 3–14 and 6–37 sessions, respectively. Treatment duration was not always specified for OCD patients, and was estimated at a maximum of 5 weeks, whereas TS patients underwent treatment for 5 weeks to 5 years. In the cohort study, the range of sessions administered was not described, only the average of 3.5 treatments. In the TS studies, 50% included maintenance ECT, and in one study, this was extended for 5 years. The patient with comorbid TS and OCD completed only six sessions and experienced complete remission of OCD and almost complete remission of TS.

OCD patients had a more variable clinical response to ECT, but responders required fewer treatments. Whilst TS patients consistently responded well to ECT, they required longer treatment regimes. Although there was no relapse reported in the TS studies, it is unclear whether maintenance ECT was administered due to clinical deterioration or was predefined.

### 3.3. tDCS Results

tDCS generates a week, direct current to the scalp between two electrodes and induces polarity dependent changes in the resting membrane potential at a subthreshold level [48]. The polarity effects of tDCS on neural elements are postulated to have an anodal-excitation and cathodal-inhibition (AeCi) relationship. Theories on the mechanisms of action are based on investigations at low doses (~10 min, 1 milliampere, mA) over the motor cortex in healthy individuals. Polarity effects are likely to differ with consecutive applications of relatively high doses (1 mA, 10 min) on brain regions involved in cognitive and emotional processes, in which transmitter availability and brain function are altered in psychiatric disorders.

Theoretically, tDCS is capable of inducing lasting behavioral changes mediated through synaptic plasticity mechanisms of long-term potentiation (LTP) and long-term depression (LTD); this notion is supported by behavioral, neurochemical [49], and neuroimaging evidence [50]. Neuromodulation is not restricted to the region under the electrodes, and widespread cortical and subcortical modulation can occur [50]. Modulation of discrete cortical excitation and diffuse network connectivity is of particular importance in targeting OCRDs, which are underpinned by hyperactive nodes and network imbalances. tDCS therapy presumably restores pathological excitatory/inhibitory imbalances.

Twenty-six articles were screened for eligibility, with 19 being included in the final synthesis: 15 for OCD and 4 for TS treatment. Four articles were excluded due to a lack of standardized assessment of primary symptoms, two because the primary diagnosis was not an OCRD or was unclear, and one because immediate post-treatment outcomes were not reported. Of the included articles, OCD investigations consisted of five case reports, five open-label trials, three double blind RCTs (two sham controlled), two case series, and one double blind case report. TS investigations consisted of two case series/pilot data of a double blind RCT, one case series, and one case report. The final sample included 148 OCD patients and 8 TS patients. Table 3 and Table 4 show summary results of tDCS investigations for OCD, and Table 5 shows summary results of tDCS investigations for TS.

#### 3.3.1. tDCS Results for OCD

Pre-SMA/SMA investigations included two RCTs (one without a sham condition), one open-label trial, and four case studies. Across cohorts, anodal tDCS led to 7–22% improvement and cathodal tDCS led to 15–20% improvement [53].

DLPFC investigations included an open-label trial and two case studies. Improvement of 65% occurred on a cohort level from cathodal tDCS of bilateral DLPFC [55]. Dual targeting of the right OFC (cathodal) and left DLPFC (anodal) led to 23% improvement [52].

OFC investigations included one RCT, two open-label trials and two case studies. Open- and closed-label cathodal tDCS of the left OFC led to 5% and 20% improvement, respectively [51].

Open-label transcranial alternating current stimulation (tACS) over the bilateral frontotemporal region led to 52% improvement, 86% full response, and 100% partial response [54].

#### 3.3.2. tDCS Results for TS

TS investigations included four case reports of cathodal tDCS over motor regions. tDCS of pre-SMA or SMA led to improvements between 20–43% [67]. Stimulation of both the pre-SMA and SMA led to incongruent outcomes, with improvement in the YGTSS but dramatic increase in tic counts [68]. tDCS of the primary motor cortex led to 11–30% improvement [66].

### 3.4. tDCS Discussion

The risk of bias was low for 15 articles and medium for four (S2); the quality assessments rated six as good, 13 as moderate, and one as poor (S3). Quality assessments highlighted a lack of RCTs with a sham control (only two studies met criteria), a failure to report the number of clinically significant responders (four met criteria), and a lack of follow-up outcomes (10 met criteria). Statistical significance was often the focus of the results, which has limited clinical relevance; for example, mean change as little as 4.7% in 20 patients was sufficient to yield statistical significance [56], yet arguably, this does not reflect clinical significance.

OCD patients had failed between one and five medication trials, and case studies exemplified higher treatment resistance in relation to cohort studies. Several comorbid mood disorders were reported and did not appear to interfere with efficacy. Illness duration in the case studies ranged between 1–23 years of illness, and type of obsessions varied (predominantly of contamination, symmetry, aggression, sexuality, and guilt). TS patients had disparate baseline severity, and illness duration ranged between 4–45 years. Information on symptom domains and the definition of treatment resistance was lacking.

When reported, all except for six OCD and two TS patients were taking prescribed medication; this did not appear to impact treatment response, although SSRIs can enhance and prolong the facilitation effect of anodal tDCS [69]. Depression symptoms improved by 10–87% and anxiety symptoms by 17–100% in OCD patients and were not reported in TS patients.

OCD protocols targeted the pre-SMA, SMA, DLPFC and OFC; yet the return electrode placement—which determines the distribution of current flow and is a critical determinant of network effects [70]—was variable. Stimulation involved 2 mA for 20 min, except for two studies in which it was 30 min [55,71], across 8–20 sessions with varying polarities and hemispheres. Protocols for TS articles targeted different motor regions including the pre-SMA, SMA and motor cortex. The return location and polarity was consistent. Stimulation was applied between 1.4–2 mA for 20 or 30 min across 10–18 sessions.

#### 3.4.1. OCD, Pre-SMA and SMA Targets

Within-group and within-patient analysis showed a superiority of cathodal stimulation [53,63], yet randomized controlled and open-label studies revealed only modest effects of cathodal tDCS with 17–29% symptom improvement [53,57,58,63]. Anodal stimulation was directly investigated only in case reports: three patients responded with symptom change of 40–70% [61,64]. Benefit from anodal tDCS over the pre-SMA/SMA occurred after twice daily sessions rather than daily sessions. Polarity comparison studies showed superiority of daily cathodal sessions [53,63]. Thus, a perceived superiority of cathodal stimulation may have biased previous reviews to conclude ineffectiveness of anodal stimulation [72].

Cathodal and anodal polarity after-effects are linear for intensities up to 1 mA and durations up to 13 min; however, opposing polarity effects may not be apparent beyond these parameters. Cathodal and anodal stimulation at 2 mA can enhance cortical excitability for 120 and 60 min, respectively, returning to baseline after 6–8 h [73]. The timing between consecutive tDCS sessions is also an important determinant of the cumulative polarity effect. A second application of tDCS during the after-effects of the first can enhance and prolong the polarity effects on cortical excitability, whereas a second application outside the after-effects of the first can attenuate or abolish the polarity effects [74,75]. In turn, applications within this review of 2 mA cathodal tDCS may lead to cortical excitability that is enhanced by a second daily session. Cathodal SMA stimulation may be theoretically optimal [76], although twice daily anodal stimulation may cause greater inhibition and appears to be therapeutically optimal.

Follow-up data was lacking in many studies. In those four patients for whom follow-up was performed, one responder (anodal stimulation) further improved at one week follow-up [61], whilst one partial responder (cathodal stimulation) further improved at three-month follow-up [53]. Two patients who had not responded to cathodal stimulation did show improvement six months later [65].

The pre-SMA/SMA is involved in mediating important cognitive functions, particularly response inhibition, monitoring of conflicting information, and the control of internally triggered movements [77]. SMA dysfunction in OCD leads to reduced inhibition of the striatum, resulting in striatal hyperactivity, manifesting as inhibitory dysfunction related to intrusive ideas and ritualistic behaviors [78]. Modelling of a montage over the pre-SMA is predicted to modulate the medial PFC to the striatum [76], inclusive of the critical cortical and subcortical regions implicated in OCD pathophysiology. Thus, establishing the optimal stimulation montage for the pre-SMA/SMA can potentially alleviate hyperactivity in several regions and suppress intrusive thoughts and behaviors involved in OCD.

#### 3.4.2. OCD, DLPFC Target

Investigations of DLPFC implemented disparate montages, studies failed to report clinically significant responders, and there were no randomized or controlled protocols. Thus, conclusions that can be drawn are limited. The open-label cohort study implemented an extensive stimulation montage (bilateral DLPFC cathode contacts and three anode/return contacts), with 65% symptom improvement, rising to 81.5% at three-month follow-up [55]. Case reports did not demonstrate efficacy to anodal stimulation of the left and cathodal stimulation of the right DLPFC [71], or cathodal stimulation of the left DLPFC [59].

There was insufficient evidence to establish the treatment parameters (i.e., intensity, duration, number of sessions or electrodes) that are the most important determinants of clinical outcomes. Across investigations of the DLPFC, 10 sessions were insufficient to induce symptom change [59], whilst 15 or 20 sessions appeared to be sufficient to reach a partial response but not to maintain efficacy [52,71]; however, 15 sessions with increased stimulation electrodes, and thus area of current, were shown to be highly effective [55]. Thus, it appears that at least 15 sessions of cathodal DLPFC stimulation should be applied to achieve a clinical response, and greater stimulation intensity and current distribution may be required for response to be optimized and maintained.

#### 3.4.3. OCD, OFC Target

Post-treatment efficacy was comparable between open- and closed-label conditions [51,56], in which 22% symptom improvement occurred, and 33% response achieved. However, the open-label trial provided valuable insight that efficacy was greatest at three-month follow-up. Cathodal tDCS of the left OFC and anodal tDCS of the right OFC showed a delayed partial response in one patient [60] and a dramatic clinical response in another [62]. Two patterns emerged: (1) greater response to cathodal tDCS of the left OFC was associated with less treatment resistance [56]; and (2) all investigations showed further improvement at follow-up, identifying a delayed and prolonged pattern of response.

#### 3.4.4. Transcranial Alternating Current

Klimke (2016) demonstrated a dramatic clinical response in six out of seven OCD patients using bilateral fronto-temporal tACS [54]. Further, the results were achieved with a fraction of the stimulation intensity typically employed in tDCS protocols (0.65 mA vs. 2 mA). Rather than the direct current used in tDCS, tACS produces sine-wave stimulation at a frequency of interest. It is theorized that tACS leads to manipulation and entrainment of intrinsic cortical oscillations in a frequency-specific manner [79]. It is known that phase and frequency are fundamental parameters of neuronal function, and thus LTP; also, endogenous oscillations can entrain to extrinsic rhythmic stimuli [80]. Thus, tACS has relevance for implicit learning, and follow-up data would be valuable to establish a possible learning effect. Applications of tACS are limited and novel, and sham controlled investigations of the current montage are warranted.

#### 3.4.5. TS, Motor Targets

Four case studies of cathodal tDCS of motor regions yielded mixed outcomes. The pre-SMA and SMA showed superiority, but even so, only two of six patients achieved a response, with overall improvements ranging between 6–41%. Follow-up data were available for three patients and showed that response was maintained for up to six months. Cathodal tDCS of the motor cortex in two patients did not lead to clinical response; however, the number of sessions was likely too low (five) to lead to behavioral change. The case studies that achieved higher clinical efficacy [25,67] implemented daily cathodal tDCS sessions of the pre-SMA/SMA at the relatively low stimulation dosage of 1.4 mA.

SMA activity is positively correlated with tic symptom severity in TS, and thus, is implicated as a neural substrate [18]; yet distinction between an initiation and suppressive role has not been established. It is concluded that a low dose (~1.4 mA) of daily cathodal tDCS over the pre-SMA and SMA leads to clinical response and maintained efficacy in case studies in TS; however, randomized controlled evidence is required.

### 3.5. TMS Results

TMS produces a rapidly changing magnetic field perpendicular to the plane of the coil that penetrates the skull and induces an electric field perpendicular to the magnetic field [81]. In turn, axons within underlying cortical and subcortical regions depolarize and activate neural circuits whilst interacting with spontaneous oscillatory rhythms [82]. Low frequency (LF, 1 Hz) and high frequency (HF, ≥5 Hz) rTMS respectively cause inhibition and facilitation of axons within the motor cortex and are assumed to have the same effect on nonmotor functions [83]. The sustained effects likely depend on NMDA and GABA receptors, with LTP and LTD, although the precise mechanisms are unknown [84]. The effects of rTMS depend inter alia on the orientation of the coil, the stimulation protocol (frequency, intensity, and pulse parameters), the resting brain state, and the axonal threshold of activation. Modulation may involve local and distant, excitatory and/or inhibitory axons. Low stimulation intensities will activate a limited number of neurons, activation is more likely if neurons are close to the firing threshold, and currents parallel to the axon are more likely to reach activation [85].

Novel devices and stimulation protocols have been implemented into clinical research to achieve focal targeting. Neuronavigation implements imaging to localize a within-subject target in order to overcome differences in anatomy. Figure-eight coils concentrate current by two-fold in the mid-point of the two loops, compared to circular coils. Deep TMS coils (double cone or H coils) allow a slow fall-off of the intensity of the magnetic field, thus achieving greater strength and depth of penetration [81]. Alpha frequency rTMS is based on the notion that various mental disorders express abnormal EEG power band frequencies, and alpha (α) has shown to be abnormal in prefrontal and temporal lobes in OCD [86]. Lastly, theta burst stimulation (TBS) is typically applied for shorter periods (1–3 min) at lower intensities and can induce rapid and long lasting inhibitory (continuous TBS) or excitatory (intermittent TBS) cortical changes [81].

The neural effects of rTMS are accompanied by sensory effects, predominantly a loud clicking noise and skin sensations. This poses difficulties with blinding. Successful blinding does not guarantee placebo effects are controlled for; the sham condition should fulfil certain technical aspects to minimize disparities across conditions. An ideal placebo condition should (1) not result in cortical stimulation, (2) produce sensory sensations identical to those of real stimulation, and (3) be positioned the same way as the active coil [87]. There is a trade-off between the absence of neural effects and presence of somatic effects. In the articles reviewed here, sham methods were assessed through the RoB assessment (S2).

Sixty-seven articles were screened for eligibility, with 48 being included in the final synthesis. Of these, 36 were for OCD, seven for TS, three for comorbid TS and OCD, one for skin picking, and one for hoarding disorder. Eighteen articles were excluded due to a lack of standardized assessment of primary symptoms (*n* = 6), adjunct behavioral therapy (*n* = 5), lack of original data (*n* = 3), the primary diagnosis not being an OCRD or being unclear (*n* = 2), methodological concerns with the stimulation protocol (*n* = 1), and outcomes not being reported directly from the patient (*n* = 1). Of the excluded articles, there were six RCTs, four open-label trials, four case reports, two retrospective studies, one case series, and one review article. Of the included articles, OCD investigations consisted of 21 RCTs (18 double blinded single-site, two multisite double blinded, one single blinded), four open-label trials, five case reports, two investigations as standard clinical care, one single blind nonrandomized trial, two retrospective reports, and one case series. TS investigations consisted of three RCTs (one each multisite double blinded, single-site double blinded, and single blinded), one pilot RCT, three open-label trials, and one case series. In addition, there was one open-label trial, one case report, and one case series for patients with comorbid OCD and TS. The skin picking investigation was a double-blinded RCT, and the hoarding disorder investigation was a case report. The final sample included 952 OCD patients, 92 TS patients, 14 skin picking patients, three patients with comorbid OCD and TS, and one hoarding disorder patient. Table 6 and Table 7 show summary results of rTMS investigations for OCD, Table 8 and Table 9 show summary results of rTMS investigations for TS. Table 10 and Table 11 show summary results of rTMS investigations for skin picking disorder and HD.

#### 3.5.1. TMS Results for OCD

Investigations of the DLPFC encompassed nine RCTs, one single blinded nonrandomized controlled trial, one open-label trial, and two case studies. High frequency and alpha guided rTMS of bilateral DLPFC led to 26–37% improvement [102,104]. rTMS of left DLPFC led to 8–30% improvement [89,91,92,95]; and rTMS of the right DLPFC led to 9–45% improvement [88,89,93,110], respectively. Also, continuous (inhibitory) TBS of the right DLPFC led to 58% improvement [121].

Stimulation of the pre-SMA and SMA was investigated in four RCTs (one multisite), one randomized open-label trial, three open-label trials, and four case studies; all employed LF rTMS. Closed- and open-label investigation achieved symptom improvement between 23–49%, and 1 trial led to 8% change [96,107,109,113] increasing the treatment sessions by two-fold changed improvements from 25% to 49% [96]. TMS targeting of the SMA for OCD symptoms and right DLPFC for comorbid MDD led to a high response rate of 83% and 42% improvement [111]. TBS treatment yielded 13% improvement [117].

Investigations of the OFC included two RCTs and a retrospective study, all using LF rTMS. Symptom improvements of 19%–24% were achieved and maintained at 4–10 weeks [94,115], but not within a trial using deep TMS [101]. When patients were prescribed LF rTMS of the SMA or OFC based on their preference, 27% improvement was reached overall [118].

LF and HF rTMS of other prefrontal regions were investigated in two RCTs (one multisite), two open-label trials, and one case study. Deep TMS of the medial PFC and ACC led to 44% and 45% improvement across a pilot and multisite study [114,116]. LF and HF rTMS of the medial PFC led to comparable outcomes of 39% and 40% improvement, respectively [105,106].

#### 3.5.2. TMS Results for TS

Investigations for pre-SMA and SMA included two RCTs, three open-label trials and three case studies, all of which implemented LF rTMS. Closed-label conditions led to response in 33% and 50% when implementing continuous TBS [128,129], while open-label trials led to 34%, 30% and 5% improvement [126,127,130]. Additional treatment led to greater efficacy, and when patients were followed up, outcomes were maintained at 3 and 6 months.

Two small RCTs targeted the premotor and motor cortex, which led to no change and 29% improvement, respectively [124,125].

#### 3.5.3. Other Conditions

A small trial of LF rTMS of the pre-SMA for skin picking led to a 36% improvement, followed by a 70–110% decline [133]. In one study, hoarding disorder patients achieved 30% improvements from LF rTMS of the right DLPFC [134].

### 3.6. TMS Discussion

For the TMS studies reported here, the risk of bias was low for 20, medium for 21, and high for seven studies (S2); the quality assessments classified 28 articles as good, 19 as moderate, and one as poor (S3). Within the quality assessment, the main methodological limitation was lack of follow-up (19 articles met the criteria), as well as a lack of controls and adequate blinding in a number of studies. The lack of follow-up outcomes is an important limitation, as rTMS effects are often slow and progressive [135], and the onset and duration of the effect is critical for insight into clinical care. Of the included articles, 43% (20/49) reported dropouts: out of the entire sample, 3.2% (31) of patients withdrew during treatment and 1.4% (14) did so prior to treatment. The only serious adverse event reported was the onset of manic symptoms in an OCD patient with comorbid bipolar disorder [98].

Treatment resistance was an inclusion criterion for all but one of the OCD trials [105], and only one TS investigation reported on treatment resistance criteria [130]. Within OCD, patient comorbidities included MDD (*n* = 136), eating disorders (*n* = 15), mood disorders (*n* = 9), panic disorder (*n* = 7), post-traumatic stress disorder (PTSD) (*n* = 4), personality disorders (*n* = 4), bipolar (*n* = 2), ADHD (*n* = 1), and generalized anxiety disorder (GAD) (*n* = 1). Within TS patients, comorbidities included ADHD (*n* = 36), OCD (*n* = 23) and MDD (*n* = 4). The presence or absence of comorbidities was not reported for 90 OCD and 25 TS patients. Patients were most often taking prescribed medication (714/873 OCD and 58/84 TS patients) but this was not reported on, or not controlled for in 94 OCD patients.

rTMS stimulation protocols contain numerous complex parameters, and no two studies presented comparable methodologies and stimulation protocols. When grouping each stimulation parameter within ranges (e.g., 10–20 sessions, 1200–1500 pulses etc.), there were no more than three studies with similar protocols for pre-SMA and DLPFC targets, which was insufficient for a meta-analysis. Stimulation protocols for OCD varied in the number of sessions (10–30 acute sessions, up to 44 for second phase protocols), pulses (750–3000, 600 for TBS), intensity (80–120%), frequency (1 Hz, 10 Hz, 20 Hz, 30 Hz, 50 Hz, α), coil type (figure-eight, deep TMS, one circular coil protocol), and targeting method (standardized coordinates, individualized neuronavigation). Protocols for TS also varied in session number (range 2–20), pulses (900–1800, 600 for TBS), intensity (80–110%), frequency (1 Hz, one 30 Hz protocol) and coil type (Figure-eight, one deep TMS protocol), and targeting method (standardized coordinates, one individualized neuronavigation protocol). The DLPFC, pre-SMA/SMA, OFC, mPFC and ACC were targeted for OCD; the pre-SMA/SMA and the motor cortex were targeted for TS. The pre-SMA was targeted for skin picking, and the right DLPFC for a HD patient.

Blinding is a perennial problem in TMS, and only six studies (13% of the total) assessed the effectiveness of blinding [92,93,99,114,116,129]. Three did not report the sham method [89,110,128]. It cannot be established whether protocols failed to assess the effectiveness of sham, or whether ineffective blinding was simply not reported. Studies implemented the following sham method:

1. A regular active TMS coil tilted 45–90 degrees from the scalp [88,91,93,94,95,97,103,104,107,118,133]
2. A sham coil, i.e., metal shield, de-activated coil, or purpose-built sham coil [59,96,98,99,100,101,109,113,114,116,117,125,129].
3. A sham coil placed on the same site as the active condition and an active coil placed 0.6- 1 m away from the scalp [92,102].

The tilt method produces the same sound and generally 25–50% of the electrical field compared to active TMS [136,137], thus likely producing some somatic sensations. The magnitude of electrical field that penetrates the scalp varies greatly as a function of the direction of the tilt and whether one or two wings are touching the scalp [136,137]; yet these aspects were not reported. A sham coil yields negligible stimulation to the cortex (3%), and produces the same sound as active TMS, yet lacks somatic sensations. The mechanisms of method 3 (active and sham coil) are not discussed in the literature, and thus, the limitations of this method are unknown. A sham coil combined with surface electrodes that induces somatic sensations is considered the sham gold standard [138], but none of the studies reviewed here implemented this method [87].

#### 3.6.1. OCD, DLPFC

##### Bilateral DLPFC

Within-group examination of laterality revealed a slight clinical superiority of HF rTMS of the right DLPFC immediately post-treatment that become a clear superiority at one-month follow-up [89]. Alpha (α) guided rTMS of bilateral DLPFC led to response in 36% of patients’ post-treatment, with minimal placebo effect, yet response was not maintained in three patients one week later [102].

##### Left DLPFC

Similar clinical response was achieved with LF (24%) and HF (30%) rTMS; however, comparable placebo effects occurred [91,92]. One study reported post-treatment effects and showed further improvement at two weeks, yet sham benefit was greater than active [91]. A response rate of 55% was achieved when HF rTMS was combined with SSRI treatment, and the placebo effect was marginal [95]. Although all RCTs included medicated patients, controlling for medication—specifically SSRIs—may optimize the aftereffects of HF rTMS. Indeed, SSRIs can enhance and prolong excitatory neuromodulation [69]. In addition, an ‘interactive’ model of rTMS mechanisms proposes that rTMS may not necessarily restore specific functions due to limited after effects, but rather, may “allow the brain to restore itself” [85]. rTMS may modulate a certain level of pathological activity and prime the neural system to become more adaptable to adjunct therapy and further excitatory modulations.

Analysis of symptom subdomains provided further insight. For HF rTMS, only left-sided application led to significant improvements in obsessions but not compulsions, but when controlling for depression, the effect held true for the left and right conditions together, but not for the left condition alone [89,92]. This finding suggests that the therapeutic effects of HF rTMS of the left DLPFC are driven by changes in depression, and that rTMS treatment of the left DLPFC alone is insufficient to produce clinical benefit for OC symptoms.

##### Right DLPFC

Mean symptom improvements were moderate from rTMS of the right DLPFC in randomized controlled contexts, with response rates of 20–50% for LF and 31–54% for HF protocols. Although response rates were similar for low and high frequency trials, direct comparison of frequency demonstrated within-patient superiority of LF at post-treatment that increased in magnitude three months later [103]. Placebo effects were relatively low, with the exception of one LF [93] and one HF trial [97], in which active and control groups showed comparable outcomes. Follow-up reports consistently demonstrated continued improvements four and six weeks after HF treatment [89,97,99], maintained at three months from LF but not HF treatment [103].

##### DLPFC General

Various DLPFC montages reported a larger antidepressant and anxiolytic effect compared to the anti-obsessive-compulsive effect [91,92,93,97,99,102,110,111]. This is compatible with a recent meta-analysis [139]. It is well established that excitatory stimulation of the left DLPFC or inhibitory stimulation of the right DLPFC has an antidepressant effect, and that both montages are an effective treatment for depression [140]. It could be stated that DLPFC applications have a strong antidepressant effect irrespective of laterality, frequency, and pathological state. Here, this effect most commonly occurred with LF (inhibitory) rTMS of the right DLPFC. The premise of targeting the DLPFC is also related to consistent beneficial clinical effects in numerous psychiatric conditions, playing a global role in decision-making and emotional regulation, rather than showing a specialized correlation to OCD pathophysiology. LF and HF rTMS of the right DLPFC led to similar outcomes immediately following treatment, which is likely driven by a primary antidepressant effect and secondary effect on obsessions and compulsions. Yet, LF rTMS of the right DLPFC led to more prolonged benefits, possibly due to a stronger antidepressant effect.

No predictors of response were identified [110,111]. Across studies, greater treatment dose (i.e., sessions and pulses) did not relate to greater efficacy; in fact, optimal outcomes were achieved with just 10 sessions and ≤1000 pulses per day [104,111]. This is contrary to the treatment efficacy–dose relationship of rTMS DLPFC application for depression [141] and highlights that stimulation protocols should not be extrapolated across diagnostic groups.

When post-treatment outcomes were reported, symptom suppression was always maintained or improved, which highlights a possible learning effect. Considering the fact that DLPFC applications may not directly modulate obsessions and compulsions, and greater dosage did not achieve greater response, a treatment regime that administers a moderate treatment dose combined with behavioral therapy that directly targets obsessions and compulsions may achieve optimal clinical efficacy across several global symptoms.

#### 3.6.2. OCD, Pre-SMA/SMA

Apart from the study of Pelissolo (2016), which reported a greater placebo effect than treatment effect, LF rTMS of the pre-SMA/SMA achieved responses in between 22–80% of patients across five RCTs, an open-label randomized trial and one retrospective report. Combined targeting of both OCD and MDD symptoms (pre-SMA and right DLPFC) showed an impressive response rate of 83% [111]. TBS of pre-SMA achieved a response in almost a third of patients, but the placebo effect was greater than the treatment effect [117]. Follow-up outcomes were reported in two studies that showed a slight improvement at two-week follow-up [107] or decline at three-month follow-up [100]. It was stated that responders were maintained at three-month follow-up from LF rTMS of SMA, but outcomes were not reported [90]. No discernible differences were identified between pre-SMA and SMA applications, and as such, outcomes were discussed jointly herein.

There was a treatment dose-efficacy association that became evident only after 20 sessions, with sustained improvements between 20–44 sessions and no reported adverse events. Within the cohort of Mantovani (2010), a small number of patients entered a second phase of 20 additional sessions, and response was achieved in all. Hawken (2016) administered 25 treatments and titrated down the regime, resulting in a response rate of 80% that was maintained six weeks later. In contrast, Gomes and colleagues found that 10 sessions of the same protocol did not maintain efficacy at three-month follow-up [100]. Furthermore, 24 maintenance sessions following 20 acute sessions enhanced improvements from 35% to 65% in a single patient [123]. Hyperactivity in the pre-SMA, particularly during inhibitory behavior, is a potential endophenotype of OCD [142]. Individuals with greater hyperactive CSTC likely require additional stimulation to regulate pathological activity and reach a threshold that alters behavior. Thus, 20 or more LF rTMS sessions of the pre-SMA may be required to reach response and maintain efficacy.

Insufficient and inconsistent information prevented us from identifying a relationship between rTMS effects for comorbid symptoms. Although articles did not report or demonstrate evidence that depression hindered OCD outcomes, a large group level analysis identified comorbid depression as a potential negative prognostic marker [118]. Notably, tailoring targets for both OCD and MDD symptoms may optimize outcomes for both conditions [111]. Psychiatric comorbidities other than MDD were reported in two studies, and it cannot be determined whether they impacted outcomes [108,112]. Mostly anxiety and depression outcomes showed a lack of correlation with OCD (YBOCS) outcomes [90,96] although OCD and anxiety symptoms did correlate within one RCT that showed high efficacy [100].

It can be concluded that LF rTMS of the pre-SMA/SMA can achieve high clinical efficacy for OCD, as confirmed by several randomized controlled investigations and clinical case series. If the most recent RCTs are considered (excluding that of Mantovani et al., 2010), response rates were consistently high (42–80%). It is possible that the methods and expertise of rTMS applications became refined over time, and recent trials represent a realistic response rate for pre-SMA/SMA applications. When comorbidities are present, additional targeting of comorbid symptom domains appears to have additional benefits. Longer-term treatment (≥20 sessions) with slow tapering off may be required for some patients to achieve a response and should be considered in stimulation protocols.

#### 3.6.3. OCD, OFC

Response rates between 25–44% were achieved from LF rTMS of left OFC, and efficacy was maintained at four- and eight-weeks following treatment [94,115]. A RCT showed greatest improvement in the active group at the two-week FU (21.4%), which was maintained at eight weeks FU (19.2%) but showed some loss of gains at 12-week FU (15.5%). LF deep TMS of right OFC led to 19% change, which was not maintained at one-month follow-up [101].

The study of Nauczyciel (2014) showed decreased metabolism in bilateral orbitofrontal lobes (pretreatment) to a greater extent in the (targeted) right hemisphere. Additionally, improvements in YBOCS were correlated with a decrease in right OFC activity. Despite limited response to ten sessions of LF deep rTMS of the right OFC, sufficient modulation occurred to induce changes in resting state activity. Within-group analysis showed a slight superiority of LF rTMS of the left OFC over LF rTMS of the SMA [118].

Although only four studies were identified, the number of sessions had a progressive positive relationship with efficacy, and baseline severity scores were relatively high compared to most trials targeting other brain regions. Two trials [115,118] showed efficacy (43–44% response) only after having extended their treatment protocols to 20 sessions. The two trials that achieved less positive change [94,101] used 10 or 15 sessions, and their patients also had high baseline YBOCS scores (~32 points).

Given the central role of a hyperactive OFC in OCD pathophysiology, it is reasonable to assume that less severe patients with hyperactivity of the OFC may respond more rapidly to rTMS, whilst more severe patients may require additional treatment sessions. In support of this notion, the number of previous medication trials (which is related to treatment duration or severity) was significantly lower in TMS responders in the trial of Kumar and colleagues [115]. A separate study found that, compared to responders, nonresponders had a significantly greater duration of illness, higher baseline YBOCS, were more likely have comorbid MDD, and had failed more medication trials [118]. These data suggest that effects of OFC rTMS are related to several aspects of illness severity and comorbidities.

#### 3.6.4. OCD, Other Prefrontal Targets

Investigations of HF rTMS of mPFC and ACC led to 44% and 45% response with marginal placebo effects within reviewed RCTs [114,116]; a 50% response was found when targeting the dmPFC alone [106]. These findings gain support from a large multisite trial, in which efficacy was maintained four weeks later [116]. Dunlop (2016) administered an additional 10 sessions to nonresponders, and 50% of them achieved response based on a high criterion (50% improvement). Also, LF rTMS of the medial PFC alone achieved 40% mean improvement [105]. Baseline clinical characteristics did not predict YBOCS outcomes [105,106].

Targeting the medial PFC led to consistent efficacy, despite heterogeneous stimulation protocols (10–30 sessions, 1200–3000 pulses), varying symptom severity (mean baseline YBOCS of 22.8–30.5), and complex psychiatric comorbidities (MDD, bipolar, anorexia nervosa, bulimia nervosa, PTSD, TS). It should be noted that all studies employed novel techniques, including neuronavigation and symptom provocation. Symptom provocation activates CSTC circuits [143] and is implemented on the basis that subsequent facilitatory stimulation may have additive effects.

#### 3.6.5. TS, Motor Targets

LF rTMS of the SMA showed 33–71% response and 17–34% change within open- and closed-label trials, except in a single pilot study, in which it did not achieve efficacy [130]. Theta burst stimulation was associated with a high response rate, yet the placebo effect was greater than the treatment effect [128]. One investigation showed no effect of targeting the pre-SMA using deep TMS [130]. Studies of LF rTMS of the premotor cortex or pre-SMA did not result in symptom improvement, suggesting too low a ‘dose’ of TMS for efficacy.

Notably, when efficacy was achieved, symptoms remained suppressed in the long term, i.e., from 3–6-months [126,127,131]. A previous meta-analysis contended that the daily and total dose of TMS was not related to clinical outcomes [27], but a treatment threshold likely exists, as two sessions was insufficient, and some patients required maintenance therapy (15 sessions) for response to be achieved or maintained [129,131]. There was heterogeneity in baseline severity (YGTSS between 20.6–64.7 on average, and 37–85 within case studies), although severity was not correlated with outcomes.

LF rTMS of the SMA was able to achieve high efficacy and long-term outcomes in a subgroup of TS patients and is recommended for clinical use. Further investigations into predictors of response are required, although response does not appear to be related to symptom severity.

#### 3.6.6. Other Conditions

LF rTMS of the pre-SMA led to a transient effect for skin picking, followed by deterioration [133]. One HD patient experienced benefit from LF rTMS of right DLPFC, which was associated with increased functional connectivity between the right DLPFC and vmPFC [134]. The data are too sparse to allow conclusions to be drawn.

#### 3.6.7. Polarity Dependent Effects

Low and high frequency protocols showed counterintuitive clinical outcomes. Theoretically, LF rTMS protocols should be optimal in suppressing the frontal-striatal hyperactivity that underlies OCRD. However, both LF and HF stimulation were effective for OCD, and HF stimulation was particularly effective for mPFC targeted alone or in combination with ACC. The mPFC and ACC play central roles in monitoring internal states and motivational drives and integrating emotionally salient information [144]. Although the mPFC and ACC are involved in the dorsal and affective CTSC loops, hyperactivity is not consistently associated with obsessions and compulsions. The favorable facilitatory effect on these regions may be due to their broad role in cognitive control and emotional regulation, which are supposedly enhanced in an obsessive-compulsive state.

#### 3.6.8. Novel Techniques

Novel techniques including deep coil, neuronavigation, and TBS were implemented in a few of the studies reviewed here [105,106,109,114,116,117,121,130]. Three out of five deep TMS studies showed efficacious outcomes and a low placebo effect for mPFC and ACC [105,114,116], while two showed marginal treatment effects for right OFC and SMA [101,130]. Deep TMS coils were developed to overcome the limited penetration depth of 1.5–2.5 cm from figure-eight coils to target deep neural structures, i.e., around 6 cm in depth [145]. In any neuromodulation scenario, there is a trade-off between focality and depth of penetration, owing to an exponential decay of the signal as a function of distance from the stimulation discharge [146]. The protocols that did not achieve efficacy from deep coils (yet efficacy was shown with figure-eight coils), targeted largely superficial (SMA) or deep structures (OFC), whereas efficacy was achieved by targeting medial structures (mPFC, ACC). Therefore, deep rTMS current may be too diffuse for regions in either close or far proximity and is better suited for use on regions of medial proximity.

Three studies administered continuous (inhibitory) theta burst stimulation. Although moderate treatment effects were found, placebo effects were comparable to [128] or even larger than [117] treatment effects. Stimulation protocols using TBS may require refinement or induce nontherapeutic oscillations for OCRD.

Neuronavigation techniques most often showed efficacious outcomes in four out of six studies [96,105,106,134]; however, superiority over standardized targeting has not been established for OCD.

Dual targeting may be more effective than single targeting. Donse (2017) targeted the SMA and right DLPFC (inhibitory rTMS), while Carmi (2018; 2019) targeted the mPFC and ACC (facilitatory rTMS); both achieved high efficacy. Importantly, the former montage was applied to a heterogeneous clinical group, and hence, is representative of OCD populations. Additionally, the latter montage was supported by a large multisite RCT. Although Kang (2009) did not achieve efficacy by targeting the SMA and right DLPFC, a much higher number of pulses was delivered (2400 vs. 1000) compared to those applied in the study by Donse (2017). Therefore, dual targeting should be further explored with consistent stimulation protocols that have already shown effectiveness.

### 3.7. DBS Results

DBS involves the placement of electrodes in the brain. These are stimulated by a battery-powered stimulator usually placed under the clavicle and produce a biphasic and high frequency electrical current that travels in and out of neurological substrates (cells, axons, dendrites, and glial cells), resulting in a small electric field of around 2.5–5 mm within deep neural structures. Stimulation of a specific target has widespread effects on neural circuits, depending on, among other factors, the orientation and size of activated nerve fibers and the cytoarchitectural organization of innervated neural populations [147].

The most well-established theory on the mechanisms of DBS action postulates that a ‘functional lesion’ occurs when the stimulation frequency is around twice the firing frequency of the neurons; as such, a circuit is ‘captured’, resulting in local inhibition [148]. Recent theories have been expanded to consider network models, and have recognized that local excitation, suppression of pathological firing, and plasticity mechanisms may also underpin the therapeutic effects of DBS [149].

Determining the ‘sweet spot’ of stimulation is complex. Potential sources of mechanical, technical, or human error related to surgery and postoperative programming can confound patient outcomes. Unlike applications for movement disorders, in which response is almost immediate and overtly observed, DBS therapy for psychiatric conditions often has a prolonged onset and more variable response.

Appropriate patient selection, accurate placement of the electrode, and effective programming are the major factors that contribute to DBS outcomes [150]. Programming is the only modifiable factor of therapy once the leads have been implanted and is of particular importance when electrodes are placed at the border of the targeted structure or have been misplaced. The overall aim of programming is to optimize clinical benefit, avoid side effects, and minimize current consumption [151]. Stimulation amplitude (constant or cyclic current or voltage) is the amount of stimulation delivered to the neural tissue and is the most adjusted parameter. The location and number of active contacts (or configuration) changes the volume of tissue activated (VTA). The monopolar configuration has a spherical current field, bipolar has a narrow oval current field, and multipolar has two current fields [147]. High frequency DBS is effective due to the time-locking parameters of axons, and 130 Hz is common based on a trade-off between efficacy and battery consumption. Pulse width variation in increments of 60µs is common, as it is the minimum pulse duration needed to initiate an action potential in a myelinated axon [151]. There are generally three programming phases. The initial visit is to screen for the optimal contact, often combined with anatomical (neuroimaging) information and intra-operative test stimulation observations. The early optimization phase aims to optimize the stimulation parameters and medication dose, which commonly involves titrating the stimulation amplitude. Then, patients are monitored around once a year, to check for unexpected worsening, battery consumption and troubleshooting.

One hundred and eighteen articles were screened for eligibility: 71 were included in the final synthesis, comprising 28 investigations for OCD, 42 for TS, and 1 for BDD. Forty-seven articles were excluded due to a lack of standardized assessment of primary symptoms (*n* = 16), reporting of primary outcomes in another article (*n* = 11), lack of pre- to post- operative outcomes (*n* = 5), adjunct therapy having been implemented (*n* = 4), the primary diagnosis not being an OCRD or was unclear (*n* = 4), previous DBS for Parkinson’s (*n* = 1), or presence of comorbid psychosis (*n* = 1). OCD investigations included 9 RCTs (six with an open-label extension), five open-label trials, two follow-up reports, one pilot study, seven case series, and four case reports. TS investigations included three RCTs with open-label extension, five open-label trials, five follow-up reports, one pilot study, three retrospective reports, seven case series, and 17 case reports. BDD investigations included a single case study. The final sample included, 153 OCD patients, 175 TS patients, and one BDD patient. 

OCD DBS targets included the anterior medial subthalamic nucleus, amSTN; anterior limb of the internal capsule, ALIC; bed nucleus of stria terminalis, BNST; supero-lateral branch of the medial forebrain bundle, sl-MFB; nucleus accumbens, NAc; inferior thalamic peduncle, ITP; ventral capsule/ventral striatum, VC/VS. TS DBS targets included the thalamus (Centromedian (CM) thalamus; centromedian-parafascicular complex (CM-Pfc) thalamic nuclei; centromedian nucleus- substantia periventricularis - nucleus-ventro-oralis (CM- SP- VOA); centromedian-parafascicular and ventralis oralis complex (CM-Pf- VOA)); globus pallidus internus, GPi (anterior globus pallidus internus (a-GPi); anterio-medial globus pallidus internus (am-GPi); posterolateral globus pallidus internus (pl-GPi); posteroventral globus pallidus internus (pv-GPi)); globus pallidus external, GPe; ALIC; NAc, and amSTN. BDD DBS targets included the VC/VS only. 

Table 12 and Table 13 show summary results of DBS investigations for OCD, Table 14 and Table 15 show summary results of DBS investigations for TS, and Table 16 shows summary results of DBS investigations for BDD. See Supplementary File S4 (S4) for classification of DBS targets.

#### 3.7.1. DBS Results for OCD

One RCT implanted two targets (four electrodes) per patient; DBS of the VC/VS, amSTN, and both targets achieved 53%, 45% and 60% mean improvement, respectively [168]. Two open-label studies compared two targets: NAc DBS led to 12–23% improvement, where-as BNST DBS led to 24–39% improvement [163,164].

Investigations of NAc DBS included three RCTs, one open-label trial, and three case reports. RCTs led to 13% and 51% symptom improvement [161,165], and long-term treatment (6–12 months) led to 12–33% improvement [161,162,167].

Investigations of ALIC DBS encompassed two RCTs, one trial with a staggered switch on, one long-term follow-up report, and two case reports. Closed-label investigations led to 20% and 43% improvement [153,157]; and long-term (1–9 years) treatment led to 43–67% improvement [153,159,160]. The ALIC was also targeted in the cohort of Mantione et al., (2014) through shifts in targeting, and achieved 43% improvement at 1 year [220].

Investigations into VC/VS DBS involved one RCT, one open-label trial with a long-term follow-up report, and three case reports. The RCT originally implanted the ALIC [153], and implemented a posterior shift in target to the VC/VS. A larger cohort from the same site as Nuttin (2003) achieved 42% mean improvement from closed-label treatment, and at three-year follow up, 39% symptom improvement was maintained [156].

Investigations of amSTN DBS involved a multisite RCT that resulted in 25% median improvement, and 51% mean improvement was reached at four-year follow up [158].

DBS of the ITP, slMFB, and thalamus were also targeted for OCD, 52%, 42%, and 9% mean change was respectively achieved per target [166,173,175].

#### 3.7.2. DBS for TS

One clinical care study and four case reports implanted different targets within the same cohort, or implanted patients with two targets. Stimulation of the thalamus, GPi, or both targets led to 45%, 32–78%, and 36–60%, respectively [186,204].

Thalamic DBS was investigated in two RCTs, three open-label trials (two with follow-up reports), two retrospective reports, and five case studies; outcomes of the closed-label phase of one RCT is not reported here due to the limited treatment duration of 7 days per condition. Closed-label treatment achieved 40% improvement, while open-label led to 19%, 44% and 55% improvements [179,183,187,191]. Sustained improvements were achieved across several cohorts with 30–73% change from 1–6 years of therapy [180,181,182,183,188,192,196].

Investigations into globus pallidus internus (GPi) DBS included two multisite RCTs, two open-label trials, one retrospective report, and 11 case series. Closed-label investigations led to 10% and 22% improvement [190,193]. Open-label treatment led to 14%, and 47% improvement in the short term [184,189], while long-term therapy (1–4 years) led to 40–63% change [185,193,194,195].

Four case studies each implanted one patient in the GPe, ALIC, NAc and amSTN achieving 71%, 25%, 41% and 92% symptom improvement, respectively [198,200,210,215].

#### 3.7.3. DBS for BDD

One patient achieved response (35% improvement) from VC/VS DBS [22]. 

### 3.8. DBS Discussion

The RoB assessment rated 46 articles as low risk, 16 as medium risk, and nine as high risk (S2). The quality assessment rated 19 articles as good, 44 as moderate and eight as poor (S3). Only 11 out of 71 articles were RCTs, and 35 were case reports, which meant a randomized control aspect and group level analysis was not present in almost half of the patients included here. Furthermore, only half (36) of the articles reported on more than one time-point, which limits interpretations regarding the duration and pattern of response. Within the bias assessment, there were multiple deviations from the intended protocol, including DBS explants or switch off, and closed-label conditions ending early. It was reported that 18 (11.7%) OCD patients and 12 (6.8%) TS patients had their devices switched off or explanted due to limited/no efficacy or even worsening in some instances; a further three (1.7%) TS patients underwent repositioning. Also, five RCTs had patients that ended the closed-label phase early. It is possible that not all cases of device switch off, explant, or repositioning were captured.

Adverse events included transient psychiatric symptoms, particularly hypomania, increased anxiety, deterioration of mood and suicidal thoughts, which were generally resolved with programming adjustments. There were seven suicide attempts, and one completed suicide [157]. Battery depletion was rarely reported on but seemed to occur between 5–22 months in OCD cohorts [153,154,170] and was reported to occur at 24-months for one TS patient [210].

There was large heterogeneity in protocols, and no comparable protocols were identified, making a meta-analysis not possible. To elaborate, surgical procedure, target trajectory, programming method, stimulation location, comorbidities, follow-up duration, and closed-label conditions varied greatly. Comorbidities reported in OCD trials included MDD (*n* = 17), personality disorder (*n* = 4), bipolar (*n* = 3), PD (*n* = 2), GAD (*n* = 1), panic disorder (*n* = 1), BDD (*n* = 1), TS (*n* = 1), yet was most often not reported (*n* = 44). Comorbidities reported in TS trials included OCD (*n* = 37), MDD (*n* = 29), ADHD (*n* = 15), GAD (*n* = 5), dystonia (*n* = 3), panic disorder (*n* = 1), personality disorder (*n* = 1), and was not reported in a further 26 cases.

#### 3.8.1. OCD, NAc Target

Modest and consistent change was achieved from NAc DBS with 21–33% improvements from open-label treatment. Yet Barcia (2019) demonstrated that high efficacy of closed-label DBS (85% full response, 100% partial response) is achievable [165]. This was attributed to optimization of the stimulation contact, and thus, stimulation of the ideal anatomical structure on a patient-specific basis. The protocol implemented a three-month condition for each contact and reported outcomes on the best one. Although other investigations may trial each contact in a monopolar review or exploratory programming across several days, this is likely insufficient to determine the true therapeutic effect [222]. Thus, optimization of programming appears to influence efficacy.

Further supporting the findings, the RCT that showed minimal improvements applied predefined and global stimulation parameters for the closed-label phase [161]. Although refining the stimulation parameters prior to the closed-label conditions has implications for blinding, applying the same therapeutic settings to diverse neurobiological patient profiles that have electrodes implanted in slightly different anatomical positions limits therapeutic benefits. Further, the importance of optimizing therapy within RCTs is rarely discussed and is proposed herein as a critical factor for outcomes. It should also be considered that Huff (2010) applied unilateral DBS, which may have also affected outcomes.

In a different protocol, eight months of open-label DBS led to 25% initial improvement. Following 24 weeks of adjunct CBT, this increased to 46%, and a subsequent closed-label phase (with CBT) of active DBS led to 1.9% deterioration while sham led to 45% deterioration- reaching baseline severity [162]. This protocol highlights the strength of DBS therapy, such that open-label effects can be maintained during closed-label conditions with CBT, yet CBT alone was not sufficient to maintain previous DBS therapeutic effects. This protocol also demonstrated the importance of optimizing therapies in a staged manner. Programming was assessed fortnightly across an eight-month period and adjusted if necessary. Once stimulation optimization had been achieved, CBT was introduced. This allowed an extended period for DBS response and a transition to CBT once patients were receptive to behavioral therapy.

Long-term outcomes were modest, with a maximum of 33% improvement at 12 months or last (8–54 months) follow-up [164,167]. The case study that experienced deterioration had comorbid mild TS, which may have hindered efficacy [169].

#### 3.8.2. OCD, ALIC Target

Three study sites showed efficacious long-term outcomes from ALIC DBS, such that 58–67% of participants achieved response at one year, which was maintained at 6–9 years [153,159,160,220]. Substantial change was achieved within three-months [153,178], with subsequent gradual improvement.

However, in comparison to these studies, the cohort of Abelson (2005) did not show comparable efficacy within both open- and closed-label phases. The RCT that achieved high response levels implemented an extensive programming regime that assessed all parameters and contact configurations across weeks to months prior to blinded phases [153]. The study of Abelson (2005), which showed inferior results to other RCTs, conducted exploratory programming across just 3–8 days before the blinded phases. This study was a pilot and supposedly involved the first implants at that site; thus, limited experience with DBS patients may have contributed to moderate outcomes. Additionally, limited programming likely hindered patients reaching response.

It is well established that the duration of clinical response to DBS is slow within psychiatric conditions in comparison to movement disorders [222,223]. Differing behavioral effects can occur across minutes to months, which influences the ability to optimize stimulation. The number of stimulation combinations is vast and the assessment of efficacy of each is laborious.

ALIC DBS responses appeared to rely on programming adjustments, particularly stimulation amplitude. High intensities up to 8.5 V or 10.5 V were applied for chronic ALIC DBS across all trials with the exception of the most recent investigation [220], and the patient that declined between 4–12 months [177] had a lower stimulation intensity (2–4 V). A case series showed a ceiling effect of clinical response at six months which coincided with optimization of stimulation [178]. Also, it was reported that one patient did not reach response until eight months, coinciding with a second contact being activated [159]. Fayad (2016) showed that responders were maintained between one to 6–9 years with comparable stimulation parameters [158]. Thus, ALIC DBS may require particularly extensive programming, requiring amplitude titration across 6–12 months, at which point stimulation parameters and clinical efficacy should stabilize.

#### 3.8.3. OCD, VC/VS Target

Two RCTs (one with an absence of sham) showed rapid improvements in blinded phases (42% and 53% mean improvement) [156,168], and further improvements in the longer term, with ultimate response rates of 62–83%. The greatest magnitude of response was achieved within three months, although optimization of treatment, including CBT, further enhanced DBS outcomes.

Luyten (2016) reported outcomes depending on the location of the chronic active contact; BNST DBS led to superior outcomes with 50% improvement and 80% response, compared to ALIC DBS, that led to 22% improvement and 17% response. Activation of both the BNST and ALIC with multipolar configuration had a mean improvement of 66% and 100% response rate but was trialed in just three patients. Many fibers from the PFC—including the ACC—transverse through the ALIC and are part of the ventral capsule (VS) of the VC/VS; also, VS and NAc are terms which are used interchangeably to refer to a confluence between the putamen and caudate [224]. Thus, stimulation of the VC/VS likely modulates the ALIC and/or closely connected structures. Stimulation of both BNST and ALIC through monopolar stimulation causes two adjacent current spreads, which is also likely to reach neighboring structures. Therefore, active contacts within the ALIC may cause a current spread that overlaps to other regions, and optimization of stimulation within this region may rely on a diffuse current spread over neighboring regions.

Controlled comparison of amSTN and VC/VS showed the superiority of VC/VS, with 83% response in patients who had been implanted with leads in both targets; stimulation of both slightly increased efficacy to 60% (from 53% from VC/VS alone), but no further responders were achieved [168]. Owing to the heterogeneous surgical and clinical practices across sites, there is great value in assessing targets within the same center. Variance in clinical practices that may confound patient outcomes are minimized in this approach. Tyagi (2019) implemented within-patient comparison of two targets, which further minimized variance in individual clinical and anatomical characteristics.

There is within-patient evidence that targeting the VC/VS is superior to amSTN DBS, and efficacious outcomes from VC/VS DBS are likely attributed to the stimulation of additional structures- BNST and ALIC.

Randomized controlled outcomes were similar across different programming protocols. An extensive programming phase was implemented across several months prior to the closed-label phase in the multisite cohort study of Luyten and colleagues [156]. The smaller cohort of Tyagi et al. (2019) employed two weeks of programming adjustments prior to each phase, but there was no sham control [168]. These studies suggest benefits from extensive programming for VC/VS DBS prior to closed-label phases, with enhancement of long-term outcomes.

The Luyten (2016) cohort showed that the majority of improvements were achieved within three months, but further improvements were seen even years after implantation (e.g., at four years, there was a 66% improvement).

#### 3.8.4. OCD, amSTN Target

One cohort showed progressive and high efficacy through targeting amSTN: 51% at four years, with 75% being full responders [13]. An earlier report from this cohort recognized errors in targeting, as four leads missed the target, and 9/33 active contacts were not within the STN [158]. However, the follow-up report noted that both responders and nonresponders had leads placed within the target [13]. Another patient showed a similar pattern with 29% and 92% change at 3 and 36 months, respectively [215]. Tygai (2019) implanted patients with both amSTN and VC/VS leads, which are discussed above (within VC/VS); briefly, closed-label outcomes of amSTN DBS achieved 45% improvement and 50% response, and VC/VS DBS led to greater efficacy. Owing to consistent slow response from targeting the amSTN, a closed-label period of three months is likely insufficient to achieve full response for amSTN DBS therapy. This delayed pattern of response should be considered in clinical care and research methodologies.

#### 3.8.5. OCD, BNST Target

Across three cohorts, BNST DBS achieved 43% improvement during closed-label conditions 225, 39% at six months of open-label treatment [163], and 24% at long-term (8–54 months) follow-up [164]. Although the cohort of Luyten et al. (2016) initially targeted the VC/VS, long-term outcomes were reported for each location of the active contact- ALIC or BNST. At last follow-up (54–171 months), BNST DBS led to 80% response, and the majority of the cohort (15/24) had active contacts within the BNST (outcomes discussed above in ALIC). Although direct targeting of BNST was limited, incidental investigations though target shifts provided valuable insight and robust evidence of high efficacy.

#### 3.8.6. OCD, Other Targets

A recent pilot study of ITP DBS showed efficacy in five patients [166], and a separate study found that superior-lateral MFB DBS led to efficacy in two patients [173]. Thalamic target DBS was not effective for OCD in two patients who had previously received NAc DBS; depression ratings worsened [175].

#### 3.8.7. Optimized Localization

Across studies, OCD outcomes were variable and dependent on fine-tuning of stimulation location and other parameters. Most of the OCD investigations targeted the striatal regions (ALIC, VC/VS, BNST, NAc), and there was robust evidence to support targeting dorsal and posterior regions. In one trial [165], YBOCS improvement was significantly greater in caudate (E2,3) compared to NAc (E0,1) contacts. Caudate contacts were within the ALIC and likely also stimulated the VS, yet the NAc was still reported as the anatomical target. The other RCT that targeted the NAc did not reach efficacy and applied active contacts within the NAc with fixed stimulation parameters [161]. Patients receiving NAc DBS often had chronic activation of multiple contacts, likely leading to the stimulation of other regions outside the NAc itself [161,167]. Yet, when the optimal location across the NAc trajectory was determined (within and outside the NAc), multipolar stimulation was not required to reach high efficacy [165]. Although studies commonly targeted the NAc, stimulation parameters showed that stimulating the NAc alone led to suboptimal therapy.

The patients reported by Mantione (2014) were included in the largest cohort study thus far reported, comprising 70 patients [226]. Although the report was outside the cut-off date for the current review, we provide a summary of the main findings. The initial 16 patients reported by Mantione (2014) were targeted with two contacts within the NAc, but it was later revealed that the position of the electrode shifted so that just one contact was within the NAc, and three contacts were within the ventral ALIC. The patients all had chronic active contacts within the ventral ALIC (not the NAc), which corresponds with the ventral capsule of the VC/VS, and mean symptom (YBOCS) improvement of 40% and response in 52% were achieved. Therefore, there is evidence from several investigations at several sites that ALIC stimulation is superior to NAc stimulation.

Investigations of other striatal regions identified even more precise optimal target. A large multisite cohort originally targeted the ALIC with the most ventral contact in the NAc [153]. Again, the target shifted, i.e., posterior, ventral, and medial to the VC/VS, and at the junction of the anterior capsule, anterior commissure, and posterior ventral striatum, with the most ventral contact in the BNST [155,156]. The shift had a dramatic effect on outcomes, lifting an initial 33% response rate to 78% and 75% in the latter cohorts with the optimized target. The target was originally reported as the ALIC [153], then the VC/VS as the optimized target [154,155], and finally, as the BNST or ALIC- depending on the chronic active contact [156]. Most patients were receiving chronic BNST stimulation (*n* = 15), while some received ALIC stimulation (*n* = 6). A similar pattern occurred in a separate cohort, in which the original target was the ALIC [159] but shifted to the VC/VS in a follow-up report [160]. Importantly, through extensive localization refinement, regions posterior and medial to the ALIC (including the VC/VS and BNST) demonstrated superior outcomes. This effect is consistent across and within cohorts, and within patients.

Further support for this notion can be found in the extremely high stimulation amplitudes for ALIC DBS (up to 10.5 V), which indicates a large VTA is required for efficacy, and likely involves stimulation of several neighboring regions. Activation of multiple contacts was often required for NAc DBS [161,167], which creates several overlapping VTAs and indicates stimulation of superior structures.

Across all published studies, the anatomical target, and often the chronic active contact, were specified (E0–E3); yet the precise positioning of the active contact(s) in relation to the target was rarely reported. Owing to all the possible sources of positioning errors and differences in anatomy, patients will be implanted at slightly different locations, as determined by postoperative imaging. It cannot be assumed that leads are placed in a comparable trajectory across patients. Depending on the chronic contact(s) within a single lead trajectory, several different regions may be activated.

Reporting of the precise anatomical localization of the active contact and stimulation parameters (including configuration) is required for progress to occur the field, and these aspects of therapy are critical for refining DBS therapy.

#### 3.8.8. Optimized Stimulation Parameters

Although implementing predefined stimulation parameters during closed-label phases is advantageous for blinding, it likely limits efficacy. Across targets, it was identified that a lack of programming was a major determinant of suboptimal therapy [157,161]. Trials that had an extensive optimization phase in the weeks prior to closed-label conditions all achieved high efficacy [153,156,165,168]. The extent of programming for one RCT was not clear [158].

The largest reported cohort of DBS for OCD [226] included an extensive optimization phase with assessments every two weeks, which likely contributed to efficacious outcomes. Refinement of suboptimal stimulation by highly experienced clinicians allows nonresponders to reach efficacy at all points of clinical management, even three years after surgery [227,228]. The fine-tuning of programming can be burdensome; it is reliant on the expertise of the clinician and the fluctuating state of the patient. Yet, it is contended that a ‘one size fits all’ approach is inappropriate within this context of a complex therapy and pathophysiology.

#### 3.8.9. Functional Connectivity Insight

Recent neuromodulation perspectives have shifted away from focal stimulation of brain nuclei, focusing instead on the modulation of distributed brain networks through analyses of connectomics [229,230,231]. The concept of ‘circuitopathies’ is not new, yet recently enhanced MRI capabilities have made it possible to identify white matter tracts and connectivity pathways. Fiber tracking analysis has consistently shown that activation of fibers from the target nuclei to the PFC (medial, lateral, dorsolateral) is correlated with good response across different OCD DBS targets and cohorts [221,229,232]. This indicates that multiple targets modulate a shared network that similarly affects OC symptoms. Indeed, the connectomic approach was able to explain 40% of variance, thus presenting itself as a promising biomarker [221].

Furthermore, the cortico-thalamo-basal ganglia network has recurrent excitatory and inhibitory loops, and different DBS targets have similar and unique connectivity patterns. The striatal target of the ventral ALIC and VS involves fibers in the OFC and ACC. The BNST contains fibers from the PFC to thalamus, and likely involves the modulation of ALIC fibers. The VS contains a complex mixture of myelinated fibers from the OFC, ACC, amygdala, and BNST among other connections, and stimulation of the VS. will likely modulate ALIC fibers. The medial STN receives fibers from the PFC, ACC, and other prefrontal regions, depending on the functional subdivision, and stimulation will likely have a secondary effect on the VS. [224]. Thus, the ALIC, VS and STN are topographically organized to receive OFC and ACC innervations, connecting to distinct subcortical pathways. Therefore, it is proposed that different OCD DBS targets modulate a shared subcortical-prefrontal network that can similarly affect OC symptoms. Also, DBS targets likely activate a specialized circuit that affects comorbid symptoms (mood, anxiety, reward, avoidance) or cognitive functions (inhibition, memory, attention) to varying degrees.

This notion is further supported by diffusion tractography analysis, which was shown to be able to predict clinical efficacy [165,168,220]. Tractography of the optimal contact within amSTN and VC/VS leads showed that both had connections to the OFC, and additional distinct tracts [168]. amSTN and VC/VS had comparable effects on OC symptoms, but divergent outcomes on cognition and mood. Fibers of the amSTN connect to the dorsal ACC, DLPFC, and medial forebrain bundle, and stimulation was associated with change in cognitive flexibility, while stimulation of the VC/VS (which is connected to the mediodorsal thalamus, amygdala, hypothalamus and habenula) resulted in changes in mood. Even within a single target (ALIC), the anatomical stimulation site cannot predict clinical response, while fiber connectivity can [220]. To elaborate, clinical response was correlated with active contacts which were closer to one fiber branch (superior-lateral MFB) than another (anterior thalamic radiation), yet the anatomical location of the electrode in a standardized space did not predict response.

Insight from connectome modelling elucidates the mechanisms underlying neuromodulation on a network level and makes it possible to target neural networks in order to predict DBS response, heralding a more objective and personalized treatment approach. Tractography analysis can also benefit non-responders by repositioning the electrode based on predictive fibers or selecting the stimulation site to prevent numerous time-consuming programming trials. Although limitations exist regarding the use of fiber tracking to target DBS, there are significant advantages in targeting symptom specific (not disease specific) circuits depending on the patient profile [229,230]. Future work should incorporate this approach throughout the pre- and post- operative stages of targeting and localizing DBS electrodes.

#### 3.8.10. Other Clinical Management Considerations

DBS-mediated effects on distinct obsessive and compulsive symptom domains were reported in some trials [13,161,162,164,233,234]. Improvements were comparable for both domains, except for one amSTN trial, that had a 76% improvement in obsessions and 55% improvement in compulsions [233].

Two trials showed CBT could further enhance open-label treatment within a short period [162,168], and sometimes it was reported that CBT was initiated or resumed after 3–12 months of open-label therapy [154,172,220]. It was proposed that DBS may be able to break the association between stimuli and obsessions (i.e., anxiety), and that a second break in the association between obsessions and ritualistic behaviors (i.e., inhibition) occurs through CBT [162]. Although this did not translate to quantitative outcomes within the review, further investigation is warranted.

#### 3.8.11. TS, Thalamus Target

Closed- and open-label investigations of thalamic stimulation showed high efficacy in TS, although reports of the former were limited. One RCT achieved 67% response; eight follow-up reports across six cohorts showed sustained long-term efficacy, with 30–73% improvements and 60–100% response across 1–6 years of therapy, excluding a trial of cycling therapy, that alternates between off and on periods of therapy.

Clear efficacy was achieved in all reports (≥60% response) except for one small open-label trial [187,188]. Delayed switch on implemented in the protocol did not have a statistical effect on outcomes. However, this was the only study to implement scheduled cycling, which alternated between periods of stimulation on and off to account for the intermittent nature of tics and varying symptom profiles. Although scheduled cycling may benefit battery life, it was shown to be inferior to constant DBS of the CM thalamus.

Progressive improvements appeared within CM thalamic applications from three months to six years across three sites [181,182,183,187,188], but a cohort that received ventral anterior and ventrolateral thalamic DBS reached ay ceiling effect at six months [191].

#### 3.8.12. TS, Globus Pallidus Internus Target

Open-label and case studies showed consistent therapeutic responses from GPi DBS in TS, while randomized controlled investigations showed only modest outcomes (10–22% improvement). Long-term open-label outcomes across four cohorts achieved 41–63% mean improvements (50–75% response rate) from 12 to 46 months of treatment.

Open-label therapy [184,185] demonstrated that response was largely achievable within just one month, with subsequent gradual improvement over the ensuing eight to 46 months. The majority of trials included several follow-ups, which all showed continued improvement from up to four years of therapy [184,185,189,190,193,194].

Although most trials targeted the anterior (limbic) subdivision of the GPi, similar response rates were achieved from posterior (69%) and anterior (71–75%) GPi DBS. Case reports showed mean improvements of 52% (11–93%) for posterior GPi DBS [45,197,205,211] and 68% (20–95%) for anterior GPi DBS [205,209,212,213]. Also, outcomes from a case series [205] that implanted both targets suggested superiority for the anterior GPi. It appears that targeting the limbic functional division is standard practice, showing a slight superiority, while the motor division is targeted in patients with more complex or motor predominant symptoms, including self-injurious behavior and comorbid dystonia [235].

#### 3.8.13. TS, Other Targets

One patient was implanted per GPe, ALIC, STN, NAc targets. Although response was achieved in all except ALIC DBS, conclusions cannot be drawn from such limited evidence. Further, NAc DBS required activations of all four contacts with a stimulation intensity of 7 V, thus posing limitations on battery duration and indicating possible suboptimal placement [200].

#### 3.8.14. Optimized Localization

For TS, the thalamus and GPi were predominantly targeted, and both achieved efficacy. Blinded outcomes were greater for thalamic DBS over GPi DBS, although RCT investigations were limited for both targets. Long-term response rates showed superiority for thalamic DBS (60–100%) over GPi DBS (50–75%). Within-patient comparisons of targets were limited: GPi showed superior outcomes in three patients, whilst in another, CM-PF thalamus was found to be slightly superior [199,204].

Like this review, a database registry study of 185 TS patients showed comparable outcomes between CM thalamic DBS and GPi DBS, but superiority of the anterior GPi over the posterior GPi [236]. A previous review of the VTA across several sites showed that the most stimulated region of the GPi was the amGPi [237]. Further, the anterior GPi has higher connectivity to regions that are positive predictors of response compared to the posterior GPi [235]. Therefore, clinical and connectivity evidence favors the anterior-limbic functional region of the GPi unless the patient profile is suited to an alternative region.

#### 3.8.15. Optimized Stimulation Parameters

The extent of programming optimization prior to closed-label conditions did not appear to affect outcomes. The trial that achieved high efficacy implemented three weeks of programming prior to blinding [183], whereas the other two trials that achieved moderate outcomes implemented one week [190] or one month [193] of programming. It should be noted that during open-label treatment, recurrence of symptoms occurred in some patients, indicating that long-term monitoring and adjustments may be necessary [180,209].

Open-label programming varied from four weeks to 12 months. Although such disparities in clinical management may theoretically impact outcomes, no clear relationship was identified between programming within open- and closed-label conditions and clinical outcomes. Also, TS outcomes had greater consistency compared to OCD outcomes.

#### 3.8.16. Other Clinical Management Considerations

Apart from one study that reported a relationship between lower baseline disease severity and better outcomes [191], we found no reports supporting a relationship between baseline severity and clinical response to DBS in TS. Some patients with very severe symptoms achieved a dramatic response, while some with lesser severity exhibited treatment resistance. A more striking predictor of response for TS was that younger age (20) was associated with clinical response for thalamic and GPi DBS, with improvements of between 37–100% [184,186,189,192]. A previous review also found younger age and lower disease severity at implantation to be associated with better outcomes [238]. However, a separate review found that median time to response was not impacted by age, despite the fact that lower age at implantation was associated with higher baseline YGTSS [237]. Thus, younger age at implantation may be predictive of response when baseline severity is low.

Investigation of motor and phonic tic subdomains did not demonstrate a clear pattern across thalamic and GPi targets. Thus, there was comparable change for motor and phonic tics (within 5–15%) [180,181,183,184,185,187,190,191,192,194,195].

Owing to the intermittent nature of tics, and evidence that tic-generating networks function at varying time points [239], it has been contended that continuous DBS stimulation may not be necessary for tic relief. However, cycling of DBS has been associated with inferior outcomes in comparison with continuous DBS [187,188].

A number of case reports have suggested that comorbid OCD is a positive prognostic factor for DBS in TS. Stimulation of the GPi in comorbid patients achieved improvements of 67%, 85%, 90%, 94%, 94% and 95% [201,212,213]. When targeting the thalamus, comorbid patients achieved individual improvements of 60%, 69%, 80%, 82%, 83% [206,207,208]. However, the effect of comorbid OCD was not addressed in clinical trials, even though comorbidity was common. DBS modulates several networks that underlie tic and obsessive-compulsive behaviors, as well as mood and cognition; thus, it is likely that alleviating one aspect of impairment will have complimentary effects on other aspects. Indeed, several TS patients with comorbid OCD/obsessive-compulsive behavior achieved suppression of both conditions from DBS targeted for TS [200,201,207,209,211,213,217].

DBS treatment for TS showed a more consistent pattern of response than OCD applications, and minimal or no placebo effect from sham [183,190]. Across all investigations, just three study sites did not achieve efficacy [45,187,188,205]. Despite the consistency of response identified in this review, a previous in-depth analysis showed large variability in YGTSS change, i.e., 46.7 ± 29.7, and a YBOCS change of 21.1 ± 52.9 from DBS therapy for TS [237]. Thus, whilst clinical response was consistent across TS investigations, the variance in change is large, indicating that the identification of prognostic factors may allow patients to achieve even higher levels of efficacy.

## 4. Conclusions

### 4.1. ECT

A recently published expert report on new developments in evidence-based management of OCD [240] recommends ECT only for acute treatment of comorbid conditions (e.g., depression, psychosis). Currently, ECT is usually considered for OCD only after a number of other treatment interventions, i.e., as a last resort, when rapid improvements are necessary, or if a life-threatening psychiatric state is present [37]. Previous systematic reviews of ECT for OCRD have concluded that there is a lack of unequivocal evidence to support the efficacy of ECT. The current review found greater response rates than previous reviews [24,26] that adopted more lenient definitions of response, and included a greater spectrum of obsessive-compulsive conditions.

The current study identified response rates of 79% and 100% from ECT in OCD and TS cases, respectively. Although investigations involved a small number of patients, and there were no randomized or sham controlled investigations, the magnitude of effect was large considering the patients’ level of severity and treatment resistance. Yet, without randomized placebo-controlled trials, valid recommendations cannot be made. The current review implemented stricter inclusion criteria than previous reviews, and necessitated standardized assessments, which resulted in the inclusion of fewer articles. Pooling together heterogeneous samples with biased methods and reporting may have previously obscured clinical interpretations. Cohort studies with standardized assessments following treatment and improved reporting of clinical characteristics are necessary to establish more objective guidelines regarding the potential value of ECT in OCRD.

### 4.2. tDCS

In agreement with previous systematic reviews and expert opinions [23,241,242], we found tDCS outcomes for OCRD to be modest and heterogeneous. This has previously been attributed to differences in stimulation protocols and clinical characteristics of patients. Our findings accord with those of Jacobson et al. (2012), i.e., that the observed heterogeneity of outcomes in part reflects the diversity of polarity effects [243]. Cathodal stimulation was the most often applied polarity in tDCS investigations, based on the assumption of inhibitory modulation. Yet, this may not be the true mechanism of action. Insight into the precise effects of consecutive applications of a relatively high dose (2 mA) tDCS on local cortical excitation and diffuse connectivity in psychiatric states is necessitated to understand behavioral changes and refine stimulation protocols.

Recently, da Silva et al. (2019) modelled the spatial distribution of electrical fields of the montages associated with tDCS in OCD patients [241]. Two prominent diffusion patterns were identified: (1) electrical fields focused within different PFC regions [52,62,64,71], and (2) electrical fields diffused across regions within and outside the PFC [51,53,55,59,60,63,65]. Consideration of these diffusion patterns within the context of the response patterns identified here showed that montages with a focused current spread within the PFC resulted in immediate symptom improvement, although not always meeting response criteria. An antidepressant effect was also common. In contrast, montages with diffuse modulation across the brain tended to show a delayed onset of response, in which symptom improvement was minimal immediately following treatment, but improved at follow-up. Thus, the distribution of tDCS neuromodulation may influence the pattern (onset and duration) of response, and other factors (i.e., stimulation dosage and clinical characteristics) may influence whether a particular patient responds to tDCS. This has implications for individualized therapy, notably for scenarios that necessitate immediate symptom suppression.

Stimulation dose (but not frequency) had a positive relationship with improvements with tDCS, in OCD patients. Protocols did not plan more than 20 sessions, but extended therapy led to further improvement in nonresponders [57]. This highlights the notion that a subgroup of patients may respond at a slower rate and require more than 20 sessions.

Protocols should also consider that by increasing the regions targeted, a rapid response may be achieved within a few sessions [55,61,64]. Finally, despite the small number of studies, tACS was shown to be able to induce changes with minimal stimulation dose [54].

Recent reviews of tDCS in OCD have failed to reach definitive conclusions or propose clinical recommendations [23,72,242,244], in part reflecting the fact that there were no RCTs included in previous systematic reviews [23,242]. A review of previous case studies of tDCS for TS proposed the pre-SMA as an optimal target, but no further recommendations were reached [25]. Previously, neither response rates per target nor patterns of response have been established; rather, these methods rely on ‘improvement’ or statistical change on the primary outcome measure, which limits clinical interpretations. From the studies reviewed here, we conclude that for OCD patients (1) twice daily anodal tDCS of the pre-SMA/SMA can achieve response in at least a third of individuals; (2) predictors of response should be investigated, (3) high dose tDCS of the DLPFC warrants randomized controlled investigations into optimal dosage and laterality; and (4) daily cathodal tDCS of the left OFC should be investigated in patients without a high level of treatment resistance, implementing an extended treatment regime and long-term follow-up. For TS patients, daily cathodal tDCS of the pre-SMA/SMA at low stimulation intensity (~1.4 mA) warrants further investigation.

There are several limitations that cannot be controlled across studies, including disparities in medication, resting brain state, clinical characteristics (level of treatment resistance, illness duration, and symptom profile), precision of targeting, and multiple and varied stimulation parameters (polarity, stimulation intensity, duration, and frequency, and return electrode placement). Controlled methodology, standardized protocols, and understanding of cortical aftereffects are required to establish more robust clinical recommendations.

### 4.3. TMS

A previous meta-analysis [245] concluded that the greatest treatment effects from TMS for OCD were associated with targeting the right DLPFC, then bilateral DLPFC, and then left DLPFC; investigations for OFC and SMA were too sparse to include a ranking. Further, no differences between HF and LF protocols were identified, and intensities of 100% RMT were favorable. More recently, Rehn (2018) concluded that LF rTMS of the SMA was optimal for OCD, but that bilateral DLPFC and right DLPFC targets were also effective [139]. Treatment effects were maximal at 12 weeks, and greater for anxiety and depressive symptoms than primary OCD symptoms.

For TS, previous reviews have identified younger age and comorbid ADHD/OCD as positive prognostic factors, and the SMA as a favorable target [27,82]. Yet, a recent meta-analysis concluded that rTMS is no more effective than placebo for the treatment of TS [27].

The current review agrees with previous reviews and provides further insights. It is concluded that LF rTMS of the pre-SMA/SMA yields the highest response in up to 80% of OCD patients, and up to 70% in TS patients, and that increased treatment sessions should be investigated for maximal response.

For OCD patients, LF rTMS of the right DLPFC carries sustained efficacy. Outcomes on OC symptoms are likely mediated through antidepressant and anxiolytic effects. Recent investigations have highlighted the potential for dual targeting, activating/priming neural systems prior to stimulation, and HF stimulation. Specifically, HF rTMS of the mPFC and ACC yielded robust findings that should be subjected to long-term follow-up. Also, regions that are not directly implicated in OCD pathophysiology (DLPFC, mPFC, ACC) but which have a more global function in cognitive and emotional control consistently showed high efficacy. Yet, an optimal frequency was not identified, as efficacy was achieved from both excitatory and inhibitory rTMS. Regions directly implicated in OCD pathophysiology (pre-SMA/SMA, OFC) may require a relatively high number of treatment sessions (20) to normalize hyperactivity and thus alter behavioral manifestations.

No clear predictors of treatment response were identified for rTMS in OCD. It is proposed that neuroimaging should be implemented for the purpose of identifying abnormalities in functional connectivity rather than in precise regions. This approach may yield more optimal patient identification and target selection. Further, considering the importance of the resting brain state on rTMS outcomes [85], careful consideration of the state of patients prior to and after treatment may reduce response variability and improve outcomes. Studies generally have not reported methods to control for this effect. For example, priming the pathological brain state [114,116] and controlling pharmacological interventions [95], which both alter cortical excitability, provided consistent outcomes. Novel techniques (neuronavigation, deep TMS and TBS) did not show clear superiority over conventional protocols, but symptom provocation during rTMS appeared to enhance outcomes in OCD.

For TS patients, long-term efficacy for tic suppression is achievable with LF rTMS of the SMA, and thus, is recommended for clinical practice. Longer therapeutic trials should be considered for initial nonresponders. Predictors of response were not identified in TS, in part due to a lack of consistent protocols and randomized controlled investigations. Tic generation is underpinned by hyperactive motor pathways and hypoactive control pathways of the CSTC loops [18]. Like OCD applications, LF rTMS of the pre-SMA/SMA and HF rTMS of cognitive control regions may be effective in TS, but these procedures have not been subjected to clinical trials. Investigations into rTMS for other OCRD were very limited, and conclusions cannot be drawn.

### 4.4. DBS

One previous systematic review contended that there is insufficient evidence to conclude the existence of an optimal DBS target for OCD [31], while another proposed the NAc as the optimal target [29]. Similarly, for TS, no superior target has been determined, although the inferiority of NAc/ALIC DBS [238] and comparable efficacy of the thalamus and GPi DBS have been argued in international database studies [236,237]. Experts in the field [31,246,247] have proposed that a multisite registry is necessary for enhanced DBS management and the standardization of protocols. Such TS registries [236,248] have already allowed progress to be made in the research and clinical care discussed herein [235,237].

The current review identified novel findings that depart to some extent from previous reviews. For OCD treatment, NAc DBS led to modest changes, with 50–70% reaching *partial* response from 12 months of therapy and around 30% improvement over longer periods. Optimization of therapy through contact selection and adjunct behavioral therapy can enhance NAc DBS efficacy and achieve 85% response. ALIC DBS led to rapid and dramatic efficacy with further changes seen at long-term follow-up, with response in at least 60% of patients being achieved. Varying patterns of response were observed from ALIC DBS, which appears to rely on stimulation intensity and fine-tuning of programming. VC/VS DBS can achieve high efficacy and shows superiority over other striatal regions and the amSTN. Rapid improvements occurred in closed-label conditions from VC/VS DBS, and response continued to improve, with long-term response rates of 80% or more. amSTN investigations were limited, but 75% response was achieved from long-term treatment, supporting further applications for OCD. Direct investigations of BNST are recommended, as there is evidence for superiority over other targets (ALIC, NAc) and long-term efficacy, with up to 80% response.

There is extensive behavioral and programming evidence that the VC/VS and BNST encompass the DBS ‘sweet spot’ of the striatum; as such, these regions have been identified as optimal targets for OCD therapy. Extensive programming prior to closed-label conditions is critical to achieve full DBS effects. Also, CBT is likely to optimize DBS effects and should be incorporated into care.

For TS treatment, a smaller number of patients have been implanted than for OCD, but with less variance in targets, with thalamus and GPi being consistently chosen, even though the targeted subregions did show variation across studies. Continuous CM thalamic DBS led to consistent and sustained efficacy for tic suppression, with 60–100% response rates. Response usually occurred within months, with continued improvements for up to six years following implantation. Despite limited efficacy from blinded treatment, there is robust open-label support for GPi DBS in TS. Effects occured rapidly (1–3 months) with gradual improvement, such that years after surgery, response rates of 50–88% can be achieved. Efficacy from affective and motor divisions of the GPi were consistent, but there was greater evidence to support targeting of the anterior-limbic functional division in the long-term treatment of TS.

Treatment for TS appeared to be less reliant on programming optimization compared to OCD patients; however, this does not exclude the necessity to optimize programming for TS, or refute the hypothesis that greater efficacy may be achievable by doing so. Furthermore, TS patients tend to achieve a more rapid response than those with OCD, and show continued improvement following years of therapy. Younger age appears to be a predictor of DBS response for TS. For TS patients with comorbid OCD, stimulation of TS targets will likely achieve symptom suppression for both conditions.

DBS mediates change in obsessive-compulsive behavior through modulation of a shared network, which is consistent across targets and conditions. In comparison, DBS effects on tics occur through distinct networks depending on the stimulated region. Connectivity analysis, but not anatomical location, was sufficient to predict response for OCD and TS. There was an absence of behavioral or clinical predictors of response. Connectome modelling is a data-driven yet complex approach to identify biomarkers. Thus, fiber connectivity analysis is an important evolving field that should be considered in anatomical targeting and postoperative management.

The complexities of DBS-mediated recovery and the effects on different determinants of functioning are not fully captured within quantitative outcome measures [222,249,250]. Some patients do not achieve response on the primary outcome variable, yet choose to maintain stimulation due to improved anxiety, depression, and/or quality of life. Other patients achieve response yet struggle with newfound realizations and the ‘burden of normality’. Future reviews may consider a more integrated clinical and global functioning picture; however, this is beyond the scope of the current review.

Across all techniques, there was a scarcity of research for OCRDs (other than OCD and TS), and thus, conclusions could not be made for the application of neurostimulation therapy across all OCRDs.

## Figures and Tables

**Table 1:** Summary results of electroconvulsive therapy for OCD.

Study (Country)	N (m:f)	Study Design	Illness Duration-Main Obsession	Baseline YBOCS	Rx	Stimulation Parameters			YBOCS % Change from Pre-Treatment		Comments/Conclusions
						Sessions	Target	Intensity	Post treatment	Follow up	
Maletzky 1994 (USA) [38]	32 (13;19)	Retrospective review	✕	21.7 ^	✓	3.5 (average)	Bilateral fronto-temporal	21.2 J	42.3% * +	35.4% * +	OC symptoms improved by 42.3% at 5 days following treatment, and efficacy was maintained by 35.4% at 12-month FU. Depression symptoms improved by 48% immediately following treatment, improvements maintained by 24% at 12 months follow up (FU).
Tomruk et al., 2010 (Turkey) [39]	2 (0;2)	Case series	P1: 13y-sexuality, guiltP2: 1y-persecution, sexuality	P1: 40 P2: 40	✓	P1: 7 P2: 8	✕	✕	P1: 90% + P2: 95% +	✕	OC symptoms drastically improved in 2 patients with comorbid depression and psychotic features after short term ECT. Changes in other symptom domains or FU outcomes were not reported. It was stated that P2 remained in remission at 15-month FU.
Liu et al., 2014 (China) [40]	3 (2;1)	Case series	✕	P1: 29 P2: 27 P3: 21	✓	P1: 8 P2: 3 P3: 4	Bilateral frontal	0.88–0.92 A	P1: 44% + P2: 58% + P3: 47.6% +	✕	OC, depression and anxiety symptoms improved by 44–58%, 50–62%, 37–50%, respectively across the 3 patients. It was claimed that patients remained stable at long term FU (up to 4 years), and P3 was symptom free at 4 year FU.
Manhas et al., 2016 (India) [41]	5 (✕)	Subgroup of an interventional study	✕	28.6 ± 3.7	✕	6–12	Bilateral	✕	43.7% +	14.6%	The study was part of a larger cohort that assessed numerous psychiatric conditions, OCD patients are reported here. According to a global impression criterion, 3 out of 5 patients (60%) were responders following treatment, and 1 responder remained so at 3 and 6 month FU.
Agrawal et al., 2018 (India) [42]	1 (1;0)	Case study	6y-contamination, pathological doubt and slowness	35	✓	a-ECT: 8 m-ECT: 6	✕	✕	a-ECT: 65% + m-ECT: 54% +	a-ECT: 0% m-ECT: 0%	OC symptoms showed a rapid improvement of 65% after 8 sessions, ECT treatment was stopped due to cognitive deficits, and symptoms returned to baseline. 6 additional ECT sessions led to a 54% improvement, once treatment stopped OC symptoms deteriorated again to baseline. The patient deteriorated beyond baseline levels at further FU.
Aggarwal et al., 2019 (India) [43]	3 (1;2)	Case series	P1: 3m-contamination P2: 5y-sexual nature P3: 2.5y-contamination	P1: 24 P2: 22 P3: 33	✓	P1: 12 P2: 12P3: 8	Bilateral frontal	✕	P1: 85% + P2: 63% + P3: 12%	✕	OC symptoms improved drastically in 2 out of 3 patients. The non-responder had less ECT sessions and a higher baseline YBOCS. It was stated that P1 and P2 maintained response at 6-month FU and P3 was lost to follow up.

A, amps; a-ECT, acute electroconvulsive therapy; J, Joule; M-ECT, maintenance electroconvulsive therapy; P, participant; Rx, patients continued taking prescribed medication; SIB, self-injurious behavior; y = years; + = clinically significant change from baseline; * = statistically significant change from baseline; ✓ = criterion applies; ✕ = not reported. ^ Maleztsky et al., 1994 [38] used the Maudsley Obsessive Compulsive Inventory (MOCI) as the main outcome.

**Table 2:** Summary results of electroconvulsive therapy for TS.

Study (Country)	N (m:;f)	Study Design	Illness Duration-Main Symptoms	Baseline YGTSS	Rx	Stimulation Parameters			YBOCS Outcomes % Change from Pre-Treatment		Comments/Conclusions
						Sessions	Target	Stimulus intensity	Post treatment	Follow up	
Morais et al., 2007 (Brazil) [44]	1 (1;0)	Case report	20y-motor and vocal tics, echolalia, coprolalia, SIB	85	✓	23	Bilateral temporal	504 mC	100% +	✕	The protocol involved 8 acute (2/week), 13 continuation (titrated down across 5 months), and 2 maintenance (1/ month) ECT sessions. Complete remission of tic and depressive symptoms occurred, with no relapse at 8 month FU.
Dehning et al., 2011 (Germany) [45]	1 (1;0)	Case report	30y-motor and vocal tics, SIB, palilalia, coprolalia, OCB	50	✓	37	Unilateral	50 mC	100% +	✕	The extensive treatment protocol lasted for 5 years, including 14 acute (3/week), and 23 maintenance ECT sessions (titrated down from monthly to bi-annually). Complete remission of tics occurred, with no relapse at 5 month FU.
Rajashree et al., 2014 (India) [46]	1 (0;1)	Case report	12y-motor and vocal tics, coprolalia, OCD	84	✕	6	✕	✕	96% +	✕	6 ECT sessions were effective in reducing TS and OC symptoms, the patient experienced complete remission of OC symptoms. Efficacy was maintained at 3-month FU.
Guo et al., 2016 (USA) [47]	1 (1;0)	Case report	8y-SIB, coprolalia	90	✕	13	Bilateral	800 mA	83% +	✕	13 ECT sessions were effective in reducing TS symptoms, an improvement in depression was claimed, yet outcomes not reported.

mA, milliamps; mC, milicoulombs; OCB, obsessive compulsive behavior; Rx, patients continued taking prescribed medication; SIB, self-injurious behavior; y = years; + = clinically significant change from baseline; ✓ = criterion applies; ✕ = not reported.

**Table 3:** Summary results of transcranial direct current stimulation for OCD.

Study (Country)	N (m:f)	Study Design	Baseline YBOCS	Rx	Stimulation Parameters			YBOCS Outcomes % Change from Pre-Treatment			Comments/Conclusions
**OCD**					Polarity: target Polarity: return/ target	Amplitude, duration	Sessions	Post treatment	Follow up	Responders (Criterion, if reported)	
Bation et al., 2016 (France) [51]	8 (2;6)	OL, single arm trial	29.0 ± 5.8	7/8	Cathode: left OFC Anode: right cerebellum	2 mA, 20 min,	10 (2/day)	22% *	26.5% +	3 months: Full: 37.5% Partial: 62.5% (35% full, 25% partial)	Cathodal tDCS over left OFC was effective for a subset of individuals. Clinical efficacy was maintained and greatest at 3 month FU. Subjective improvement was greater (45.6%) than objective assessment (26.5%). Depression symptoms improved by 19.7%. The only medication free patient was a non-responder.
Dinn et al., 2016 (Turkey) [52]	5 (1;4)	OL, single arm trial	156.8 ± 74 ^	✓	Cathode: right OFC Anode: left DLPFC	2 mA, 20 min	15 (5/week)	23% *	1.7% (deterioration)	✕	Anodal tDCS over left DLPFC, and cathodal tDCS over right OFC was partially effective post treatment, but effects did not remain. Despite deterioration of OC symptoms at 1 month FU, depressive symptoms remained improved by 31.7%.
D’urso et al., 2016b (Italy) [53]	12 (5;7)	RCT, 2 phases; cross-over or repeated design	✕	11/12	Cathode or anode: pre-SMA Anode or cathode: right deltoid	2 mA, 20 min	20 (10 per phase, 5/ week)	C-C: 20.1% * A-C: 14.9% A-A: 6.6% C-A: none	✕	✕	If symptoms worsened the polarity was switched, otherwise participants remained in the randomly allocated condition. Cathodal was superior to anodal, but clinically significant change was not achieved within conditions.
Klimke et al., 2016 (Germany) [54]	7 (6;1)	OL, single arm, pilot study	31.9 ± 5.6	6/7	Fronto-temporal tACS^, 40Hz	650 µA, 20 min	8–20 (3/week)	52% * +	✕	Full: 85.7% Partial: 100% (35% full, 25% partial)	tACS over bilateral frontotemporal regions, was effective for OC symptom suppression, improvements were between 28–86% indicating all reached at least a partial response, and all but 1 patient reached a full response.
Najafi et al., 2017 (Iran) [55]	42 (19;23)	OL, single arm trial	29.1 ± 2.7	✓	Cathode: bilateral DLPFC Anode: parietal/temporal/occipital regions	2–3 mA, 30 min	15 (5/week)	65.6% +^,^*	1M: 68.3%+ 3M: 81.5%+	✕	Three cathodal DLPFC contacts and 3 anodal contacts were highly effective for OC symptoms. A rapid and maintained improvement occurred, that continued to improve 3 months after treatment.
Bation et al., 2019 (France) [56]	21 (9;12)	RCT, active or sham	A:29 ± 4.5S:29.4 ± 6.6	✓	Cathode: left OFC Anode: right cerebellum	2 mA, 20 min,	10 (2/day)	A: 4.7% * S: −2.3%	A: 10.6% S: 4%	A: 20% S: 9% (35%)	The same stimulation protocol as Bation et al., 2016 in a RCT was effective in a small number of patients, but group mean change was minimal. The level of treatment resistance was significantly associated with OC symptom improvement in the active group; responders had lowerresistance.
Godwa et al., 2019 (France) [57]	25 (21;4)	Phase 1: RCT, active or sham. Phase 2: OL	A:25.8 ± 4.8 S:27.3 ± 5.2	✓	Cathode: right supraorbital region Anode: pre-SMA	2 mA, 20 min	10 (2/day)	Phase 1: A:22% # S:12%	Phase 2: A: 7.7–15.7% S: 11.7–8.4%	A: 33% S: 0% (35%)	Anodal tDCS over pre-SMA had statistical and clinical significantly greater improvement than sham. Another 5 days of OL treatment was offered for non-responders. Those that entered phase 2 from the active condition had a further improvement (7.7% to 15.7%), those that entered from sham had a reduction in improvements (from 11.7% to 8.4%)
Kumar et al., 2019 (India) [58]	20 (11;9)	OL, single arm trial	31.6 ± 4.9	✓	Cathode: SMA Anode: right occipital area	2 mA, 20 min	20 (2/day)	16.5% *	✕	15% (35%)	Cathodal tDCS over SMA was effective in a small number of patients. Anxiety and depressive symptoms showed greater improvement of 32% and 47% respectively.

M = month; Rx, patients continued taking prescribed medication; + = clinically significant change from baseline; * = statistically significant change from baseline; ✓ = criterion applies; # = statistically significant change compared to control condition; ✕ = not reported; µA = microampere. ^ Dinn et al., 2016 [52] the main outcome assessment used was the Obsessive-Compulsive Inventory (OCI). ^ Klimke et al., 2016 [54] implemented transcranial alternating current between Fp1-T3 and Fp2-T4 (10–20 coordinates).

**Table 4:** Summary results of transcranial direct current stimulation for OCD case studies.

Study (Country)	N (m:f)	Study Design	Illness Duration-Main Symptoms	Baseline YBOCS	Rx	Stimulation Parameters			YBOCS Outcomes % Change from Pre-Treatment		Comments/Conclusions
						Target, return electrode	Amplitude, duration	Sessions	Post treatment	Follow up	
Volpato et al., 2013 (Italy) [59]	1 (1;0)	Double blind, case report	23y-cleanliness, symmetry, hoarding, slowness, MDD, GAD	22	✓	Cathode: left DLPFC Anode: posterior neck	2 mA, 20 min	10 (5/week)	0%	✕	Cathodal tDCS over the left DLPFC had no effect on OC symptoms. Depression and anxiety symptoms were in the moderate range at baseline and respectively improved by 34% and 17.8% after active; and improved by 14% and declined by 12% following sham. Following a 2 week wash out, 10 sessions of inhibitory rTMS of primary motor cortex had no effect on OC symptoms within a sham-controlled context. Anxiety and depression increased by 30% and 12% respectively after active TMS.
Mondino et al., 2015 (France) [60]	1 (0;1)	Case report	15y-hoarding, contamination	36.5	✓	Cathode: left OFC Anode: right OFC	2 mA, 20 min	10 (2/day)	1.3%	26%	Cathodal of the left OFC and anodal of the right OFC did not lead to immediate suppression of OC symptoms, nor at 2 week FU. A delayed and partial response (26%) emerged at 1 month FU.
Narayanaswamy et al., 2015 (India) [61]	2 (1;1)	Case report	P1: 5y-aggression, sexuality, blasphemous, SAD P2: 3y-sexuality, aggressive	P1: 25 P2: 30	✓	Cathode: right supraorbital region Anode: left pre SMA/SMA	2 mA, 20 min	20 (2/day)	P1: 40% + P2: 46.7% +	P1: 52% + P2: ✕	Anodal tDCS over pre-SMA and SMA twice daily was effective in reducing OC symptoms. It was stated that efficacy was maintained at 1-, and 2-month FU, yet FU outcomes at 1 week for P1 were only reported.
Goradel et al., 2016 (Iran) [62]	1 (01)	Case report	1y-religious, no compulsions	23	no	Cathode: left OFC Anode: right OFC	2 mA, 20 min	10 (1/day)	56.5% +	65.2% +	Cathodal and anodal tDCS over left and right OFC were effective in reducing OC symptoms; majority of improvement (43%) occurred within the first 5 days of therapy, and a further improvement was seen at 2-week FU. Depression and anxiety symptoms improved by 56% and 40%, and also showed greatest change at 2-week FU, with 87% and 100% improvement, respectively, which were in the moderate-severe range at baseline.
D’urso et al., 2016a (Italy) [63]	1 (0;1)	Case report	6y-contamination	34	no	Cathode-anode: right deltoid Anode-cathode: pre-SMA	2 mA, 20 min	20 (10 per condition, 5/week)	A: 11.7% (deterioration) C: 29.4%	32%	Anodal tDCS over pre-SMA led to deterioration of OC symptoms. The polarity of tDCS was then switched which led to a partial response, and further improvement at 3 month FU.
Hazari et al., 2016 (India) [64]	1 (1;0)	Case report	10y-aggressive, sexual, previous suicide attempt	23	✓	Cathode: right supraorbital region Anode: pre-SMA/SMA,	2 mA, 20 min	8 (2/day)	69.5% +	✕	Anodal tDCS of pre-SMA and SMA twice daily was effective in reducing OC symptoms in a patient that had been previously treated with ECT, and tDCS but relapsed following both treatments. The patient also experienced improvement in depressive and anxiety symptoms by 69% and 62.5%, respectively. It was not reported whether relapse occurred again.
Silva et al., 2016 (Brazil) [65]	2 (1;1)	Case series (pilot data from RCT)	P1: 21y-symmetry, ordering, GAD P2: 22y-contamination, aggression, psychotic symptoms	P1: 38 P2: 40	✓	Cathode: pre-SMA Anode: left deltoid	2 mA, 30 min	20 (5/week)	P1: 0% P2: 17.5%	P1: 18.4% P2: 55% +	Cathodal tDCS over pre-SMA did not have a clinically significant effect on both patients, yet 6 month later, 1 reached clinical response. Depression and anxiety symptoms improved by 50% in P2 and were not reported in P1.

GAD, generalized anxiety disorders; SAD, social anxiety disorder; y = years; + = clinically significant change from baseline; ✓ = criterion applies; ✕ = not reported.

**Table 5:** Summary results of transcranial direct current stimulation for TS.

Study (Country)	N (m:f)	Study Design	Illness Duration-main Symptoms	Baseline YGTSS	Rx	Stimulation Parameters			YBOCS Outcomes % Change from Pre-Treatment		Comments/Conclusions
						Target, return electrode	Amplitude, duration	Sessions	Post treatment	Follow up	
Mrakic-Sposta et al. 2008 (Italy) [66]	2 (0;2)	Blinded, sham controlled cross over case series	P1: 13 y P2: 20 y	P1: 59 P2: 66	✕	Cathode: left M1, Anode: right deltoid	2 mA, 15 min	5 (per condition, 5/week)	P1-A: 30.3% P1-S: 5% P2-A: 10.6% P2-S: 5%	✕	Cathodal tDCS over the left motor cortex did not lead to clinically significant improvement for 2 TS patients; 1 achieved a partial response of 30% improvement. Subjective improvement of general wellness was much higher-132% and 450%. There was minimal or no placebo effect.
Carvalho et al., 2015 (India) [67]	1 (1;0)	Case report	10 y	76	✓	Cathode: pre-SMA Anode: right deltoid	1.425 mA, 30 min	10 (5/week)	41%+	44% +	Cathodal tDCS over pre-SMA was effective in reducing tics, efficacy was maintained 6 months after treatment.
Eapen et al., 2017 (Australia) [25]	2 (0;2)	Case series (pilot data from RCT)	✕	P1: 83 ^ P2: 34 ^	✕	Cathode: SMA Anode: right deltoid	1.4 mA, 20 min	18 (2–3/week)	P1: 34% P2: 20.5%	P1: 43.3% +P2: 29.4%	6 weeks of cathodal tDCS over SMA led to partial clinical response in 2 TS patients. Following cathodal tDCS, 3 weeks of sham was administered, outcomes scores did not change. Further, improvement at 3 month FU occurred.
Behler et al., 2018 (Germany) [68]	3 (2;1)	Case series	All-vocal and motor tics P1: 45y-OCD P2: 4y-psychotic symptoms, SIB P3: 4y-OCD	P1: 29 P2: 38 P3: 36	P2 P3	Cathode: Pre-SMA/ SMA Anode: right neck	2 mA, 30 min	10 (2/day)	P1: 34% P2: 13.2% P3: 5.6%	✕	Concurrent cathodal tDCS over the pre-SMA and SMA led to partial response in 1 TS patient; however, YGTSS outcomes were contrary to tic counts which showed an increase in all patients by 18–200%. OC symptoms improved by 83% in P1, yet declined by 20% in P3.

P, participant; Rx, patients continued taking prescribed medication; SIB, self-injurious behavior; y = years; + = clinically significant change from baseline; ✓ = criterion applies; ✕ = not reported. ^ Eapen et al., 2017 [25] the main outcome assessment used was the Adult Tic Questionnaire (ATQ).

**Table 6:** Summary results of transcranial magnetic stimulation for OCD.

Study (Country)	N (m:f)	Study Design	Baseline YBOCS	Rx	Stimulation Parameters			YBOCS Outcomes % Change from Pre-Treatment			Comments/Conclusions
					Target	Pulses, sessions, frequency	RMT, frequency, coil	Post treatment	Follow up	Responders (Criterion, if reported)	
Alonso et al., 2001 (Spain) [88]	18 (6;12)	RCT, active or sham	A: 24 ± 5.3 S: 25.6 ± 6.1	13/18	Right DLPFC	20 min, 18 (3/week)	100%, 1 Hz, Circular coil	✕	A: 14% S: 1%	A: 20% S: 12.5% (40%)	Inhibitory rTMS of right DLPFC led to marginal improvement, and 1 more responder than the sham condition at 4-week FU.
Sachdev et al., 2001 (Australia) [89]	12 (9;3)	RCT, left or right DLPFC	L: 22.5 ± 6.2 R: 27.2 ± 8.9	10/12	Left DLPFC, Right DLPFC	1500, 10 (5/week)	110%, 10 Hz, Figure 8 coil	L: 25.9% R: 37.4%	L: 26.6% R: 55.8% * +	L: 33.3% R: 33.3% (40%)	Excitatory rTMS over the right DLPFC had greater mean symptom improvement than left DLPFC, which increased in magnitude at 1 month FU, although responders were comparable between groups. There was no statistical effect of laterality. Patients had a high level of treatment resistance, with an average of 5.2 previous medication trials.
Mantovani et al., 2006 (Italy) [90]	7 (✕)	OL single arm, pilot study	36.4 ± 7.5	✓	SMA	1200, 10 (5/week)	100%, 1 Hz, Figure 8 coil	28.5% *	✕	60% (40%)	OC outcomes for 5 OCD and 2 comorbid OCD/TS patients are reported here (comorbid patients withdrew after 1 week and are not included in responders, tic outcomes are reported below). Inhibitory rTMS of SMA was effective for 3 out of 5 OCD patients.
Prasko et al., 2006 (Czech Republic) [91]	33 (21;12)	RCT, active or sham	A: 29.82 ± 5.8 S: 23.42 ± 4.9	✓	Left DLPFC	1800, 10 (5/week)	110%, 1 Hz, Figure 8 coil	A: 23.8% S: 15.8%	A: 28.2% S: 27.7%	✕	Inhibitory rTMS of left DLPFC was slightly more effective than sham following treatment, yet at 2-week FU improvements were comparable. Baseline YBOCS was statistically significantly higher in the active group.
Sachdev et al., 2007 (Australia) [92]	18 (8;10)	Phase 1: RCT, active or shamPhase 2: OL	A: 25.8 ± 5.7 S: 23.9 ± 9.9	13/18	Left DLPFC	1500, 10 per phase (5/week)	110%, 10 Hz, Figure 8 coil	✕	✕	A: 30% S: 25% OL: 33% * (40%)	Excitatory rTMS of left DLPFC, led to similar response rates in active, sham and open label conditions. All patients received 10 closed label and 10 open label sessions.
Kang et al., 2009 (Korea) [93]	20 (17;3)	RCT, active or sham	A: 26.5 ± 5.6 S: 26.3 ± 4.0	✓	Right DLPFC, SMA	2400, 10 (5/week)	100%, 1 Hz Figure 8 coil	A: 8.6% S: 6.8%	A: 10.9% S: 12.9%	A: 20% S: 20% (25%)	Sequentially applied LF rTMS over right DLPFC and SMA was ineffective for OC symptoms, slight improvements occurred at 2 week FU.
Ruffini et al., 2009 (Italy) [94]	23 (✕)	RCT, active or sham	A: 32.2 ± 6 S: 31.4 ± 6.9	✓	Left OFC	✕, 15 (5/week)	80%, 1 Hz, Figure 8 coil	A: 18.9% # S: 6.6%	A: 21.4% # S: 7.6%	A: 25% full, 50% partial S: 0% full, 14% partial (35% = full) (25% = partial)	Inhibitory rTMS of left OFC was effective in a group of patients. YBOCS scores were statistically different between groups after 3 weeks of therapy until 10 week FU.
Badawy et al., 2010 (Egypt) [95]	60 (29;31)	3 arm trial; active (A), active + SSRI (A+), sham (S)	A: 22.65 ± 4.4 A+: 25.8 ± 4.8 S: 22.95 ± 3.6	A+	Left DLPFC	✕, 15 (5/week)	20 Hz, ✕	A: 8% A+: 20.1% * S:5%	✕	A:25% A+:55% S:5% (40%)	The active and sham group were medication naïve patients, and the active group taking SSRIs had a previous poor response to SSRI. Excitatory rTMS over left DLPFC was most effective for SSRI medicated patients (A+ group).
Mantovani et al., 2010 (USA) [96]	18 (7;11)	Phase 1: RCT, active or shamPhase 2: OL	A: 26 ± 5.4 S: 26.7 ± 5.5	13/18	SMA	1200, 20 sessions per phase (5/week)	100% 1 Hz, Figure 8	A: 25.3% S: 11.9% A-OL: 18% S-OL: 2.7% (deterioration)	✕	A: 22.2% S: 11% A-OL:100% S-OL: 12.5% (25%)	Inhibitory rTMS of SMA led to greater efficacy than sham. 4 from active and 8 from sham entered the open label; 20 additional treatments led to a cumulative improvement of 49% in active, and response in all 4 patients, which remained at 3 month FU, yet a small deterioration occurred in patients that entered from the sham group.
Sarkhel et al., 2010 (India) [97]	42 (23;19)	Single blind, non-randomised trial, active or sham	A: 25.7 ± 3.9 S: 23.6 ± 3.7	✓	Right DLPFC	800, 10 (5/week)	110% 10 Hz, Figure 8 coil	A: 9.4% S: 7.3%	A: 19.4% S: 17.6%	✕	Excitatory rTMS of the right DLPFC led to marginal change, the active and sham groups achieved comparable outcomes, and symptoms improved in both groups at 4 week FU.
Kumar Chadda 2011 (India) [98]	12 (5;7)	OL single arm trial	26.17 ✕	✓	SMA	1000, 15 (5/week)	100% RMT, 1 Hz Figure 8 coil	34.3% *	✕	✕	Excitatory rTMS of the SMA led to a mean symptom improvement of 34.3%.
Mansur et al., 2011 (Brazil) [99]	27 (13;14)	RCT, active or sham	A: 30 ± 3.8 S: 29.5 ± 5.0	✓	Right DLPFC	2000, 30 (5/week)	110%, 10 Hz Figure 8 coil	A: 10.8% S: 7%	A: 16.6% S: 9.8%	A: 30.7% S: 14.2% (30%)	Excitatory rTMS of right DLPFC led to response in almost a third of patients, yet mean change was minimal and not significantly different to change in the sham group.
Gomes et al., 2012 (Brazil) [100]	22 (9;13)	RCT, active or sham	A: 36.4 ± 3.2 S: 31.8 ± 3.5	✓	Pre-SMA	1200, 10 (5/week)	100%, 1 Hz, Figure 8 coil	A: 42% + S: 16%	A: 34.8% + S: 6.2%	A: 42% S: 12% (25%)	Inhibitory rTMS of the pre-SMA reached clinical response in almost half of patients, and the placebo effect was small. Symptom improvements were somewhat maintained at 3 month FU.
Nauczyciel et al., 2014 (France) [101]	19 (5;15)	RCT, cross over design	A: 32 ✕ S: 32 ✕	✓	Right OFC	1200, 10 sessions per condition (2/day)	120%, 1 Hz, Double cone coil (deep TMS)	A:18.7%# S: 6.2%	A:3% S: 0%	✕	Inhibitory deep rTMS of right OFC had minimal change, and improvements were lost at 1 month FU. Yet, OC symptom improvement was statistically greater in the active condition.
Xiaoyan et al., 2014 (China) [102]	46 (30;16)	RCT, active or sham	A: 24.5 ± 6.3 S: 23.4 ± 5.7	✕	Bilateral DLPFC	648–872, 10 (5/week)	80%, intrinsic αEEG	A:31.8%+ S: 15.1%	A: 29.7%+ S: 18.3%	A: 36% S: 0% (25%)	Individualised α frequency rTMS of DLPFC was effective for over a third of patients. At 1 week follow up, improvements were maintained and 24% of patients in the active group remained as responders. Obsessions, and anxiety but not compulsions, improved statistically greater in active compared to sham.
Elbeh et al., 2015 (Egypt) [103]	45 (30;15)	RCT; LF, HF, or sham	LF: 26.7 ± 6.5 HF: 25.4 ± 4.7 S: 24.9 ± 5.7	41/45	Right DLPFC	2000, 10 (5/week)	100% LF: 1 Hz HF: 10 Hz Figure 8 coil	LF: 44.9% * + HF: 27.1% * S: 5.6%	LF: 41.2% * + HF: 10.2% S: 8.4%	✕	Inhibitory rTMS of right DLPFC had statistically and clinically greater improvement in OC and anxiety symptoms compared to excitatory rTMS and sham. Efficacy was maintained at 3-month FU for inhibitory but not excitatory rTMS.
Haghighi et al., 2015 (Iran) [104]	21 (12;9)	RCT, cross over design	A-S: 30.4 ± 6.5 S-A: 30.1 ± 8.1	✓	Bilateral DLPFC	750, 10 per condition (5/week)	100%, 20 Hz, Figure 8 coil	A-S: 37.1% * +, 33.8% * S-A: 0.5%, 26.8% *		A: 54% full, 72% partial S: 0% (35% = full) (25% = partial)	Sequentially applied excitatory rTMS of left then right DLPFC led to statistical and clinical improvement. No order effects of treatment were detected. A large majority of patients reached partial response, and over half reached full response from the active condition, and none did so following sham.
Modirrousta et al., 2015 (Canada) [105]	10 (✕)	OL single arm trial	22.8 ± 3.1	7/10	mPFC	1200, 10 (5/week)	110%, 1 Hz, Double-cone coil (Deep TMS)	39.4% * +	✕	✕	Inhibitory rTMS using a double coil and neuronavigation to locate the mPFC led to 40% symptom reduction and consistent improvements between 23–76.5%.
Dunlop et al., 2016 (Canada) [106]	20 (4;16)	OL single arm trial	30.5 ± 4.3	✓	dmPFC	3000, 20–30 (5/week)	120%, 10 Hz, Figure 8 coil	39.6% *	✕	50% (50%)	Non-remitters were offered an additional 10 sessions (30 in total), the mean number of sessions was 21.3±4.3. Excitatory rTMS using neuronavigation of the dmPFC led to response in 50% of patients with a strict criterion, and extended protocol. Responders had a mean improvement of 67%.
Hawken et al., 2016 (Turkey, Bulgaria) [107]	22 (11;11)	Multi-site RCT, active or sham	28.0 ± 4.5	21/22	SMA	20 min, 25 (5/week, 4 weeks; 3/week, 1 week; 2/ week, 1 week)	110%, 1 Hz, Figure 8 coil	A:40% * + S: 5.7%	A:44.2% * + S: 16.3%	A: 80% S: 8.3% (25%)	LF rTMS over SMA was effective for a large proportion of patients, greatest improvement occurred at 2 week FU, and maintained at 6 week FU (scores not reported). The 2 non-responders in the active group had deterioration in OC symptoms; all others reached sub-clinical to moderate levels of symptoms.
Pallanti et al., 2016 (Italy) [108]	50 (26;24)	RCT, rTMS or TAU	TMS: 30.2 ± 2.5 TAU: 31.4 ± 2.5	✓	SMA	1200, 15 (5/week)	100%, 1 Hz, Figure 8 coil	TMS: 30.6% * + TAU: 18.8% *	✕	TMS: 68% TAU: 24% (25%)	Inhibitory rTMS of SMA was more effective than anti-psychotic medication.
Pelissolo et al, 2016 (France) [109]	36 (✕)	RCT, active or sham	A: 30.2 ± 4.2 S: 28.6 ± 4.6	✓	pre-SMA	1500, 20 (5/week)	100%, 1 Hz, Figure 8 coil	A: 7.9% S: 10.1%	✕	A: 10% S: 20% (25%)	Inhibitory rTMS using neuronavigation of pre-SMA was not effective for OC symptoms, change was marginal and greater in the sham group.
Seo et al., 2016 (Korea) [110]	27 (14;13)	RCT, active or sham	A: ~33 S: ~33	✓	Right DLPFC	1200, 15 (5/week)	100%, 1 Hz, Figure 8 coil	A: 10.7±8.2 ^ S: 3.7±3.7 ^	✕	A: 50% S: 23.1% (25%)	Inhibitory rTMS of right DLPFC led to response in half of patients compared to almost a quarter in the sham group. Group mean symptom change was reported as absolute change in YBOCS scores.
Donse et al., 2017 (Netherlands) [111]	22 (15;7)	OL study as standard clinical care	26.76 ± 5.71	✕	SMA, right DLPFC (in MDD patients)	1000, 10 (sessions/ week varied)	110%, 1 Hz, Figure 8 coil	42% * +	✕	83.3% (35%)	Inhibitory rTMS of the SMA for OCD and sequentially applied rTMS of SMA and DLPFC for OCD and comorbid depression was efficacious in a high proportion of patients. Responders had a mean improvement of 67% and non-responders had a mean improvement of 8%.
Lee et al., 2017 (Korea) [112]	9 (7;2)	OL pilot study	27 ± 4.8	✓	SMA	1200, 20 (5/week)	90–100%, 1 Hz, Figure 8 coil	16.8%	✕	✕	Inhibitory rTMS of the SMA led to marginal change in an open label pilot study.
Arumugham et al., 2018 (India) [113]	36 (28;8)	RCT, active or sham	A: 25 ± 5.3 S: 26 ± 6	✓	Pre-SMA	1200, 18 (6/week)	100%, 1 Hz, Figure 8 coil	A: 22.8% S: 16.1%	✕	A: 32% S: 18% (35%)	Inhibitory rTMS over pre-SMA was effective for a third of patients. Change in OC and anxiety symptoms were not statistically different between conditions.
Carmi et al., 2018 (Israel) [114]	41 (18;20)	RCT: LF, HF, or sham	LF: 25 ± 1.2 HF: 28 ± 0.7 S: 26 ± 1	✓	mPFC, ACC	LF: 900 HF: 2000 25 (5/week)	LF: 110%, 1 Hz, HF: 100%, 20 Hz Double cone coil (deep TMS)	HF: ~25% # S: ~5%	HF:~30% + S: ~7%	HF: 43.7% S: 7.14% (30%)	Inhibitory deep TMS of the mPFC and ACC during symptom provocation, led to inconsistent outcomes, and was omitted. The excitatory condition led to response in almost half of patients, change was statistically greater than sham, which emerged at 4 weeks and was maintained until 1 week FU.
Kumar et al., 2018 (India) [115]	25 (15;10)	Retrospective review	28.96 ± 4.0	✕	Left OFC	1200, 20 (5/week)	110%, 1 Hz, Figure 8 coil	24.1%*	25%	44% (35%)	Inhibitory rTMS of left OFC led to response in almost half of patients, mean change was maintained at 1 month FU.
Carmi et al., 2019 (US, Israel, Canada) [116]	94 (39;55)	Multi-site RCT, active or sham	A: 27.7 ± 3.87 S: 26.9 ± 4.13	✓	mPFC, ACC	2000, 29 (5/week)	100%, 20 Hz Double cone coil (deep TMS)	A: 21.6% # S: 11%	A: 23% S: 15.2%	A: 45.2% S: 11.8% (30%)	Excitatory deep TMS of mPFC and ACC combined with symptom provocation was effective for OC symptoms in almost half of patients and had a statistically significantly greater change than sham. Improvements were maintained at 4-week FU.
Harika-Germaneau et al., 2019 (France) [117]	28 (13;15)	RCT, active or sham	A: 30.1 ± 4.38 S: 29.4 ± 4.7	✓	Pre-SMA	600, 30 (5/week)	70%, 50 Hz (cTBS) Figure 8 coil	A: 13% S: 17%	✕	A: 28.5% S: 35.7% (25%)	Continuous TBS using neuronavigation over pre-SMA was effective for almost a third of patients; however the placebo effect was larger than the treatment effect. Improvements were sustained at 6-week FU (values not reported).
Singh et al., 2019 (India) [118]	79 (47;32)	Retrospective review	28.47 ± 5.57	✓	SMA (*n* = 46), Left OFC (*n* = 33)	1200, 20 (5/week)	110%, 1 Hz Figure 8 coil	27%	✕	40.5% full, 57% partial SMA: 39.1% OFC: 42.7% (35% full, 25% partial)	Inhibitory rTMS of SMA or OFC led to 27% change and 41% responders, which was almost comparable between the OFC and SMA targets.

A, active; dmPFC, dorsomedial prefrontal cortex; Hz, Herts; L, left; mPFC, medial prefrontal cortex; RMT, resting motor threshold; R, right; Rx, patients continued taking prescribed medication; S, sham; TAU, treatment as usual; + = clinically significant change from baseline; * = statistically significant change from baseline; # = statistically significant change compared to the control condition; ✓ = criterion applies; ✕ = not reported. ^ = Seo et al., 2016 [110] reported YBOCS score change not percentage change; ~ = outcomes were not reported and inferred from graphical reporting.

**Table 7:** Summary results of transcranial magnetic stimulation for OCD case studies.

Study (Country)	N (m:f)	Study Design	Illness Duration-Main Symptoms	Baseline YBOCS	Rx	Stimulation Parameters			YBOCS Outcomes % Change from Pre-Treatment		Comments/Conclusions
						Target	Pulses, sessions, frequency	RMT, frequency, coil	Post treatment	Follow up	
Talaei et al., 2009 (Iran) [119]	1 (0;1)	Case report	12y-contamination, religious, 20 previous ECT		✓	Vertex, right SMA	Vertex: ✕, 20 sessions Right SMA: 1200, 10 sessions	Vertex: 50%, 5 Hz Right SMA: 110%, 1 Hz ✕	Vertex: ✕ (deterioration) Right SMA: 76.3% +	✕	Excitatory rTMS of the vertex led to deterioration of OC symptoms (values not reported). The protocol was changed to inhibitory rTMS of the right SMA, which led to drastic improvement.
Mantovani et al., 2010 (USA) [120]	2 (2;0)	Case series	P1: 21y-contamination, aggression, symmetry P2: 23y-contamination, numbers.	P1: 26 P2: 30	✓	Pre-SMA	1800, 10 (5/week)	100%, 1 Hz, ✕	P1: 27% * + P2: 54% * +	✕	Inhibitory rTMS of pre-SMA using neuronavigation led to response in both patients. There was an average of 68% and 57% improvement in anxiety and depression symptoms, respectively.
Wu et al., 2010 (UK) [121]	1 (1;0)	Case report	16y-pathological doubt, MDD, previous suicidal episode	19	✕	cTBS: right DLPFCiTBS: left DLPFC	1200, 10 (cTBS: 2/week, 6 sessions; 1/ day, 4 sessions. iTBS: every second day)	80%, 50 Hz (TBS), Figure 8 coil	cTBS: 57.8% + iTBS: no effect	✕	Continuous TBS led to a drastic improvement in OCD symptoms by 58% and depressive symptoms by 40%. After 1 week, the intermittent TBS protocol was administered, OCD symptoms remained stable (increased by 1 point), and depressive symptoms further improved, to a total of 69% from baseline.
Winkelbeiner et al., 2018 (Switzerland) [122]	1 (1;0)	Case report	15y	✕	✓	Pre-SMA, left DLPFC	1200, 10 (5/week)	100%, 1 HZ, Figure 8 coil	Pre-SMA:✕ DLPFC: 25% +	✕	Inhibitory rTMS of the pre-SMA led to minimal improvement, stimulation was then applied to the left DLPFC, and led to response (values not reported).
Kar et al., 2019 (India) [123]	1 (1;0)	Case report	10y-contamination, aggression, blasphemy	31	✓	Pre-SMA	1200, 20 (6/week) m-rTMS: 1600, 24 (6/week)	100%, 1 Hz, ✕	35.4%+ m-rTMS: 57.6% +	✕	20 sessions of inhibitory rTMS of pre-SMA led to response, symptoms remained suppressed for 1 month, then deteriorated. 24 maintenance rTMS sessions led to an overall change of 65%. Efficacy was maintained at 3 month FU (values not reported).

cTBS, continuous theta burst stimulation; Hz, Herts; iTBS, intermittent theta burst stimulation; P, participant; RMT, resting motor threshold; Rx, patients continued taking prescribed medication; y, years; + = clinically significant change from baseline; * = statistically significant change from baseline; ✓ = criterion applies; ✕ = not reported.

**Table 8:** Summary results of transcranial magnetic stimulation for TS.

Study (Country)	Patient Demographics	Study Design	Baseline YGTSS	Rx	Stimulation Parameters			YGTSS Outcomes % Change from Pre-Treatment			Comments/Conclusions
**TS**	**N (m:f)**				Target	Dosage: pulses, sessions	Current: RMT, frequency, coil	Post treatment	Follow up	Responders (Criterion, if reported)	
Chae et al, 2004 (US) [124]	8 (5;3)	RCT pilot study of 2 targets, and 2 frequencies, cross over design	70 ± 22.4	✓	Motor cortex (M), PFC	2400, 20 (4/day)	110%, 1 Hz, 15 Hz	HF-PFC: 28.9% LF-PFC: 17.4% HF-M: 20.7% LF-M: 15.2% S: 22.4%	✕	0%	5 days of rTMS involving all conditions led to a mean improvement of 24% and no responders. Frequency and site of stimulation did not affect outcomes. There were many design limitations-sessions lasted up to 4 hours, each condition was tested for 1 day, no wash out, and patients were assessed directly after each session.
Orth et al., 2005 (UK) [125]	5 (✕)	RCT, cross over design: LR, L, sham	LR:46.2 ± 1 L: 51 ± 27.3 S: 48.2 ± 8	4/5	Pre-motor cortex	1800, 2 per protocol (1/day)	80% (AMT), 1 Hz, ✕	LR: 2.5% L: 0.3% (deterioration) S: 2.4% (deterioration)	✕	✕	2 sessions of inhibitory rTMS of the left, or bilateral pre motor cortex was ineffective for tic suppression.
Kwon et al., 2011 (South Korea) [126]	10 (10;0)	OL pilot study	20.6 ± 8.44	All	SMA	1200, 10 (5/week)	100% RMT, 1 Hz, Figure 8 coil	34.4% *	34.4%	✕	Inhibitory rTMS of SMA led to a moderate mean symptom improvement, which was maintained at 3 month follow up.
Le et al., 2013 (China) [127]	25 (22;3)	OL trial	22.9 ± 5.16	All	SMA	1200, 20 (5/week)	110%, 1 Hz, Figure 8 coil	31.4% *	30%	✕	Inhibitory rTMS of SMA led to a moderate mean symptom improvement, which was maintained 6 months following treatment.
Wu et al., 2014 (USA) [128]	12 (9;3)	RCT, active or sham	A: 27.5 ± 7.4 S: 26.8 ± 4.8	9/12	SMA	600 × 4, 2 (1/day)	90%, 30 Hz (cTBS) Figure 8 coil	✕	A: 15.2% S: 19%	A: 50% S: 50% (≥6 points)	4 continuous TBS trains per day delivered 15, 60 and 75 minutes apart led to minimal change at 1 week following treatment. The sham group had greater symptom change than active, yet quality of life improved by 44% in active and 4.3% in sham.
Landeros-Weisenberger et al., 2015 (USA) [129]	20 (16;4)	Phase 1: Multi-site RCT, active or sham Phase 2: OL	A: 35.8 ± 0.2 S: 36.3 ± 8.2	10/20	Anterior SMA	1800, 15 (5/week)	110%, 1 Hz, Figure 8	A: 17.3% S: 13.2% A-OL: 3.3% S-OL: 19.1% *	✕	A: 33% S: 18% A-OL: 71.4% S-OL: 44.4% (25%)	Inhibitory rTMS of anterior SMA led to moderate improvement from 15 sessions. 7 from active and 9 from sham received an additional 15 sessions within the OL phase. Outcome were largely favourable for those that received 30 sessions compared to 15 sessions.
Bloch et al., 2016 (Israel) [130]	12 (6;6)	OL pilot study	64.7 ± 23.1	10/12	SMA	1200, 20 (5/week)	110%, 1 Hz, Deep coil	4.6%	✕	✕	Inhibitory deep rTMS of the SMA was ineffective for TS. It was stated that those experiencing comorbid TS and OCD had a statistically significant reduction in both YBOCS and YGTSS, these outcomes were not reported.

A, active; AMT, active motor threshold; cTBS, continuous theta burst stimulation; Hz, Herts; L, left; RMT, resting motor threshold; R, right; Rx, patients continued taking prescribed medication; S, sham; * = statistically significant change from baseline; ✓ = criterion applies; ✕ = not reported.

**Table 9:** Summary results of transcranial magnetic stimulation for TS case studies.

Study (Country)	N (m:f)	Study Design	Illness Duration-Main Symptoms	Baseline YGTSS	Rx	Stimulation Parameters			YBOCS Outcomes % Change from Pre-Treatment		Comments/Conclusions
						Target	Pulses, sessions, frequency	RMT, frequency, coil	Post treatment	Follow up	
Mantovani et al., 2006 (Italy) [90]	4 (✕)	Case series	✕	71.2 ± 2.1	✓	SMA	1200, 10 (5/week)	100% RMT, 1 Hz, Figure 8 coil	67.1% +	✕	Inhibitory rTMS of SMA led to complete remission in 2 patients (YGTSS score of 70 to 0, and 90 to 0). Changes in depression and anxiety symptoms were not correlated with changes in YGTSS outcomes.
Mantovani et al., 2007 (USA) [131]	2 (2;0)	Case series	P12: SIB, OCD, ADHD, MDD P1: 14y P2: 10y	P1: 45 P2: 37	✓	SMA	1200, 10 (5/week)	110%, 1 Hz, Figure 8 coil	P1: 36% + P2: 68% +	P1:4% (deterioration) P2: 57%	Inhibitory rTMS of SMA led a partial response in P1, followed by a relapse after 1 month, 10 more sessions led to improvement of 36% again, which was maintained at 1 month FU. P2 had 68% change, which was maintained at 4-month FU.
Salatino et al., 2014 (Italy) [132]	1 (1;0)	Case report	35y-SIB, severe motor and vocal tics, OCD	85	✓	Pre-SMA	900 (day 1), 1200 (day 2), 2 (1/day)	80%, 1 Hz, Figure 8 coil	18%	✕	Two sessions of inhibitory rTMS of pre-SMA had marginal change. Yet a 75% improvement on the MOVES scale, and 23% deterioration in quality of life ocurred.

Hz, Herts; P, Participants; MOVES, motor tic, obsessions and compulsions, vocal tic evaluation survey; RMT, resting motor threshold; Rx, patients continued taking prescribed medication; SIB, self-injurious behavior; + = clinically significant change from baseline; ✓ = criterion applies; ✕ = not reported.

**Table 10:** Summary results of transcranial magnetic stimulation for skin picking.

Study (Country)	N (m:f)	Study Design	Baseline Ne-YBOCS	Rx	Stimulation Parameters			Ne-YBOCS Outcomes % Change from Pre-Treatment			Comments/Conclusions
					Target	Pulses, sessions, frequency	RMT, frequency, coil	Post treatment	Follow up	Responders (Criterion, if reported)	
Aydin et al., 2019 (Turkey) [133]	14 (2;12)	RCT, active or sham	A: 20.9 ± 3.3 S: 22.1 ± 5.3	7/14	Pre-SMA	1200, 15 (5/week)	100%, 1 Hz, Figure 8 coil	A: 35.9% + S: 15.8%	A: 70–110% (deterioration) S: ✕	A: 62.5% S: 33.3% (35%)	Inhibitory rTMS of pre-SMA was effective for a large proportion of skin picking patients immediately post treatment. However, at follow up, all patients had a drastic deterioration.

A, active; Hz, Herts; RMT, resting motor threshold; Rx, patients continued taking prescribed medication; S, sham; + = clinically significant change from baseline; ✕ = not reported.

**Table 11:** Summary results of transcranial magnetic stimulation for hoarding disorder.

Study (Country)	N (m:f)	Study Design	Baseline SI-R	Rx	Stimulation Parameters			SI-R Outcomes % Change from Pre-Treatment			COMMENTS/CONCLUSIONS
					Target	Pulses, sessions, frequency	RMT, frequency, coil	Post treatment	Follow up		
Diefenbach et al., 2015 (USA) [134]	1(0;1)	Case report	66	✕	Right DLPFC	900, 30 (5/week)	90%, 1 Hz, ✕	30.3%	31.8%		Inhibitory rTMS of the right DLPFC using neuronavigation was effective for hoarding disorder, and efficacy was maintained at 2-month FU.

A, active; Hz, Herts; RMT, resting motor threshold; Rx, patients continued taking prescribed medication; ✕ = not reported.

**Table 12:** Summary results of deep brain stimulation for obsessive compulsive disorder.

Study (Country)	N (m:f)	Study Design	Baseline YBOCS	Rx	Stimulation Parameters			YBOCS Outcomes % Change from Pre-Treatment			Comments/Conclusions
					Target (span of trajectory if reported)	Pulse width, Frequency	Stimulation intensity, and configuration (n)	Post treatment (≤6 months, or phase 1)	Follow up (>6 months, or phase 2)	Responders (Criterion, if reported)	
Gabriels et al., 2003 (Belgium) [152]	3 (1;2)	Case series	P1: 38 P2: 33 P3: 30	✓	ALIC	✗	9–10.5 V ✗	✗	12 months: P2: ~27% P3: ~46% + 32 months: P2: ~45% + P3: ~73% +	12 months: 33.3% 32 months: 66.6% (35%)	12 months of ALIC DBS led to response in one patient, partial response in another, and the other had DBS explanted. At 32 months of treatment, efficacy increased and 2 reached response.
Nuttin et al., 2003 (Belgium) [153]	4(✗)	Phase 1: RCT, cross over design Phase 2: OL trial	35 ± 4	✓	ALIC (E0 in NAc)	210/450 µs 100 Hz	4–10.5 V Multipolar (4) Bipolar (1)	A: 43.4% + S: 7.7%	21 months: 56% +	Phase 1: A: 75% S: 0% Phase 2: ✗ (35%)	3 months of closed label ALIC DBS achieved a mean improvement of 43%, and response in 3/4 patients compared to 7.7% improvement and no responders in sham. 2 patients reached phase 2 and improved by 56% at 21 month FU.
Greenberg et al., 2006 (USA) [154]	10(6;4)	OL trial	34.6 ± 0.6		VC/VS	90–210 µs 100–130 Hz	8–17 mA Monopolar (4) Bipolar (6) Unilateral (2)	27.7%	36 months: 35.5% +,*	50% full 75% partial (35% full, 25% partial)	3 months of ALIC DBS led to a mean improvement of 28% and 36 months led to 36% improvement. 20% achieved response at 6 months (2/10), and 50% (4/8) at 36 months.
Greenberg et al., 2010 (Belgium, USA) [155] Long-term FU of Gabriels (2003), Nuttin (2003) and Greenberg (2006) cohorts 26(14;12) Multi-site OL follow up			34 ± 0.5	✓	VC/VS (E0 in Nac)	✗ 100–130 Hz	≤10.5 V ✗	38.2% +,*	36 months: 38.5% +,*	1 month: 28% Last FU: 61.5% (35%)	3 months of VC/VS DBS led to mean improvement of 38%, and no further change at 36 months. 12 patients reached 36 month FU, all were included in the last FU (average 34 months), in which 62% reached response. Depression, anxiety and global functioning significantly improved by 53%, 50% and 69%, respectively, at last FU. CBT was resumed or initiated after 6–12 months. Outcomes of this cohort led to FDA and CE approval or ALIC DBS for TR-OCD.
Luyten et al., 2016 (Belgium, USA) [156] RCT and long-term follow up of Nuttin (2003), Gabriels (2003), Greenberg (2006; 2010) cohorts 24(12;12) Phase 1: Multi-site RCT, cross over design Phase 2: OL follow up			35 ^	✓	ALIC (6), BNST (15) ALIC + BNST (3)	90–450 µs 85–130 Hz	3–10.5 V Multipolar (5) Monopolar (4) Bipolar (8)	A: 42% #,*,+ S: 11% *	48 months: BNST: 50% + ALIC: 22% ALL: 66% +,*	Phase 1: A: 70% S: 26% Phase 2: BNST: 80% ALIC: 16.6% ALIC + BNST: 100% (35%)	3 months of closed label ALIC-BNST DBS (*n* = 17) led to 42% improvement compared to 11% in sham. 18 patients reached the 4-year FU, in which 66% improvement occurred. The optimised target shifted posterior with E0 in the BNST. BNST DBS led to an average of 50% improvement, compared to 22% from ALIC DBS, and 66% from both BNST and ALIC DBS. Anxiety, depression and global functioning improved by 45%, 49%, and 86%, respectively at last FU (54–171 months).
Abelson et al., 2005 (France) [157]	4 (2;2)	Phase 1: RCT, cross over design Phase 2: OL	32.75 ± 5.8	✓	ALIC (E0 in NAc)	60/210 μs 130/150 Hz	4–10.5 V Monopolar (1) Bipolar (3)	A: 19.8% S: 10.5%	Phase 2: 30.2%	Phase 1: A: 25% S: 0% Phase 2: 50% (35%)	Average improvement from two 3-week cycles of ALIC DBS was 20% compared to 11% from sham. The best outcome was reported in phase 2 (4–23 months), individually these were 0% (device explanted), 44% (committed suicide), 73%, and 4%. 2 reached response in phase 2.
Mallet et al., 2008 (France) [158]	16 (9;7)	Multi-site RCT, cross over design	On-off: 30–28 ^ Off-on: 28–31 ^	14/16	amSTN	60 µs 130 Hz	2.0 ± 0.8 V Monopolar (14) Bipolar (1) Mono- and bipolar (1) Unilateral (1)	A: 25.4% #,+ S: 4.1%	✗	A: 75% S: 37.5% (25%)	3 months of closed label amSTN DBS led to median improvement of 25% compared to 4% from sham. Global functioning (but not depression and anxiety) significantly improved in active compared to sham.
Mallet et al., 2019 (France) [13] Long-term FU of Mallet (2009) cohort 14 (6;8) OL follow up			32.4 ± 3.6	✗	amSTN	60 µs 130 Hz	1.2–4 V Monpolar (all)	✗	16 months: 35.4% + 48 months: 51.2% +	48 months: 75% full 92% partial (35% full, 25% partial)	16 and 48 months of amSTN led to mean improvement of 35% and 52%, respectively. Depression and anxiety improved by 53% and 61%, respectively at 4 years. 2 withdrew from the previous report.
Goodman et al., 2010 (USA) [159]	6 (2;4)	Phase 1: Pilot trial, staggered switch on (30 or 60 days post-op) Phase 2: OL	33.2 ± 2.1	✓	ALIC (E0 in VC/VS)	90–210 µs 130/135 Hz	2.5–8.5 V Monopolar (6)	Phase 1: ✗	12 months: 52.8% +,*	Phase 1: 50% Phase 2: 66.6% (35%)	2 or 3 months of ALIC DBS led to response in 3/6 patients (values not reported). At 12 months, mean improvement was 53%, which was not affected by staggered switch on. 2 remained as severe on the CGI, but requested DBS be maintained due to subjective relief of anxiety, depression and tic symptoms.
Fayad et al., 2016 (USA) [160] Long-term follow of Goodman (2010) cohort 6 (2;4) OL follow up			✗	5/6	VC/VS	150–210 µs 130/135 Hz	4–8.5 V Multipolar (2) Monopolar (1) Bipolar (1)	✗	✗	Last FU: 66.6% (35%)	6–9 years of VC/VS DBS led to response in the same 4 patients that achieved response from 12 months of treatment. 1 patient reached partial response of 26% improvement, and the other patient had the device switched off.
Huff et al., 2010 (Germany) [161]	10 (6;4)	Phase 1: RCT, cross over design Phase 2: OL	32.2 ± 4	✓	NAc (E2,3 in ALIC)	90 µs 145 Hz	4.5 V Multipolar (all)	A: 13.3% * S: 3.4%	6 months: 21.1%*	12 months: 10% full 50% partial (35% full, 25% partial)	3 months of closed label, unilateral NAc DBS led to mean improvement of 13.3% compared to 3.4% from sham. Following 3 and 6 months of open label DBS, improvements were 12.4% and 21.1%, respectively. At 12 month FU, 1 patient reached full response.
Mantione et al., 2014 (Netherlands) [162]	16 (9;7)	Phase 1: OL trial, then CBT added Phase 2: RCT, cross over design	33.7 ± 3.6	12/16	NAc (E3 in ALIC)	90 μs 130 Hz	Up to 5 V ✗	Phase 1: 24.6% * Phase 1, CBT: 46% +,*	Phase 2: A: 1.9% (deterioration) S: 44.9% (deterioration) 21 months: 52% +	Phase 1: 37.5% Phase 1, CBT: 56% (35%)	8 months of open label NAc DBS led to 25% improvement. A subsequent 24-week cycle with adjunct CBT led to a further significant improvement, reaching 46% change from pre-op, yet no significant change in depression or anxiety. The subsequent 4 week closed label phase (with CBT) led to deterioration of 1.9% from active and 44.9% from and sham. At 21 months post-op, mean improvement for OCD, anxiety and depression scores were 52%, 57%, and 46%, respectively.
Islam et al., 2015 (Italy) [163]	8 (7;1)	OL trial of 2 targets	Nac: 34.6 ± 4.1 BNST: 35.8 ± 2.2	✗	NAc (3) BNST (5)	90/210 µs 130/180 Hz	4.5–5.5 V Monopolar (4) Bipoar (4)	✗	6 months: Nac: 11.6% BNST: 38.5% +	✗	6 months of BNST DBS led to individual improvements of 25%, 10%, 0% in 3 patients, and NAc DBS led to improvements of 27.5%, 55%, 56%, 25%, 29% in 5 patients. Responders are reported from the last FU (6 months–5 years); 1 NAc patient had the device switched off, the other 2 reached 75% and 60% change at 5 years, 1 BNST patient was reported at 5 years with 30% change, the other 4 reached 6 month FU.
Farrand et al., 2018 (Australia) [164]	7 (3;4)	OL trial	32.4 ± 3.8	✓	NAc (3) BNST (3) NAc-left, BNST-right (1)	✗	✗ Monopolar (all)	✗	Last FU: BNST: 24.4% NAc: 23.4% BNST/NAc: 47.1% + All: 27.3% *	Last FU: BNST: 33.3% NAc: 33% BNST/NAc: 100% ALL: 42.8% (35% full)	Long-term (8–54 months) DBS of the BNST, NAc or both led to an average improvement of 24%, 23%, 47%, respectively. Individual change varied between 7–47%. Depression improved by 23% and anxiety deteriorated by 54% on average.
Barcia et al., 2019 (Spain) [165]	7 (3;4)	RCT, cross over design	32.2 ± 5	✓	NAc (E2-3 in caudate)	60 µs 130 Hz	4.5 V ✗	A: 51.3% +,* S: 25% *	✗	A: 85% S: ✗ (35%)	3 months of closed label NAc DBS with the optimal contact, achieved mean improvement of 52% compared to 25% from sham. The non-responder had a partial response of 25% improvement. 1 patient reached 93% improvement after 3 months (YBOCS = 1). Anxiety did not significantly change from any contact.
Lee et al., 2019 (USA) [166]	5 (2;3)	OL pilot study	35 ± 1.9	✓	ITP	90 µs 130 Hz	5–8.5 V Monopolar (all)	✗	12 months: 52% +,* Last FU: 54% +,*	12 months: 100% (35%)	1 year of ITP DBS led to 52% improvement in OC symptoms and response in all 5 patients, and 54% improvement at last FU (duration was not specified). Anxiety symptoms had a significant improvement at 2 year FU (but not 1 year).
Huys et al., 2019 (Germany) [167]	20 (10;10)	OL trial	30.9 ^	✗	NAc (E0,1), ALIC (E2,3)	90–210 µs 120–180 Hz	3–6 V Multipolar (all)	11.5% *	12 months: 33.3% *	12 months: 40% full 70% partial (35% full, 25% partial)	6 and 12 months of NAc-ALIC DBS led to median improvement of 12% and 33%, respectively. A further significant improvement at 6 and 12 months occurred. Anxiety and depressive symptoms did not significantly improve, and no predictors of response were identified.
Tyagi et al., 2019 (UK) [168]	6 (5;1)	Phase 1: RCT, cross over design of 2 targets Phase 2: OL trial; amSTN, VC/VS amSTN + VC/VS DBS (COMB), optimised settings (OPT), OPT + CBT	36.17 ± 0.75	✓	VC/VS (NAc-ALIC) + amSTN	60 µs 130 Hz	amSTN:1.4–2.6 V VC/VS: 5.4–7 V Monopolar (all)	Phase 1: amSTN: 45.2% +,* VC/VS: 52.9% +,*	Phase 2: COMB: 60.1% +,* OPT: 60.3% +,* OPT + CBT: 74.2% +,*	amSTN: 50% VC/VS: 83.3% COMB: 83.3% OPT: 100% OPT + CBT: 100% (35%)	3 months of closed label amSTN and VC/VS DBS led to mean improvement of 45% and 53%, respectively. There was no statistical effect of conditions (amSTN vs. VC/VS, single vs. both targets, COMB vs. OPT + CBT) on OC symptoms, however the optimised stimulation condition, and adjunct CBT had clinical superiority. Depressive symptoms significantly improved from VC/VS DBS and set shifting significantly improved from amSTN DBS.

A, active; CGI, clinical global impression; E, electrode; Hz, Hertz; P, participant; pre-op, pre-operative; SIB, self-injurious behavior; S, sham; V, Volts; µs, microsecond. + = clinically significant change from baseline; * = statistically significant change from baseline; # = statistically significant change compared to control condition; ✓ = criterion applies; ✗ = not reported; ~ = outcomes were not reported and inferred from graphical reporting. ^ Mallet et al., 2008 [158], Luyten et al., 2016 [156], and Huys et al., 2019 [167], reported median scores rather than mean scores.

**Table 13:** Summary results of deep brain stimulation for obsessive compulsive disorder case studies.

Study (Country)	N (m:f)	Study Design	Illness Duration-Main Symptom	Baseline YBOCS	Rx	Stimulation Parameters			YBOCS Outcomes % Change from Pre-Treatment		Comments/Conclusions
						Target (span of trajectory if reported)	Pulse width, Frequency	Stimulation intensity, and configuration (n)	Post treatment(≤6 months, or phase 1)	Follow up (6 months, or phase 2)	
Burdick et al., 2010 (USA) [169]	1 (1;0)	Case study	24y-mild TS	31	✕	NAc (to ALIC)	90 μs 135 Hz	3.5–6.5 V Bipolar	6 months: 6.4%	12 months: 12.9% (deterioration) 30 months: 0%	6 months of NAc DBS for OCD led to 6.4% improvement, however at 12 months the patient declined beyond baseline level, and at 30 months outcomes were comparable to baseline. Tic outcomes also showed the same pattern, with an average of 15% deterioration.
.Franzini et al., 2010 (Italy) [170]	2 (2;0)	Case series	P1:17y-house imagery, bipolar P1: 26y-BDD, MDD, phobic anxiety disorder	P1: 38 P2: 30	P1	NAc	90 μs 130 Hz	5–5.5 V Multipolar	✕	24 months: P1: 42.1% + P2: 33.3%	2 years of NAc DBS led to 42% and 33% improvement. Depression and GAF outcomes had a mean improvement of 58% and 50%, respectively.
Grant et al., 2011 (USA) [171]	1 (1;0)	Case study	5y-contamination	32	✓	NAc	✕	✕	✕	8 months: 68.7% +	8 months of NAc DBS led to 69% improvement.
Roh et al., 2012 (South Korea) [172]	4 (1;3)	Case series		37 ± 1.8	✓	VC/VS (E0 in NAc)	90–270 µs 90–130 Hz	2–5 V Bipolar (all)	✕	2 years: 60% * +	2 years of VC/VS DBS led to mean improvement of 60%, all 4 reached response, and 1 reached remission. Depressive symptoms improved more rapidly than OCD symptoms, reaching 42% change at 3 months and 66% at 2 years. The youngest patient showed more fluctuations in the pattern of response. CBT was resumed or initiated after 3 months.
Coenen et al., 2014 (Germany) [173]	2 (2;0)	Case series	P1: 19y-contamination, avoidance P2: 32y-violence, avoidance	P1: 39 P2: 30	✓	slMFB	60 µs 130 Hz	2.5–3.6 mA Bipolar (all)	✕	12 months: P1: 33.3% P2: 50% +	12 months of slMFB DBS led to partial and full response in 2 patients. P1 stabilized over months and reengaged in hobbies. P2 had improvements within hours and resumed their former occupation.
Tsai et al., 2014 (China) [174]	4 (4;0)	Case series	P1: 9y-contamination P2:11y-sexual P3: 5y-erotic images, SIB P4: 4y-contamination, spitting	P1: 36 P2: 36 P3: 34 P4: 39	✕	VC/VS	210 µs 130 Hz	✕ Monopolar (all)	✕	15 months: 33% *	15 months of VC/VS DBS led to a mean improvement of 33% in symptom severity, anxiety and depression.
Maarouf et al., 2016 (Germany) [175]	4 (1;3)	Retrospective case series	P1: 38y-contamination, ordering, BPD, BN P2: 19y-contamination, blasphemous P3: 17y-contamination, PTSD, BPD P4: 20y-numbers, colours	P1: 35 P2: 37 P3: 32 P4: 35	✓	Thalamus (medial dorsal and ventral anterior nucleus)	90 µs 130 Hz	0.5–4.5 V Multipolar (all)	✕	P1: 11.4% (3 months) P2: ✕ P3: 0% (7.5 months) P4: 17% (13 months)	P1 and P2 were initially implanted in the NAc, leads were then repositioned within the thalamus. P1, P2, and P3 had their device explanted, P3 developed new symptoms. P4 reported deterioration in mood and achieved partial response of 31% improvement only after 3 years. Depression worsened in the group that had previous DBS (P1,P2) and improved in the other patients, anxiety showed a similar pattern.
Chang et al., 2017 (China) [176]	1 (1;0)	Case study	8y-contamination, MDD	36	✓	VC/VS	210 μs 130 Hz	2–4 V Monopolar	✕	1 year: 30.5% 8 months after re-implant: 11%	1 year of VC/VS DBS led to 31% improvement, however the patient experienced compulsive skin picking and infection, the IPG was explanted and re-implanted 4 months later. 8 months after the re-implant improvement from initial pre-op was 11%.
Choudhury et al., 2017 (USA) [177]	1 (0;1)	Case study	21y-checking, hoarding	37	✓	ALIC (E0 in NAc)	210 μs 100 Hz	2–4 V Bipolar	4 months: 51.3%	1 year: 18.9%	~4 months of ALIC DBS led to 51% improvement, however the patient then experienced a gradual decline, at 1 year improvement was 19% from pre-op.
Gupta et al., 2019 (India)[178]	2 (0;2)	Case series	P1: 20y-contamination, reassurance P2: 26y-contamination, checking	P1: 38 P2: 38	✕	ALIC	✕	✕	3 months: P1: 63.1% P2: 68.4%	6/12 months: P1: 68.4% + P2: 78.7% +	3 months of ALIC (ventral, posterior) DBS led to 63% and 68% improvement. 6 and 12-month outcomes were comparable, with slightly further improvements.

A, active; BN, bulimia nervosa; BPD, borderline personality disorder; E, electrode; Hz, Hertz; IPG, implantable pulse generator; P, participant; pre-op, pre-operative; SIB, self-injurious behavior; S, Sham; V, Volts; y, years; µs, microsecond. + =clinically significant change from baseline; * =statistically significant change from baseline; ✓ = criterion applies; ✕ = not reported.

**Table 14:** Summary results of deep brain stimulation for Tourette’s.

Study (Country)	N (m;f)	Study Design	Baseline YGTSS	Rx	Stimulation Parameters			YBOCS Outcomes % Change from Pre-Treatment			Comments/Conclusions
**TS**					Target (span of trajectory if reported)	Pulse width, Frequency	Stimulation intensity, and configuration (n)	Post treatment (≤6 months, or phase 1)	Follow up (6 months, or phase 2)	Responders (Criterion, if reported)	
Maciunas et al., 2007 (USA) [179]	5 (5;0)	Phase 1: RCT Phase 2: OL trial	89 ± 9	✓	CM-Pf thalamus	60 µs 130 Hz	3.5–6.5 V Multipolar (3) Monopolar (3) Bipolar (4)	Phase 2: 43.5% +	✕	Phase 2: 60%	3 months of CM-Pf DBS led to 44% improvement and response in 3/5, the response criterion was not reported. RCT outcomes were not reported here, due to limited treatment duration per condition (7 days).
Servello et al., 2008 (UK) [180]	18 (15;3)	OL trial	80.8 ± 11.9	✓	CM-Pf-VOA	60–120 µs 130 Hz	2.5–4 V Bipolar (all)	✕	Last FU:64.6% + *	✕	CM-Pfc-VOA DBS led to 65% improvement at last FU (3–17 months).
Porta et al., 2009 (UK) [181] Long term follow up of Servello (2008) cohort 15 (12;3) OL follow up trial			76.5 ± 15.1	✕	CM-Pf-VOA	✕	✕	✕	24 months: 52.1% + *	✕	2 years of CM-Pfc-VOA led to 52% improvement. 3 discontinued DBS and 1 had additional leads implanted in the GPi.
Porta et al., 2012 (Italy) [182] Long-term follow up of Servello (2008) and Porta (2009) cohorts. 18(15;3) OL follow up trial			80.83 ± 11.9	11/ 18	CM-Pf-VOA	✕	✕	✕	Last FU: 72.6% + *	✕	5–6 years of CM-Pfc-VOA DBS led to 73% improvements. OC, anxiety and depression symptoms significantly improved by 42%, 46%, 55%, respectively. 2 had the device switched off, 1 deceased.
Ackermans et al., 2011 (Netherlands) [183]	6 (6;0)	Phase 1: RCT, cross over design Phase 2: OL trial	42.3 ± 3.1	5/6	CM-SP-VOA	60–210 μs 70–130 Hz	1–7.3 V Monopolar (3) Bipolar (3)	A: 39.5% #+ S: 2.8%	12 months: 49% + *	A: 66.6% S: 0% 1 year: 100% (33%)	3 months of closed label CM-SP-VOA DBS led to 40% improvement and response in 4/6 compared to 3% improvement and no responders from sham. At 1 year, OC symptoms improved by 49%.
Cannon et al., 2012 (Australia) [184]	11 (8;3)	OL trial	84.45 ± 13.6	✕	amGPi	60–120 μs 100–160 Hz	3–5 V Monopolar (all)	1 month: 46.7% * + 3 months: 49.6% + *	✕	3 months: 54.5% (50%)	1 and 3 months of amGPi DBS led to 47% and 50% improvement, respectively. Depression scores improved by 74%. Just 2 patients required tic medication after DBS.
Sachdev et al., 2014 (Australia) [185] Long term follow up of Cannon (2012) cohort with 6 additional patients 17 (14;3) OL follow up trial			81.2 ± 12.3	✕	amGPi	60–120 µs 110–160 Hz	3–5 V, ✕	1 month: 43.5% 3 months: 49.13%	Last FU: 54.3% + *	Last FU: 70.5% (50%)	amGPi DBS led 54% symptom improvement and response in 71% of patients at last FU (8–46 months), majority of improvements were achieved within 1 month, with no significant change after. 1 discontinued DBS at 3 months due to worsening of tics.
Motlagh et al., 2013 (USA) [186]	8 (8;0)	Clinical care study of 2 targets; midline thalamic nuclei (T), pv-GPi	40.2 ± 7.1	5/6	T (4) pv-GPi (2) T + pv-GPi (2)	T:60–210 µs 120–185 Hz pv-GPi: 90–180 µs130–185 Hz	T: 1.9–3.2 V GPi: 1–3 V Multipolar (1) Monopolar (1) Bipolar (3) Monopolar and bipolar (1) NR (2)	✕	Last FU: T: 45% GPi: 32% T + GPi: 36% All: 45%	All: 37.5% (50%)	Stimulation of the midline thalamic nuclei, pv-GPi, or both led to mean improvement of 45%, 32% and 36%, respectively at last FU (6–95 months). Individual change varied from 0–85%. The 3 responders were implanted in the midline thalamic nuclei.
Okun et al, 2013 (USA) [187]	5 (2;3)	OL Pilot study, staggered switch on (30 or 60 days post-op) of scheduled DBS	91.6 ± 8.8	✓	CM thalamus	160–400 µs 125 Hz	1–4 V Monopolar (4) Bipolar (1)	6 months: 19.4%*	✕	6 months: 0% (50%)	6 months of CM thalamus scheduled cycling DBS did not lead to response in any of the 5 patients and average improvement was 19%. Scheduled cycles varied from (seconds on/ seconds off): 2/10, 10/10, 16/80, 16/80. Staggered switch on did not affect 6-month outcomes.
Rossi et al., 2016 (USA) [188] Follow up of Okun et al., 2013 cohort 5 (2;3) OL follow up study			92.2 ± 9.3	✓	CM thalamus	80–320 µs 125 Hz	1–4.5 mA ✕	✕	Last FU: 29.6%	Last FU: 40% (40%)	CM thalamus scheduled cycling DBS led to a mean improvement of 30% (10–58%) and 2/5 reached response at last FU (24 months, and 18 months for 1 patient). P1-5 had the following total daily stimulation periods: 2.1 hours, 3.9 hours, 1.1 hours, 4 hours, 1.8 hours. Pulse trains also varied between patients.
Zhang et al., 2014 (China) [189]	13 (12:1)	Retrospective review	60.9 ± 15.1	6/13	pl-GPi	≤120 μs ≤185 Hz	≤3.6 V ✕	13.6% *	36 months: 55% + *	69.2% (40%)	6 months of pl-GPi DBS led to 14% improvement at 6 months, which improved every 6 months to reach 55% at 36 months. 1 patient was explanted at 1 week.
Kefalopoulou et al., 2015 (UK) [190]	15 (11;4)	Phase 1: Multi-site RCT, cross-over Phase 2: OL trial	87.9 ± 9.2	11/14	Am-GPi (13) pv-GPi (2)	60/90 μs 125–180 Hz	1–4 V Monopolar (11) Multipolar (4)	A:22.2% # S: 8.1%	Last FU: 41.4% +	Last FU: 60% (40%)	2 withdrew prior to switch on, outcomes for 13 are reported here. 3 months of closed label GPi DBS led to 22% mean improvement compared 8% from sham. At last FU (8–36 months) 41% improvement was achieved. 2 had comorbid dystonia and were implanted in the pv-GPi. Depression and quality of life (not OC and anxiety) outcomes significantly improved at last FU.
Huys et al., 2016 (Germany) [191]	8 (5;3)	OL trial	71.75 ✕	✕	Thalamus (ventral anterior and ventrolateral)	60–150 µs 80–130 Hz	1.3–3.7 V Multipolar (4) Monopolar (3) Bipolar (1) Unilateral (2)	6 months: 55.4% + *	12 months: 55.7% + *	✕	6 months of thalamic DBS led to 55% improvements, which was maintained at 12 months. Lower baseline scores on compulsivity, anxiety, emotional dysregulation, and inhibition were associated with greater outcomes. The patient with the lowest pre-op severity (YGTSS = 46) achieved complete remission (YGTSS = 0) at 1 year.
Testini et al., 2016 (USA) [192]	11(8;3)	Retrospective review	85 ± 8.6	✕	Thalamus (CM-Pfc)	✕	≥4V	✕	Last FU: 54% + *	62.6% (40%)	CM-Pfc DBS led to 54% symptom improvement and 63% response at last FU (2–91 months, mean 26 months).
Welter et al., 2017 (France) [193]	16 (12;4)	Phase 1: Multisite RCT, active or sham Phase 2: OL trial	75.3 ± 10.3 ^	14/16	aGPi	60–150 µs 130 Hz	2.5–4 V Multipolar (4) Monopolar (12)	A: 10.2% S: 4.2% (deterioration)	Phase 2: 40.2% +	A: 28.5% S:22.2% Phase 2: 87.5% (25%)	3 withdrew prior to the closed label period, outcomes for 13 patients are reported. 3 months of closed label aGPi DBS led to 10.2% improvement compared to 4.2% deterioration from sham. After 6 months of open label treatment improvements reached 40%. 2 out of 4 non-responders had active contacts outside the aGPi.
Welter et al., 2019 (France) [194] Follow up of Welter (2017) cohort with 3 additional patients 16(12;4) OL follow up			75.4 ± 11.1 ^	✓	a-GPi	✕	✕	✕	12 months: 40.1% + 30 months: 48.1% +	75%	12 months and 30 months of aGPi DBS led to mean improvement of 40.1% and 48%, respectively. 7/12 patients were considered responders; however the criterion was not stated. Responders improved by 70% on average, where-as non-responders deteriorated by 1–19%.
Azimi et al., 2018 (Iran) [195]	6 (4;2)	OL trial	75.6 ± 16.5	✓	Am-GPi	60/90 µs 110–155 Hz	2.5–6 V Multipolar (all)	✕	12 months: 62.5% + *	✕	12 months of amGPi DBS led to an average improvement of 63%. Quality of life improved by 162%.
Brito et al., 2019 (Brazil) [196]	5 (5;0)	Retrospective review	82 ± 9	4/5	CM-Pfc Thalamus	✕	✕	✕	12 months: 29.7%	60% (40%)	12 months of CM-Pf DBS led to 30% improvement and response in 3/5 patients, no other clinical outcomes were reported.

A, active; E, electrode; Hz, Hertz; S, Sham; V, Volts; µs, microsecond; + = clinically significant change from baseline; * = statistically significant change from baseline; # = statistically significant change compared to control condition; ✓ = criterion applies; ✕ = not reported. ^ Welter et al., 2019 [194] reported median scores not mean. ^Welter et al., 2017 [193] reported change from post-operative/ pre-switch on as baseline.

**Table 15:** Summary results of deep brain stimulation for Tourette’s case studies.

Study (Country)	N (m:f)	Study Design	Illness Duration-Main Symptom,	Baseline YGTSS	Rx	Stimulation Parameters			YBOCS Outcomes % Change from Pre-Treatment		Comments/Conclusions
**Case studies-TS**						Target (span of trajectory if reported)	Pulse width, Frequency	Stimulation intensity, and configuration (n)	Post treatment (≤6 months, or phase 1)	Follow up (6 months, or phase 2)	
Diederich et al., 2005 (Australia) [197]	1 (1;0)	Case study	7y-complex motor and vocal tics	83	No	pv-GPi	60 µs 185 Hz	2 V Bipolar	✕	14 months: 46.9% +	14 months of GPi DBS led to 47% improvement.
Flaherty et al., 2005 (USA) [198]	1 (0;1)	Case study	17y-frequent vocal tics, head/arm jerks	✕	✕	ALIC (to NAc)	210 μs 185 Hz	4.1 V Bipolar	✕	18 months: 25%	18 months of ALIC DBS led to 25% improvement, and subjective tic frequency improved by 45%. It was reported that nearly all clinical effects were apparent within a few days of programming adjustments.
Houeto et al., 2005 (France) [199]	1 (1;0)	Randomised controlled case study	29y-SIB, previous ECT, frequent shrieking	84	✓	CM-Pfc Thalamus, amGPi	60 μs 130 Hz	1.5 V Thalamus: multipolar GPi: monopolar	CM-Pf: 61.5% + amGPi: 58.5% + S: 213.7% (deterioration) CM-Pf + amGPi: 62.6% +	✕	2 months of CM-Pf DBS led to 61% improvement, a consecutive phase of amGPi DBS led to 3% deterioration, a subsequent sham phase led to a 214% deterioration, CM-Pf and amGPi DBS then led 63% improvement. CM-Pf DBS led to disappearance of SIB, and GPi DBS led to worsened mood and impulsivity, sham led to panic attacks.
Kuhn et al., 2007 (Germany) [200]	1 (1;0)	Case study	~14y-autoaggressive, automutilation, spitting, coprolalia, SIB, OCD.	90	✕	NAc	90 µs 130 Hz	7 V Multipolar	✕	30 months: 41% +	30 months of NAc DBS led to 41% improvement and remission in 1 patient. OC symptoms and global functioning improved by 52% and 485%, respectively. All 4 contacts were active for chronic settings.
Shahed et al., 2007 (USA) [201]	1 (1;0)	Case study	13y-coprolalia, copropraxia, SIB, anxiety, ADHD, OCD.	90	✓	p-GPi	90 μs 145–160 Hz	5 V Monopolar	6 months: 85% +	✕	6 months of GPi DBS led to 85% improvement. Obsessions, compulsions, anxiety and depression outcomes improved by 100%, 29%, 55%, 66%, respectively.
Dehning et al., 2008 (Germany) [202]	1 (0;1)	Case study	24y-self-mutilation, contamination, 2 suicide attempts, previous ECT	83	✓	GPi	120–210 μs 130–145 Hz	4.2 V Monopolar	6 weeks: 66.2% +	12 months: 87.9% +	6 weeks of GPi DBS led to 66% improvement, and 88% at 12 months. The patient was in full remission.
Shields et al., 2008 (USA) [203]	1 (0;1)	Case study	30y-vocal tics, head/arm jerks (led to limb fracture and blindness)	79	✓	ALIC, CM thalamus	210 µs 185 Hz	ALIC: 4.1 V Thalamus: 7 V	ALIC: 22.7% Thalamus: 45.5% +	✕	18 months of ALIC DBS led to 23% improvement, yet due to lead damage from jerking and mood side effects leads were re-implanted in the CM thalamus. 3 months of CM thalamus DBS led to 46% improvement (from original pre-op score).
Welter et al., 2008 (France) [204]	3 (1;2)	Phase 1: RCT, cross over design, Phase 2: OL trial	P1: 19y-copropraxia, coprolalia, SIB, BPD P2: 24y-jerks, shouting, SIB, arithmomania P3: 17y-jerks, motor and vocal tics.	✕	✓	CM-Pfc thalamus, GPi	60 µs 130 Hz	✕ ✕	CM-Pf: 44.6% + GPi: 78.3% + CM-Pf + GPi: 59.6% + S: ✕	P1: 82% + P3: 74% +	2 months of stimulation of CM-Pf thalamus, GPi, or both DBS, led to 45%, 78%, 60% improvement, respectively. Sham outcomes were not reported. Long term FU was reported for 2 patients, 82% (60 months) and 74% (20 months) improvements were achieved. All 3 patients had chronic GPi DBS, and 2 had combined GPi and CM-Pf thalamic DBS.
Dehning et al., 2011 (Germany) [45]	4 (1;3)	Case series	P1: SIB, vocal tics P2: motor and vocal tics P3: complex motor tics, head jerking P4: complex motor and extensive vocal tics, SIB	P1: 69 P2: 75 P3: 87 P4: 89	✕	pv-GPi	150–210 μs 130/145 Hz	3.5–4.2 V ✕	P2: 17% P3: 6% (deterioration)	P1: 88% + P4: 64% +	Individual symptom improvements from pv-GPi DBS were 88% (12 months), 17% (5 months), 64% (12 months); P3 had repositioning and the device switched off at 5 months. Responders (but not non-responders) had predominant SIB, and previous response to ECT.
Martinez-Fernandez et al., 2011 (USA) [205]	5 (4;1)	Case series	P1: coprophenomena, SIB P2: mild OCB, violent neck tics P3: 14y-violent neck tics, P4: coprophenomena, echophenomena, SIB	P1: 93 P2: 63 P3: 94 P4: 94	✓	P1: pv-GPi P2: am-GPi P3: am-GPi P4: am-GPi	60–210 μs 20–170 Hz	2.5–4 V Multipolar (3) Multipolar and monopolar (1) Bipolar (1)	✕	Last FU: P1: 10.7% P2: 19% P3: 31.9% P4: 62.7% +	1 patient was treated for dystonia, the remaining 4 are reported here. 6 months of GPi DBS led to mean improvement of 20%, which reached 29% at last FU (9–24 months). P2 was initially implanted with pv-GPi leads and underwent repositioning within the am-GPi at 18 months due to worsening of tics.
Pullen et al., 2011 (USA) [206]	1 (1;0)	Case study	ADHD, OCD	77	✕	CM-Pfc thalamus	✕	✕	✕	8 months: 81.8% +	8 months of CM-Pf thalamus DBS led to 82% improvement. Anxiety and depression improved by 25% and 33%, respectively.
Rzesnitzek et al., 2011 (USA) [207]	1 (1;0)	Case study	Grunting, SIB, OCD, intrusive thoughts, 2 suicide attempts	77	✕	CM-Pfc thalamus	130 Hz 60 μs	6–8 V Monopolar	✕	24 months: 83.1% +	24 months of CM-Pf thalamus DBS led to 83% improvement. OC symptoms were in complete remission (YBOCS of 0).
Savica et al, 2012 (USA) [208]	3 (2;1)	Case series	P1: 12y-severe motor and vocal tics, MDD, OCD P2: 25y-severe vocal tic, OCD, ADHD P3: 9y-complex vocal tics, head jerks, OCD, ADHD	P1: 93 P2: 80 P3: 70	✕	CM-Pfc thalamus	90–120 μs 107–130 Hz	2.5–4.1 V Multipolar (P3) Bipolar (P1,2)	✕	12 months: P1: 68.8% + P2: 60% + P3: 80% +	12 months of CM-Pf thalamus DBS led to response in 3 patients with 69%, 60% and 80% improvements. These patients are included in the Testini (2016) cohort above.
Massano et al., 2013 (Portugal) [209]	1 (0;1)	Case study	Coprolalia, motor and vocal tics, unable to attend school, OCB	81	✓	Am-GPi	90 µs 130 Hz	3.2–3.5 V Monopolar	49.3% +	24 months: 60.5% +	3 months of amGPi DBS led to 49% improvement, there was a slight decline at 1 year (37% improvement from baseline) in which stimulation current was increased and led to further benefit within 3–4 days. Tic, OC, anxiety and depressive symptoms improved by 61%, 43%, 63% and 79%, respectively at 2 year FU.
Piedimonte et al., 2013 (Argentina) [210]	1 (1;0)	Case study	Motor and phonic tics, refused medication	78	No	GPe	300 µs 150 Hz	3 V Multipolar	3 months: 57.7% + 6 months: 70.5% +	✕	3 and 6 months of GPe DBS led to 58% and 71% improvement, respectively. At 2 years, in which the battery was depleted, the patient declined slightly to 38% improvement from baseline. Anxiety and depression improved by 75% and 82% at 6 months respectively, and anxiety (but not depression) declined on battery depletion.
Dong et al., 2014 (China) [211]	1 (1;0)	Case study	25y-copropalia, neck rotation, shoulder jerks, OCB, depression	56	✕	pv-GPi	90 μs 130 Hz 65 Hz (33 months)	2.8 V Multipolar	1 month: 50% + 3 months: 86% +	33 months: 92.9% +	1 and 3 months of pvGPi DBS led to 50% and 86% improvement, respectively. At 33 months low frequency DBS was applied and improvement reached 93%. OCB disappeared at 1 month, and depression disappeared at 3 months.
Huasen et al., 2014 (UK) [212]	1 (0;1)	Case study	~6y-motor and vocal tics, coprolalia, neck jerks, OCD	83	✕	am-GPi	180 μs 180 Hz	2.8–2.9 V Monopolar	✕	12 months: 67.4% +	12 months of amGPi DBS led to 67% improvement.
Nair et al., 2014 (Australia) [213]	4 (4;0)	Case series	P1: 16y-grunting, OCD P2: 23y-coprolalia, echolalia, ADHD, OCD P3: 33y-jerking, OCD P4: 9y-coprolalia, violent motor tics, OCD	P1: 86 P2: 96 P3: 84 P4: 99	✕	am-GPi	60–90 µs 120–160 Hz	2.3–4.4 mA Monopolar (all)	✕	P1: 94.1% + P2: 93.7% + P3: 90.4% + P4: 94.9% +	amGPi DBS was effective for all comorbid TS and OCD patients, with 90–95% improvements in tic symptoms and 85–100% improvements in OC symptoms. Assessments were conducted between 3–26 months post-op.
Patel et al., 2014 (USA) [214]	1 (0;1)	Case study	Motor and vocal tics, SIB, OCB, ADHD, depression, mild PD	89	✓	GPi	90/110 µs 150 Hz	5.5 V Monopolar	47% +	✕	6 months of GPi DBS led to 47% improvement.
Wojtecki et al., 2016 (Germany) [215]	1 (0;1)	Case study	15y-contamination, washing, depression	38	✓	am-STN	60 µs 130 Hz	2.5 V Monopolar	28.9%	36 months: 92% +	3 months of amSTN DBS led to 29% improvement, which reached 92% at 3 years. Depression outcomes improved by 85% at 3 months.
Kano et al., 2018 (Japan) [216]	2 (2;0)	Case series	P1: 8y-coprolalia P2: 17y-coprolalia, depression	P1: 84 P2: 83	✕	CM-Pfc Thalamus	180–330 µs 125/145 Hz	2.5–3.5 V Multipolar (P1) Multipolar and bipolar (P2)	P1: 50% + P2: 14.4%	P1: 48.8% + P2: 19.2%	CM-Pfc thalamic DBS led to individual improvements of 50% (4 months) and 14% (10 months) which reached 49% (35 months) and 19% (29 months) from long term treatment.
Kakusa et al., 2019 (USA) [217]	1 (1;0)	Case study	~10y-head and neck tics, OCD, ADHD, MDD, chronic pain, opioid use disorder	70	✕	CM-Pfc thalamus + VC/VS (ALIC-NAc)	90 µs 130 Hz	2.5–5 V Bipolar	60% +	84.2% +	4 months of CM-Pf and VC/VS DBS led to 60% improvement, and disappearance of vocal tics. OC symptom also improved by 70%. At 8 months, YGTSS improvement increased to 84%. A 1 year, depression improved by 95%. The active contact in the VC/VS lead was within the NAc.
Rossi et al., 2019 (Argentina) [218]	1 (0;1)	Case study	≥10y, severe phonic and motor tic	93	✕	GPi	130 µs 130 Hz	4 mA Monopolar	2 months: 67.7% + 6 months: 86% +	12 months: 87% +	2 months of GPi DBS led to 68% improvement, at 6 and 12 months, 86% and 87% improvements were reached, respectively. At 14 months unilateral lead failure was detected, YGTSS outcomes were unaffected. Depressive symptoms improved by 88% at 1 year.
Zhu et al., 2019 (China) [219]	4 (4;0)	Case series	✕	P1: 86 P2: 64 P3: 74 P4: ✕	✕	GPi lateral STN	60–70 µs, 135–145 Hz	2.35–3.3 V, Bipolar (all)	P1: 48.8% + P2: 45.3% + P3: 14.8% P4: ✕	✕	6 months of GPi and lateral STN DBS led to an average improvement of 41%. 2/4 patients reached response, P4 withdrew as efficacy did not meet expectations, outcomes were not reported.

A, active; BPD, bipolar personality disorder; E, electrode; Hz, Hertz; OCB, obsessive-compulsive behavior; P, participant; PD, Parkinson’s disease; SIB, self-injurious behavior; S, Sham; V, Volts; µs, microsecond; y, years; + = clinically significant change from baseline; ✓ = criterion applies; ✕ = not reported.

**Table 16:** Summary results of deep brain stimulation for body dysmorphic disorder.

Study (Country)	Patient Demographics	Study Design	Illness Duration-Main Symptom	Baseline BDD-YBOCS	Rx	Stimulation Parameters			YBOCS Outcomes % Change from Pre-Treatment		Comments/Conclusions %
	**N (m:f)**					Target (span of trajectory if reported)	Pulse width, Frequency	Stimulation intensity, and configuration (n)	Post treatment (≤6 months, or phase 1)	Follow up (6 months, or phase 2)	
Baldermann et al., 2016 (Germany) [22]	1 (1;0)	Case study	14y	39	✕	VC/VS	150 μs 150 Hz	2.4–3.2 V Multipolar	20.5%	3 months (after lead replacement): 36%	3 months of VC/VS DBS led to 21% improvement, when the active contact was shifted dorsally, improved increased to 36%.

Hz, Hertz; V, Volts; µs, microsecond; y, years; ✕ = not reported.

## Data Availability

Data is contained within the article (see summary tables), extracted data from articles can be made available upon request.

## Supplementary Materials

The following are available online at https://www.mdpi.com/2076-3425/11/7/948/s1, Supplementary results, S1 PRISMA diagram, S2 Risk of Bias Table, S3 Quality assessments table, S4 DBS targets.

## References

[1] (2013). Diagnostic and Statistical Manual of Mental Disorders: DSM-5.

[2] Como P.G., LaMarsh J., O’Brien K.A. (2005). Obsessive-Compulsive Disorder in Tourette’s Syndrome. Adv. Neurol..

[3] Zilhão N.R., Smit D.J., Boomsma D.I., Cath D.C. (2016). Cross-Disorder Genetic Analysis of Tic Disorders, Obsessive-Compulsive, and Hoarding Symptoms. Front. Psychiatry.

[4] Atmaca M. (2016). Treatment-refractory obsessive compulsive disorder. Prog. Neuro Psychopharmacol. Biol. Psychiatry.

[5] Denys D. (2006). Pharmacotherapy of obsessive-compulsive disorder and obsessive-compulsive spectrum disorders. Psychiatr Clin. North Am..

[6] Denys D., Mantione M., Figee M., van den Munckhof P., Koerselman F., Westenberg H., Bosch A., Schuurman R. (2010). Deep Brain Stimulation of the Nucleus Accumbens for Treatment-Refractory Obsessive-Compulsive Disorder. Arch. Gen. Psychiatry.

[7] Ring H.A., Serra-Mestres J. (2002). Neuropsychiatry of the basal ganglia. J. Neurol. Neurosurg. Psychiatry.

[8] Nakao T., Okada K., Kanba S. (2014). Neurobiological model of obsessive-compulsive disorder: Evidence from recent neuropsychological and neuroimaging findings. Psychiatry Clin. Neurosci..

[9] Dougherty D., Rauch S., Greenberg B.D. (2020). Pathophysiology of Obsessive-Compulsive and Related Disorders. The American Psychiatric Association Publishing Textbook of Anxiety, Trauma and OCD-Related Disorders.

[10] Millet B., Dondaine T., Reymann J.-M., Bourguignon A., Naudet F., Jaafari N., Drapier D., Turmel V., Mesbah H., Vérin M. (2013). Obsessive Compulsive Disorder Networks: Positron Emission Tomography and Neuropsychology Provide New Insights. PLoS ONE.

[11] Abramovitch A., Abramowitz J.S., Mittelman A. (2013). The neuropsychology of adult obsessive–compulsive disorder: A meta-analysis. Clin. Psychol. Rev..

[12] Abramowitz J.S., McKay D., Storch E.A. (2017). The Wiley Handbook of Obsessive Compulsive Disorders.

[13] Mallet L., Du Montcel S.T., Clair A.H., Arbus C., Bardinet E., Baup N., Chabardes S., Chereau I., Czernecki V., Fontaine D. (2019). Long-term effects of subthalamic stimulation in Obsessive-Compulsive Disorder: Follow-up of a randomized controlled trial. Brain Stimul..

[14] Feusner J.D., Moody T., Hembacher E., Townsend J., McKinley M., Moller H., Bookheimer S. (2010). Abnormalities of Visual Processing and Frontostriatal Systems in Body Dysmorphic Disorder. Arch. Gen. Psychiatry.

[15] Buchanan B., Rossell S., Maller J.J., Toh W.L., Brennan S., Castle D. (2014). Regional brain volumes in body dysmorphic disorder compared to controls. Aust. N. Z. J. Psychiatry.

[16] Beucke J.C., Sepulcre J., Buhlmann U., Kathmann N., Moody T., Feusner J.D. (2016). Degree connectivity in body dysmorphic disorder and relationships with obsessive and compulsive symptoms. Eur. Neuropsychopharmacol..

[17] Marsh R.P.D., Zhu H.P.D., Wang Z.P.D., Skudlarski P.P.D., Peterson B.S.M.D. (2007). A Developmental fMRI Study of Self-Regulatory Control in Tourette’s Syndrome. Am. J. Psychiatry.

[18] Wang Z., Maia T.V., Marsh R., Colibazzi T., Gerber A., Peterson B.S. (2011). The Neural Circuits That Generate Tics in Tourette’s Syndrome. Am. J. Psychiatry.

[19] Grant J.E., Stein D.J., Woods D.W., Keuthen N.J. (2012). Trichotillomania, Skin Picking, and Other Body-Focused Repetitive Behaviors.

[20] Kohl S., Schönherr D.M., Luigjes J., Denys D., Mueller U.J., Lenartz D., Visser-Vandewalle V., Kuhn J. (2014). Deep brain stimulation for treatment-refractory obsessive compulsive disorder: A systematic review. BMC Psychiatry.

[21] Moher D., Liberati A., Tetzlaff J., Altman D.G. (2009). Preferred Reporting Items for Systematic Reviews and Meta-Analyses: The PRISMA Statement. PLoS Med..

[22] Baldermann J.C., Kohl S., Visser-Vandewalle V., Klehr M., Huys D., Kuhn J. (2016). Deep Brain Stimulation of the Ventral Capsule/Ventral Striatum Reproducibly Improves Symptoms of Body Dysmorphic Disorder. Brain Stimul..

[23] Brunelin J., Mondino M., Bation R., Palm U., Saoud M., Poulet E. (2018). Transcranial Direct Current Stimulation for Obsessive-Compulsive Disorder: A Systematic Review. Brain Sci..

[24] Dos Santos-Ribeiro S., de Salles Andrade J.B., Quintas J.N., Baptista K.B., Moreira-de-Oliveira M.E., Yucel M., Fontenelle L.F. (2018). A Systematic Review of the Utility of Electroconvulsive Therapy in Broadly Defined Obsessive-Compulsive-Related Disorders. Prim. Care Companion CNS Disord..

[25] Eapen V., Baker R., Walter A., Raghupathy V., Wehrman J.J., Sowman P.F. (2017). The Role of Transcranial Direct Current Stimulation (tDCS) in Tourette Syndrome: A Review and Preliminary Findings. Brain Sci..

[26] Fontenelle L.F., Coutinho E.S., Lins-Martins N.M., Fitzgerald P.B., Fujiwara H., Yucel M. (2015). Electroconvulsive therapy for obsessive-compulsive disorder: A systematic review. J. Clin. Psychiatry.

[27] Hsu C.W., Wang L.J., Lin P.Y. (2018). Efficacy of repetitive transcranial magnetic stimulation for Tourette syndrome: A systematic review and meta-analysis. Brain Stimul..

[28] Lusicic A., Schruers K.R., Pallanti S., Castle D.J. (2018). Transcranial magnetic stimulation in the treatment of obsessive-compulsive disorder: Current perspectives. Neuropsychiatr. Dis. Treat..

[29] Rapinesi C., Kotzalidis G.D., Ferracuti S., Sani G., Girardi P., Del Casale A. (2019). Brain Stimulation in Obsessive-Compulsive Disorder (OCD): A Systematic Review. Curr. Neuropharmacol..

[30] Trevizol A.P., Shiozawa P., Cook I.A., Sato I.A., Kaku C.B., Guimaraes F.B., Sachdev P., Sarkhel S., Cordeiro Q. (2016). Transcranial Magnetic Stimulation for Obsessive-Compulsive Disorder: An Updated Systematic Review and Meta-analysis. J. ECT.

[31] Vázquez-Bourgon J., Martino J., Sierra Peña M., Infante Ceberio J., Martínez Martínez M.Á., Ocón R., Menchón J.M., Crespo Facorro B., Vázquez-Barquero A. (2019). Deep brain stimulation and treatment-resistant obsessive-compulsive disorder: A systematic review. Rev. Psiquiatr. Salud Ment. (Engl. Ed.).

[32] Vicheva P., Butler M., Shotbolt P. (2020). Deep brain stimulation for obsessive-compulsive disorder: A systematic review of randomised controlled trials. Neurosci. Biobehav. Rev..

[33] Higgins J.P., Altman D.G., Gotzsche P.C., Juni P., Moher D., Oxman A.D., Savovic J., Schulz K.F., Weeks L., Sterne J.A. (2011). The Cochrane Collaboration’s tool for assessing risk of bias in randomised trials. BMJ.

[34] Jadad A.R., Moore R.A., Carroll D., Jenkinson C., Reynolds D.J.M., Gavaghan D.J., McQuay H.J. (1996). Assessing the quality of reports of randomized clinical trials: Is blinding necessary?. Control. Clin. Trials.

[35] The GRADE Working Group (2013). Grading of Recommendations, Assessment, Development and Evaluation (GRADE) Handbook.

[36] Pallanti S., Hollander E., Bienstock C., Koran L., Leckman J., Marazziti D., Pato M., Stein D., Zohar J., International Treatment Refractory OCD Consortium (2002). Treatment non-response in OCD: Methodological issues and operational definitions. Int. J. Neuropsychopharmacol..

[37] Valadas M.T.T.R.T., De Bragança M.Â.M.F. (2017). Electroconvulsive therapy’s mechanisms of action: A review. Clin. Neuropsychiatry.

[38] Maletzky B., McFarland B., Burt A. (1994). Refractory obsessive compulsive disorder and ECT. Convuls. Ther..

[39] Tomruk N.B., Saatcioglu O., Ugurlu E., Hacioglu M. (2016). ECT Use in Refractory Obsessive-Compulsive Disorder. Klin. Psikofarmakol. Bülteni Bull. Clin. Psychopharmacol..

[40] Liu X., Cui H., Wei Q., Wang Y., Wang K., Wang C., Zhu C., Xie X. (2014). Electroconvulsive therapy on severe obsessive-compulsive disorder comorbid depressive symptoms. Psychiatry Investig..

[41] Manhas R.S., Mushtaq R., Tarfarosh S.F., Shoib S., Dar M.M., Hussain A., Shah T., Shah S., Manzoor M. (2016). An Interventional Study on the Clinical Usefulness and Outcomes of Electroconvulsive Therapy in Medication-Resistant Mental Disorders. Cureus.

[42] Agrawal A., Das S., Thirthalli J. (2018). When Obsessive Compulsive Disorder Responds Only to Electroconvulsive Therapy: A Rare Case for Maintenance Electroconvulsive Therapy?. J. Neurosci. Rural Pr..

[43] Aggarwal A., Aggarwal N., Prasad S. (2019). Electroconvulsive Therapy in Severe Obsessive-Compulsive Disorder. Prim. Care Companion CNS Disord..

[44] Morais S.L., Derenusson G.N., Pinto J.P., Hounie A.G., Dursun S.M., Wichert-Ana L., Kato M., de Oliveira L.F., de Azevedo-Marques P.M., Sakamoto A.C. (2007). Neurobiological Substrates of Electroconvulsive Therapy for Tourette Syndrome: A Serial SISCOM Study. J. ECT.

[45] Dehning S., Feddersen B., Mehrkens J.H., Muller N. (2011). Long-term results of electroconvulsive therapy in severe Gilles de la Tourette syndrome. J. ECT.

[46] Rajashree V.C., Manjiri C.D., Ivan S.N., Alka V.P. (2014). Gilles de la Tourette’s syndrome successfully treated with electroconvulsive therapy. Indian J. Psychiatry.

[47] Guo J.N., Kothari J.S., Leckman J.F., Ostroff R.B. (2016). Successful Treatment of Tourette Syndrome With Electroconvulsive Therapy: A Case Report. Biol. Psychiatry.

[48] Nitsche M.A., Paulus W. (2000). Excitability changes induced in the human motor cortex by weak transcranial direct current stimulation. J. Physiol..

[49] Stagg C.J., Nitsche M.A. (2011). Physiological basis of transcranial direct current stimulation. Neuroscientist.

[50] Keeser D., Meindl T., Bor J., Palm U., Pogarell O., Mulert C., Brunelin J., Möller H.-J., Reiser M., Padberg F. (2011). Prefrontal Transcranial Direct Current Stimulation Changes Connectivity of Resting-State Networks during fMRI. J. Neurosci..

[51] Bation R., Poulet E., Haesebaert F., Saoud M., Brunelin J. (2016). Transcranial direct current stimulation in treatment-resistant obsessive-compulsive disorder: An open-label pilot study. Prog. Neuropsychopharmacol. Biol. Psychiatry.

[52] Dinn W.M., Aycicegi-Dinn A., Göral F., Karamursel S., Yildirim E.A., Hacioglu-Yildirim M., Gansler D.A., Doruk D., Fregni F. (2016). Treatment-resistant obsessive-compulsive disorder: Insights from an open trial of transcranial direct current stimulation (tDCS) to design a RCT. Neurol. Psychiatry Brain Res..

[53] D’Urso G., Brunoni A.R., Mazzaferro M.P., Anastasia A., de Bartolomeis A., Mantovani A. (2016). Transcranial direct current stimulation for obsessive-compulsive disorder: A randomized, controlled, partial crossover trial. Depress. Anxiety.

[54] Klimke A., Nitsche M.A., Maurer K., Voss U. (2016). Case Report: Successful Treatment of Therapy-Resistant OCD with Application of Transcranial Alternating Current Stimulation (tACS). Brain Stimul..

[55] Najafi K., Fakour Y., Zarrabi H., Heidarzadeh A., Khalkhali M., Yeganeh T., Farahi H., Rostamkhani M., Najafi T., Shabafroz S. (2017). Efficacy of Transcranial Direct Current Stimulation in the Treatment: Resistant Patients who Suffer from Severe Obsessive-compulsive Disorder. Indian J. Psychol. Med..

[56] Bation R., Mondino M., Le Camus F., Saoud M., Brunelin J. (2019). Transcranial direct current stimulation in patients with obsessive compulsive disorder: A randomized controlled trial. Eur. Psychiatry.

[57] Gowda S.M., Narayanaswamy J.C., Hazari N., Bose A., Chhabra H., Balachander S., Bhaskarapillai B., Shivakumar V., Venkatasubramanian G., Reddy Y.C.J. (2019). Efficacy of pre-supplementary motor area transcranial direct current stimulation for treatment resistant obsessive compulsive disorder: A randomized, double blinded, sham controlled trial. Brain Stimul..

[58] Kumar S., Kumar N., Verma R. (2019). Safety and efficacy of adjunctive transcranial direct current stimulation in treatment-resistant obsessive-compulsive disorder: An open-label trial. Indian J. Psychiatry.

[59] Volpato C., Piccione F., Cavinato M., Duzzi D., Schiff S., Foscolo L., Venneri A. (2013). Modulation of affective symptoms and resting state activity by brain stimulation in a treatment-resistant case of obsessive-compulsive disorder. Neurocase.

[60] Mondino M., Haesebaert F., Poulet E., Saoud M., Brunelin J. (2015). Efficacy of Cathodal Transcranial Direct Current Stimulation Over the Left Orbitofrontal Cortex in a Patient With Treatment-Resistant Obsessive-Compulsive Disorder. J. ECT.

[61] Narayanaswamy J.C., Jose D., Chhabra H., Agarwal S.M., Shrinivasa B., Hegde A., Bose A., Kalmady S.V., Venkatasubramanian G., Reddy Y.C. (2015). Successful Application of Add-on Transcranial Direct Current Stimulation (tDCS) for Treatment of SSRI Resistant OCD. Brain Stimul..

[62] Goradel A.J., Pouresmali A., Mowlaie M., Sadeghi Movahed F. (2016). The Effects of Transcranial Direct Current Stimulation on Obsession-compulsion, Anxiety, and Depression of a Patient Suffering from Obsessive-compulsive Disorder. Pract. Clin. Psychol..

[63] D’Urso G., Brunoni A.R., Anastasia A., Micillo M., de Bartolomeis A., Mantovani A. (2016). Polarity-dependent effects of transcranial direct current stimulation in obsessive-compulsive disorder. Neurocase.

[64] Hazari N., Narayanaswamy J.C., Chhabra H., Bose A., Venkatasubramanian G., Reddy Y.C.J. (2016). Response to Transcranial Direct Current Stimulation in a Case of Episodic Obsessive Compulsive Disorder. J. ECT.

[65] Silva R.M., Brunoni A.R., Miguel E.C., Shavitt R.G. (2016). Transcranial direct current stimulation for treatment-resistant obsessive-compulsive disorder: Report on two cases and proposal for a randomized, sham-controlled trial. Sao Paulo Med. J..

[66] Mrakic-Sposta S., Marceglia S., Mameli F., Dilena R., Tadini L., Priori A. (2008). Transcranial direct current stimulation in two patients with Tourette syndrome. Mov. Disord..

[67] Carvalho S., Goncalves O.F., Soares J.M., Sampaio A., Macedo F., Fregni F., Leite J. (2015). Sustained Effects of a Neural-based Intervention in a Refractory Case of Tourette Syndrome. Brain Stimul..

[68] Behler N., Leitner B., Mezger E., Weidinger E., Musil R., Blum B., Kirsch B., Wulf L., Lohrs L., Winter C. (2018). Cathodal tDCS Over Motor Cortex Does Not Improve Tourette Syndrome: Lessons Learned From a Case Series. Front. Behav. Neurosci..

[69] Nitsche M.A., Boggio P.S., Fregni F., Pascual-Leone A. (2009). Treatment of depression with transcranial direct current stimulation (tDCS): A Review. Exp. Neurol..

[70] Sale M.V., Mattingley J.B., Zalesky A., Cocchi L. (2015). Imaging human brain networks to improve the clinical efficacy of non-invasive brain stimulation. Neurosci. Biobehav. Rev..

[71] Palm U., Leitner B., Kirsch B., Behler N., Kumpf U., Wulf L., Padberg F., Hasan A. (2017). Prefrontal tDCS and sertraline in obsessive compulsive disorder: A case report and review of the literature. Neurocase.

[72] Rachid F. (2019). Transcranial direct current stimulation for the treatment of obsessive-compulsive disorder? A qualitative review of safety and efficacy. Psychiatry Res..

[73] Batsikadze G., Moliadze V., Paulus W., Kuo M.F., Nitsche M.A. (2013). Partially non-linear stimulation intensity-dependent effects of direct current stimulation on motor cortex excitability in humans. J. Physiol..

[74] Monte-Silva K., Kuo M.F., Liebetanz D., Paulus W., Nitsche M.A. (2010). Shaping the optimal repetition interval for cathodal transcranial direct current stimulation (tDCS). J. Neurophysiol..

[75] Alonzo A., Brassil J., Taylor J.L., Martin D., Loo C.K. (2012). Daily transcranial direct current stimulation (tDCS) leads to greater increases in cortical excitability than second daily transcranial direct current stimulation. Brain Stimul..

[76] Senco N.M., Huang Y., D’Urso G., Parra L.C., Bikson M., Mantovani A., Shavitt R.G., Hoexter M.Q., Miguel E.C., Brunoni A.R. (2015). Transcranial direct current stimulation in obsessive-compulsive disorder: Emerging clinical evidence and considerations for optimal montage of electrodes. Expert Rev. Med. Devices.

[77] Van den Heuvel O.A., van Wingen G., Soriano-Mas C., Alonso P., Chamberlain S.R., Nakamae T., Denys D., Goudriaan A.E., Veltman D.J. (2016). Brain circuitry of compulsivity. Eur. Neuropsychopharmacol..

[78] Chamberlain S.R.M.A., Fineberg N.A.M.D., Blackwell A.D.P.D., Robbins T.W.P.D., Sahakian S.W.P.D. (2006). Motor Inhibition and Cognitive Flexibility in Obsessive-Compulsive Disorder and Trichotillomania. Am. J. Psychiatry.

[79] Antal A., Paulus W. (2013). Transcranial alternating current stimulation (tACS). Front. Hum. Neurosci..

[80] Tavakoli A.V., Yun K. (2017). Transcranial Alternating Current Stimulation (tACS) Mechanisms and Protocols. Front. Cell. Neurosci..

[81] Hallett M. (2007). Transcranial magnetic stimulation: A primer. Neuron.

[82] Grados M., Huselid R., Duque-Serrano L. (2018). Transcranial Magnetic Stimulation in Tourette Syndrome: A Historical Perspective, Its Current Use and the Influence of Comorbidities in Treatment Response. Brain Sci..

[83] Pell G.S., Roth Y., Zangen A. (2011). Modulation of cortical excitability induced by repetitive transcranial magnetic stimulation: Influence of timing and geometrical parameters and underlying mechanisms. Prog. Neurobiol..

[84] Lenz M., Vlachos A. (2016). Releasing the Cortical Brake by Non-Invasive Electromagnetic Stimulation? rTMS Induces LTD of GABAergic Neurotransmission. Front. Neural Circuits.

[85] Ridding M.C., Rothwell J. (2007). Perspectives—Opinion—Is there a future for therapeutic use of transcranial magnetic stimulation?. Nat. Rev. Neurosci..

[86] Karadag F., Oguzhanoglu N.K., Kurt T., Oguzhanoglu A., Atesci F., Ozdel O. (2003). Quantitative EEG analysis in obsessive compulsive disorder. Int. J. Neurosci..

[87] Loo C.K., Mitchell P.B. (2005). A review of the efficacy of transcranial magnetic stimulation (TMS) treatment for depression, and current and future strategies to optimize efficacy. J. Affect. Disord..

[88] Alonso P., Pujol J., Cardoner N., Benlloch L., Deus J., Menchón J.M., Capdevila A., Vallejo J. (2001). Right Prefrontal Repetitive Transcranial Magnetic Stimulation in Obsessive-Compulsive Disorder: A Double-Blind, Placebo-Controlled Study. Am. J. Psychiatry.

[89] Sachdev P.S., McBride R., Loo C.K., Mitchell P.B., Malhi G.S., Croker V.M. (2001). Right versus left prefrontal transcranial magnetic stimulation for obsessive-compulsive disorder: A preliminary investigation. J. Clin. Psychiatry.

[90] Mantovani A., Lisanby S.H., Pieraccini F., Ulivelli M., Castrogiovanni P., Rossi S. (2006). Repetitive transcranial magnetic stimulation (rTMS) in the treatment of obsessive-compulsive disorder (OCD) and Tourette’s syndrome (TS). Int. J. Neuropsychopharmacol..

[91] Prasko J., Pasková B., Záleský R., Novák T., Kopecek M., Bares M., Horácek J. (2006). The effect of repetitive transcranial magnetic stimulation (rTMS) on symptoms in obsessive compulsive disorder. A randomized, double blind, sham controlled study. Neuro Endocrinol. Lett..

[92] Sachdev P.S., Loo C.K., Mitchell P.B., McFarquhar T.F., Malhi G.S. (2007). Repetitive transcranial magnetic stimulation for the treatment of obsessive compulsive disorder: A double-blind controlled investigation. Psychol. Med..

[93] Kang J.I., Kim C.H., Namkoong K., Lee C.I., Kim S.J. (2009). A randomized controlled study of sequentially applied repetitive transcranial magnetic stimulation in obsessive-compulsive disorder. J. Clin. Psychiatry.

[94] Ruffini C., Locatelli M., Lucca A., Benedetti F., Insacco C., Smeraldi E. (2009). Augmentation effect of repetitive transcranial magnetic stimulation over the orbitofrontal cortex in drug-resistant obsessive-compulsive disorder patients: A controlled investigation. Prim. Care Companion J. Clin. Psychiatry.

[95] Badawy A., El-Sawy H., Abdelhay M. (2010). Efficacy of repetitive transcranial magnetic stimulations in the management of obsessive compulsive disorder. Eur. Neuropsychopharmacol..

[96] Mantovani A., Simpson H.B., Fallon B.A., Rossi S., Lisanby S.H. (2010). Randomized sham-controlled trial of repetitive transcranial magnetic stimulation in treatment-resistant obsessive-compulsive disorder. Int. J. Neuropsychopharmacol..

[97] Sarkhel S., Sinha V.K., Praharaj S.K. (2010). Adjunctive high-frequency right prefrontal repetitive transcranial magnetic stimulation (rTMS) was not effective in obsessive-compulsive disorder but improved secondary depression. J. Anxiety Disord..

[98] Kumar N., Chadda R.K. (2011). Augmentation effect of repetitive transcranial magnetic stimulation over the supplementary motor cortex in treatment refractory patients with obsessive compulsive disorder. Indian J. Psychiatry.

[99] Mansur C.G., Myczkowki M.L., de Barros Cabral S., Sartorelli Mdo C., Bellini B.B., Dias A.M., Bernik M.A., Marcolin M.A. (2011). Placebo effect after prefrontal magnetic stimulation in the treatment of resistant obsessive-compulsive disorder: A randomized controlled trial. Int. J. Neuropsychopharmacol..

[100] Gomes P.V.O., Brasil-Neto J.P., Allam N., de Souza E. (2012). A Randomized, Double-Blind Trial of Repetitive Transcranial Magnetic Stimulation in Obsessive-Compulsive Disorder with Three-Month Follow-Up. J. Neuropsychiatry Clin. Neurosci..

[101] Nauczyciel C., Le Jeune F., Naudet F., Douabin S., Esquevin A., Verin M., Dondaine T., Robert G., Drapier D., Millet B. (2014). Repetitive transcranial magnetic stimulation over the orbitofrontal cortex for obsessive-compulsive disorder: A double-blind, crossover study. Transl. Psychiatry.

[102] Xiaoyan M., Yueqin H., Liwei L., Yi J. (2014). A randomized double-blinded sham-controlled trial of α electroencephalogram-guided transcranial magnetic stimulation for obsessive-compulsive disorder. Chin. Med. J..

[103] Elbeh K.A.M., Elserogy Y.M.B., Khalifa H.E., Ahmed M.A., Hafez M.H., Khedr E.M. (2016). Repetitive transcranial magnetic stimulation in the treatment of obsessive-compulsive disorders: Double blind randomized clinical trial. Psychiatry Res..

[104] Haghighi M., Shayganfard M., Jahangard L., Ahmadpanah M., Bajoghli H., Pirdehghan A., Holsboer-Trachsler E., Brand S. (2015). Repetitive Transcranial Magnetic Stimulation (rTMS) improves symptoms and reduces clinical illness in patients suffering from OCD—Results from a single-blind, randomized clinical trial with sham cross-over condition. J. Psychiatr Res..

[105] Modirrousta M., Shams E., Katz C., Mansouri B., Moussavi Z., Sareen J., Enns M. (2015). The efficacy of deep repetitive transcranial magnetic stimulation over the medial prefrontal cortex in obsessive compulsive disorder: Results from an open-label study. Depress. Anxiety.

[106] Dunlop K., Woodside B., Olmsted M., Colton P., Giacobbe P., Downar J. (2016). Reductions in Cortico-Striatal Hyperconnectivity Accompany Successful Treatment of Obsessive-Compulsive Disorder with Dorsomedial Prefrontal rTMS. Neuropsychopharmacology.

[107] Hawken E.R., Dilkov D., Kaludiev E., Simek S., Zhang F., Milev R. (2016). Transcranial Magnetic Stimulation of the Supplementary Motor Area in the Treatment of Obsessive-Compulsive Disorder: A Multi-Site Study. Int. J. Mol. Sci..

[108] Pallanti S., Marras A., Salerno L., Makris N., Hollander E. (2016). Better than treated as usual: Transcranial magnetic stimulation augmentation in selective serotonin reuptake inhibitor-refractory obsessive-compulsive disorder, mini-review and pilot open-label trial. J. Psychopharmacol..

[109] Pelissolo A., Harika-Germaneau G., Rachid F., Gaudeau-Bosma C., Tanguy M.L., BenAdhira R., Bouaziz N., Popa T., Wassouf I., Saba G. (2016). Repetitive Transcranial Magnetic Stimulation to Supplementary Motor Area in Refractory Obsessive-Compulsive Disorder Treatment: A Sham-Controlled Trial. Int. J. Neuropsychopharmacol..

[110] Seo H.J., Jung Y.E., Lim H.K., Um Y.H., Lee C.U., Chae J.H. (2016). Adjunctive Low-frequency Repetitive Transcranial Magnetic Stimulation over the Right Dorsolateral Prefrontal Cortex in Patients with Treatment-resistant Obsessive-compulsive Disorder: A Randomized Controlled Trial. Clin. Psychopharmacol. Neurosci..

[111] Donse L., Sack A.T., Fitzgerald P.B., Arns M. (2017). Sleep disturbances in obsessive-compulsive disorder: Association with non-response to repetitive transcranial magnetic stimulation (rTMS). J. Anxiety Disord..

[112] Lee Y.J., Koo B.H., Seo W.S., Kim H.G., Kim J.Y., Cheon E.J. (2017). Repetitive transcranial magnetic stimulation of the supplementary motor area in treatment-resistant obsessive-compulsive disorder: An open-label pilot study. J. Clin. Neurosci..

[113] Arumugham S.S., Subhasini V.S., Madhuri H.N., Vinay B., Ravi M., Sharma E., Thirthalli J., Reddy Y.J. (2018). Augmentation Effect of Low-Frequency Repetitive Transcranial Magnetic Stimulation Over Presupplementary Motor Area in Obsessive-Compulsive Disorder: A Randomized Controlled Trial. J. ECT.

[114] Carmi L., Alyagon U., Barnea-Ygael N., Zohar J., Dar R., Zangen A. (2018). Clinical and electrophysiological outcomes of deep TMS over the medial prefrontal and anterior cingulate cortices in OCD patients. Brain Stimul..

[115] Kumar S., Singh S., Chadda R.K., Verma R., Kumar N. (2018). The Effect of Low-Frequency Repetitive Transcranial Magnetic Stimulation at Orbitofrontal Cortex in the Treatment of Patients with Medication-Refractory Obsessive-Compulsive Disorder: A Retrospective Open Study. J. ECT.

[116] Carmi L., Tendler A., Bystritsky A., Hollander E., Blumberger D.M., Daskalakis J., Ward H., Lapidus K., Goodman W., Casuto L. (2019). Efficacy and Safety of Deep Transcranial Magnetic Stimulation for Obsessive-Compulsive Disorder: A Prospective Multicenter Randomized Double-Blind Placebo-Controlled Trial. Am. J. Psychiatry.

[117] Harika-Germaneau G., Rachid F., Chatard A., Lafay-Chebassier C., Solinas M., Thirioux B., Millet B., Langbour N., Jaafari N. (2019). Continuous theta burst stimulation over the supplementary motor area in refractory obsessive-compulsive disorder treatment: A randomized sham-controlled trial. Brain Stimul..

[118] Singh S., Kumar S., Gupta A., Verma R., Kumar N. (2019). Effectiveness and Predictors of Response to 1-Hz Repetitive Transcranial Magnetic Stimulation in Patients with Obsessive-Compulsive Disorder. J. ECT.

[119] Talaei A., Morteza-NIA M., Jafar-Zadeh M., Saghebi A., Rezaei A.A. (2009). Dramatic response of resistant obsessive compulsive disorder to repeated trancranial magnetic stimulation on right supplementary motor area. Iran. J. Med Sci..

[120] Mantovani A., Westin G., Hirsch J., Lisanby S.H. (2009). Functional magnetic resonance imaging guided transcranial magnetic stimulation in obsessive-compulsive disorder. Biol. Psychiatry.

[121] Wu C.C., Tsai C.C., Lu M.K., Chen C.M., Shen W.C., Su K.P. (2010). Theta-Burst Repetitive Transcranial Magnetic Stimulation for Treatment-Resistant Obsessive-Compulsive Disorder with Concomitant Depression. J. Clin. Psychiatry.

[122] Winkelbeiner S., Suker S., Bachofner H., Eisenhardt S., Steinau S., Walther S. (2018). Targeting Obsessive-Compulsive Symptoms With rTMS and Perfusion Imaging. Am. J. Psychiatry.

[123] Kar S.K., Dwivedi S., Agarwal V. (2019). Relevance of extended protocol and maintenance TMS in obsessive-compulsive disorder: A case report. Asian J. Psychiatr.

[124] Chae J.-H., Nahas Z., Wassermann E., Li X., Sethuraman G., Gilbert D., Sallee F.R., George M.S. (2004). A Pilot Safety Study of Repetitive Transcranial Magnetic Stimulation (rTMS) in Tourette’s Syndrome. Cogn. Behav. Neurol..

[125] Orth M., Kirby R., Richardson M.P., Snijders A.H., Rothwell J.C., Trimble M.R., Robertson M.M., Munchau A. (2005). Subthreshold rTMS over pre-motor cortex has no effect on tics in patients with Gilles de la Tourette syndrome. Clin. Neurophysiol..

[126] Kwon H.J., Lim W.S., Lim M.H., Lee S.J., Hyun J.K., Chae J.H., Paik K.C. (2011). 1-Hz low frequency repetitive transcranial magnetic stimulation in children with Tourette’s syndrome. Neurosci. Lett..

[127] Le K., Liu L., Sun M., Hu L., Xiao N. (2013). Transcranial magnetic stimulation at 1 Hertz improves clinical symptoms in children with Tourette syndrome for at least 6 months. J. Clin. Neurosci..

[128] Wu S.W., Maloney T., Gilbert D.L., Dixon S.G., Horn P.S., Huddleston D.A., Eaton K., Vannest J. (2014). Functional MRI-navigated repetitive transcranial magnetic stimulation over supplementary motor area in chronic tic disorders. Brain Stimul..

[129] Landeros-Weisenberger A., Mantovani A., Motlagh M.G., de Alvarenga P.G., Katsovich L., Leckman J.F., Lisanby S.H. (2015). Randomized Sham Controlled Double-blind Trial of Repetitive Transcranial Magnetic Stimulation for Adults With Severe Tourette Syndrome. Brain Stimul..

[130] Bloch Y., Arad S., Levkovitz Y. (2016). Deep TMS add-on treatment for intractable Tourette syndrome: A feasibility study. World J. Biol. Psychiatry.

[131] Mantovani A., Leckman J.F., Grantz H., King R.A., Sporn A.L., Lisanby S.H. (2007). Repetitive Transcranial Magnetic Stimulation of the Supplementary Motor Area in the treatment of Tourette Syndrome: Report of two cases. Clin. Neurophysiol..

[132] Salatino A., Momo E., Nobili M., Berti A., Ricci R. (2014). Awareness of symptoms amelioration following low-frequency repetitive transcranial magnetic stimulation in a patient with Tourette syndrome and comorbid obsessive-compulsive disorder. Brain Stimul..

[133] Aydin E.P., Kenar J.G., Altunay I.K., Kaymak D., Ozer O.A., Karamustafalioglu K.O. (2020). Repetitive Transcranial Magnetic Stimulation in the Treatment of Skin Picking Disorder: An Exploratory Trial. J. ECT.

[134] Diefenbach G.J., Tolin D.F., Hallion L.S., Zertuche L., Rabany L., Goethe J.W., Assaf M. (2015). A case study of clinical and neuroimaging outcomes following repetitive transcranial magnetic stimulation for hoarding disorder. Am. J. Psychiatry.

[135] Fitzgerald P.B., Hoy K.E., Elliot D., Susan McQueen R.N., Wambeek L.E., Daskalakis Z.J. (2018). Accelerated repetitive transcranial magnetic stimulation in the treatment of depression. Neuropsychopharmacology.

[136] Loo C.K., Taylor J.L., Gandevia S.C., McDarmont B.N., Mitchell P.B., Sachdev P.S. (2000). Transcranial magnetic stimulation (TMS) in controlled treatment studies: Are some “sham” forms active?. Biol. Psychiatry.

[137] Lisanby S.H., Gutman D., Luber B., Schroeder C., Sackeim H.A. (2001). Sham TMS: Intracerebral measurement of the induced electrical field and the induction of motor-evoked potentials. Biol. Psychiatry.

[138] Duecker F., Sack A.T. (2015). Rethinking the role of sham TMS. Front. Psychol..

[139] Rehn S., Eslick G.D., Brakoulias V. (2018). A Meta-Analysis of the Effectiveness of Different Cortical Targets Used in Repetitive Transcranial Magnetic Stimulation (rTMS) for the Treatment of Obsessive-Compulsive Disorder (OCD). Psychiatr Q..

[140] Chen J., Zhou C., Wu B., Wang Y., Li Q., Wei Y., Yang D., Mu J., Zhu D., Zou D. (2013). Left versus right repetitive transcranial magnetic stimulation in treating major depression: A meta-analysis of randomised controlled trials. Psychiatry Res..

[141] Teng S., Guo Z., Peng H., Xing G., Chen H., He B., McClure M.A., Mu Q. (2017). High-frequency repetitive transcranial magnetic stimulation over the left DLPFC for major depression: Session-dependent efficacy: A meta-analysis. Eur. Psychiatry.

[142] de Wit S.J., de Vries F.E., van der Werf Y.D., Cath D.C., Heslenfeld D.J., Veltman E.M., van Balkom A.J., Veltman D.J., van den Heuvel O.A. (2012). Presupplementary Motor Area Hyperactivity During Response Inhibition: A Candidate Endophenotype of Obsessive-Compulsive Disorder. Am. J. Psychiatry.

[143] Saxena S., Rauch S.L. (2000). Functional Neuroimaging and the Neuroanatomy of Obsessive-Compulsive Disorder. Psychiatr. Clin. N. Am..

[144] Miller E.K., Cohen J.D. (2001). An Integrative Theory of Prefrontal Cortex Function. Annu. Rev. Neurosci..

[145] Roth Y., Amir A., Levkovitz Y., Zangen A. (2007). Three-dimensional distribution of the electric field induced in the brain by transcranial magnetic stimulation using figure-8 and deep H-coils. J. Clin. Neurophysiol..

[146] Bersani F.S., Minichino A., Enticott P.G., Mazzarini L., Khan N., Antonacci G., Raccah R.N., Salviati M., Delle Chiaie R., Bersani G. (2013). Deep transcranial magnetic stimulation as a treatment for psychiatric disorders: A comprehensive review. Eur. Psychiatry.

[147] Denys D., Schuurman P.R. (2012). Basic principles of deep brain stimulation. Deep Brain Stimulation, a New Frontier in Psychiatry.

[148] Grill W.M., Snyder A.N., Miocinovic S. (2004). Deep brain stimulation creates an informational lesion of the stimulated nucleus. NeuroReport.

[149] McIntyre C.C., Anderson R.W. (2016). Deep brain stimulation mechanisms: The control of network activity via neurochemistry modulation. J. Neurochem..

[150] Wagle Shukla A., Zeilman P., Fernandez H., Bajwa J.A., Mehanna R. (2017). DBS Programming: An Evolving Approach for Patients with Parkinson’s Disease. Parkinson’s Dis..

[151] Castrioto A., Volkmann J., Krack P. (2013). Postoperative management of deep brain stimulation in Parkinson’s disease. Handb. Clin. Neurol..

[152] Gabriels L., Cosyns P., Nuttin B., Demeulemeester H., Gybels J. (2003). Deep brain stimulation for treatment-refractory obsessive-compulsive disorder: Psychopathological and neuropsychological outcome in three cases. Acta Psychiatr. Scand..

[153] Nuttin B.J., Gabriels L.A., Cosyns P.R., Meyerson B.A., Andreewitch S., Sunaert S.G., Maes A.F., Dupont P.J., Gybels J.M., Gielen F. (2003). Long-term electrical capsular stimulation in patients with obsessive-compulsive disorder. Neurosurgery.

[154] Greenberg B.D., Malone D.A., Friehs G.M., Rezai A.R., Kubu C.S., Malloy P.F., Salloway S.P., Okun M.S., Goodman W.K., Rasmussen S.A. (2006). Three-year outcomes in deep brain stimulation for highly resistant obsessive-compulsive disorder. Neuropsychopharmacology.

[155] Greenberg B.D., Gabriels L.A., Malone D.A., Rezai A.R., Friehs G.M., Okun M.S., Shapira N.A., Foote K.D., Cosyns P.R., Kubu C.S. (2010). Deep brain stimulation of the ventral internal capsule/ventral striatum for obsessive-compulsive disorder: Worldwide experience. Mol. Psychiatry.

[156] Luyten L., Hendrickx S., Raymaekers S., Gabriels L., Nuttin B. (2016). Electrical stimulation in the bed nucleus of the stria terminalis alleviates severe obsessive-compulsive disorder. Mol. Psychiatry.

[157] Abelson J.L., Curtis G.C., Sagher O., Albucher R.C., Harrigan M., Taylor S.F., Martis B., Giordani B. (2005). Deep brain stimulation for refractory obsessive-compulsive disorder. Biol. Psychiatry.

[158] Mallet L., Polosan M., Jaafari N., Baup N., Welter M.-L., Fontaine D., Montcel S.T.d., Yelnik J., Chéreau I., Arbus C. (2008). Subthalamic Nucleus Stimulation in Severe Obsessive–Compulsive Disorder. N. Engl. J. Med..

[159] Goodman W.K., Foote K.D., Greenberg B.D., Ricciuti N., Bauer R., Ward H., Shapira N.A., Wu S.S., Hill C.L., Rasmussen S.A. (2010). Deep brain stimulation for intractable obsessive compulsive disorder: Pilot study using a blinded, staggered-onset design. Biol. Psychiatry.

[160] Fayad S.M., Guzick A.G., Reid A.M., Mason D.M., Bertone A., Foote K.D., Okun M.S., Goodman W.K., Ward H.E. (2016). Six-Nine Year Follow-Up of Deep Brain Stimulation for Obsessive-Compulsive Disorder. PLoS ONE.

[161] Huff W., Lenartz D., Schormann M., Lee S.H., Kuhn J., Koulousakis A., Mai J., Daumann J., Maarouf M., Klosterkotter J. (2010). Unilateral deep brain stimulation of the nucleus accumbens in patients with treatment-resistant obsessive-compulsive disorder: Outcomes after one year. Clin. Neurol. Neurosurg..

[162] Mantione M., Nieman D.H., Figee M., Denys D. (2014). Cognitive-behavioural therapy augments the effects of deep brain stimulation in obsessive-compulsive disorder. Psychol. Med..

[163] Islam L., Franzini A., Messina G., Scarone S., Gambini O. (2015). Deep brain stimulation of the nucleus accumbens and bed nucleus of stria terminalis for obsessive-compulsive disorder: A case series. World Neurosurg..

[164] Farrand S., Evans A.H., Mangelsdorf S., Loi S.M., Mocellin R., Borham A., Bevilacqua J., Blair-West S., Walterfang M.A., Bittar R.G. (2018). Deep brain stimulation for severe treatment-resistant obsessive-compulsive disorder: An open-label case series. Aust. N. Z. J. Psychiatry.

[165] Barcia J.A., Avecillas-Chasin J.M., Nombela C., Arza R., Garcia-Albea J., Pineda-Pardo J.A., Reneses B., Strange B.A. (2019). Personalized striatal targets for deep brain stimulation in obsessive-compulsive disorder. Brain Stimul..

[166] Lee D.J., Dallapiazza R.F., De Vloo P., Elias G.J.B., Fomenko A., Boutet A., Giacobbe P., Lozano A.M. (2019). Inferior thalamic peduncle deep brain stimulation for treatment-refractory obsessive-compulsive disorder: A phase 1 pilot trial. Brain Stimul..

[167] Huys D., Kohl S., Baldermann J.C., Timmermann L., Sturm V., Visser-Vandewalle V., Kuhn J. (2019). Open-label trial of anterior limb of internal capsule-nucleus accumbens deep brain stimulation for obsessive-compulsive disorder: Insights gained. J. Neurol. Neurosurg. Psychiatry.

[168] Tyagi H., Apergis-Schoute A.M., Akram H., Foltynie T., Limousin P., Drummond L.M., Fineberg N.A., Matthews K., Jahanshahi M., Robbins T.W. (2019). A Randomized Trial Directly Comparing Ventral Capsule and Anteromedial Subthalamic Nucleus Stimulation in Obsessive-Compulsive Disorder: Clinical and Imaging Evidence for Dissociable Effects. Biol. Psychiatry.

[169] Burdick A., Foote K.D., Goodman W., Ward H.E., Ricciuti N., Murphy T., Haq I., Okun M.S. (2010). Lack of benefit of accumbens/capsular deep brain stimulation in a patient with both tics and obsessive-compulsive disorder. Neurocase.

[170] Franzini A., Messina G., Gambini O., Muffatti R., Scarone S., Cordella R., Broggi G. (2010). Deep-brain stimulation of the nucleus accumbens in obsessive compulsive disorder: Clinical, surgical and electrophysiological considerations in two consecutive patients. Neurol. Sci..

[171] Grant J.E., Odlaug B.L., Chamberlain S.R. (2011). Neurocognitive Response to Deep Brain Stimulation for Obsessive-Compulsive Disorder: A Case Report. Am. J. Psychiatry.

[172] Roh D., Chang W.S., Chang J.W., Kim C.H. (2012). Long-term follow-up of deep brain stimulation for refractory obsessive-compulsive disorder. Psychiatry Res..

[173] Coenen V.A., Schlaepfer T.E., Sajonz B., Dobrossy M., Kaller C.P., Urbach H., Reisert M. (2020). Tractographic description of major subcortical projection pathways passing the anterior limb of the internal capsule. Corticopetal organization of networks relevant for psychiatric disorders. Neuroimage Clin..

[174] Tsai C.H., Chang C.H., Pan J.I., Hsieh H.J., Tsai S.T., Hung H.Y. (2014). Acute stimulation effect of the ventral capsule/ventral striatum in patients with refractory obsessive-compulsive disorder-a double-blinded trial. Neuropsychiatr. Dis. Treat..

[175] Maarouf M., Neudorfer C., El Majdoub F., Lenartz D., Kuhn J., Sturm V. (2016). Deep Brain Stimulation of Medial Dorsal and Ventral Anterior Nucleus of the Thalamus in OCD: A Retrospective Case Series. PLoS ONE.

[176] Chang C.H., Chen S.Y., Tsai S.T., Tsai H.C. (2017). Compulsive skin-picking behavior after deep brain. Medicine.

[177] Choudhury T.K., Davidson J.E., Viswanathan A., Strutt A.M. (2017). Deep brain stimulation of the anterior limb of the internal capsule for treatment of therapy-refractory obsessive compulsive disorder (OCD): A case study highlighting neurocognitive and psychiatric changes. Neurocase.

[178] Gupta A., Khanna S., Jain R. (2019). Deep brain stimulation of ventral internal capsule for refractory obsessive-compulsive disorder. Indian J. Psychiatry.

[179] Maciunas R.J., Maddux B.N., Riley D.E., Whitney C.M., Schoenberg M.R., Ogrocki P.J., Albert J.M., Gould D.J. (2007). Prospective randomized double-blind trial of bilateral thalamic deep brain stimulation in adults with Tourette syndrome. J. Neurosurg..

[180] Servello D., Porta M., Sassi M., Brambilla A., Robertson M.M. (2008). Deep brain stimulation in 18 patients with severe Gilles de la Tourette syndrome refractory to treatment: The surgery and stimulation. J. Neurol. Neurosurg. Psychiatry.

[181] Porta M., Brambilla A., Cavanna A.E., Servello D., Sassi M., Rickards H., Robertson M.M. (2009). Thalamic deep brain stimulation for treatment-refractory Tourette syndrome. Two Year Outcome.

[182] Porta M., Servello D., Zanaboni C., Anasetti F., Menghetti C., Sassi M., Robertson M.M. (2012). Deep brain stimulation for treatment of refractory Tourette syndrome: Long-term follow-up. Acta Neurochir..

[183] Ackermans L., Duits A., van der Linden C., Tijssen M., Schruers K., Temel Y., Kleijer M., Nederveen P., Bruggeman R., Tromp S. (2011). Double-blind clinical trial of thalamic stimulation in patients with Tourette syndrome. Brain.

[184] Cannon E., Silburn P., Coyne T., O’Maley K., Crawford J.D., Sachdev P.S. (2012). Deep Brain Stimulation of Anteromedial Globus Pallidus Interna for Severe Tourette’s Syndrome. Am. J. Psychiatry.

[185] Sachdev P.S., Mohan A., Cannon E., Crawford J.D., Silberstein P., Cook R., Coyne T., Silburn P.A. (2014). Deep brain stimulation of the antero-medial globus pallidus interna for Tourette syndrome. PLoS ONE.

[186] Motlagh M.G., Smith M.E., Landeros-Weisenberger A., Kobets A.J., King R.A., Miravite J., de Lotbinière A.C., Alterman R.L., Mogilner A.Y., Pourfar M.H. (2013). Lessons Learned from Open-label Deep Brain Stimulation for Tourette Syndrome: Eight Cases over 7 Years. Tremor Other Hyperkinet. Mov..

[187] Okun M.S., Foote K.D., Wu S.S., Ward H.E., Bowers D., Rodriguez R.L., Malaty I.A., Goodman W.K., Gilbert D.M., Walker H.C. (2013). A trial of scheduled deep brain stimulation for Tourette syndrome: Moving away from continuous deep brain stimulation paradigms. JAMA Neurol..

[188] Rossi P.J., Opri E., Shute J.B., Molina R., Bowers D., Ward H., Foote K.D., Gunduz A., Okun M.S. (2016). Scheduled, intermittent stimulation of the thalamus reduces tics in Tourette syndrome. Parkinsonism Relat. Disord..

[189] Zhang J.G., Ge Y., Stead M., Zhang K., Yan S.S., Hu W., Meng F.G. (2014). Long-term outcome of globus pallidus internus deep brain stimulation in patients with Tourette syndrome. Mayo Clin. Proc..

[190] Kefalopoulou Z., Zrinzo L., Jahanshahi M., Candelario J., Milabo C., Beigi M., Akram H., Hyam J., Clayton J., Kass-Iliyya L. (2015). Bilateral globus pallidus stimulation for severe Tourette’s syndrome: A double-blind, randomised crossover trial. Lancet Neurol..

[191] Huys D., Bartsch C., Koester P., Lenartz D., Maarouf M., Daumann J., Mai J.K., Klosterkotter J., Hunsche S., Visser-Vandewalle V. (2016). Motor Improvement and Emotional Stabilization in Patients With Tourette Syndrome After Deep Brain Stimulation of the Ventral Anterior and Ventrolateral Motor Part of the Thalamus. Biol. Psychiatry.

[192] Testini P., Zhao C.Z., Stead M., Duffy P.S., Klassen B.T., Lee K.H. (2016). Centromedian-Parafascicular Complex Deep Brain Stimulation for Tourette Syndrome: A Retrospective Study. Mayo Clin. Proc..

[193] Welter M.-L., Houeto J.-L., Thobois S., Bataille B., Guenot M., Worbe Y., Hartmann A., Czernecki V., Bardinet E., Yelnik J. (2017). Anterior pallidal deep brain stimulation for Tourette’s syndrome: A randomised, double-blind, controlled trial. Lancet Neurol..

[194] Welter M.L., Houeto J.L., Worbe Y., Diallo M.H., Hartmann A., Tezenas du Montcel S., Ansquer S., Thobois S., Fontaine D., Rouaud T. (2019). Long-term effects of anterior pallidal deep brain stimulation for tourette’s syndrome. Mov. Disord..

[195] Azimi A., Parvaresh M., Shahidi G., Habibi A., Rohani S., Safdarian M., Fattahi A., Taheri M., Rohani M. (2018). Anteromedial GPi deep brain stimulation in Tourette syndrome: The first case series from Iran. Clin. Neurol. Neurosurg..

[196] Brito M., Teixeira M.J., Mendes M.M., Franca C., Iglesio R., Barbosa E.R., Cury R.G. (2019). Exploring the clinical outcomes after deep brain stimulation in Tourette syndrome. J. Neurol. Sci..

[197] Diederich N.J., Kalteis K., Stamenkovic M., Pieri V., Alesch F. (2005). Efficient internal pallidal stimulation in Gilles de la Tourette syndrome: A case report. Mov. Disord..

[198] Flaherty A.W., Williams Z.M., Amirnovin R., Kasper E., Rauch S.L., Cosgrove G.R., Eskandar E.N. (2005). Deep brain stimulation of the anterior internal capsule for the treatment of Tourette syndrome: Technical case report. Neurosurgery.

[199] Houeto J.L., Karachi C., Mallet L., Pillon B., Yelnik J., Mesnage V., Welter M.L., Navarro S., Pelissolo A., Damier P. (2005). Tourette’s syndrome and deep brain stimulation. J. Neurol. Neurosurg. Psychiatry.

[200] Kuhn J., Lenartz D., Mai J.K., Huff W., Lee S.H., Koulousakis A., Klosterkoetter J., Sturm V. (2007). Deep brain stimulation of the nucleus accumbens and the internal capsule in therapeutically refractory Tourette-syndrome. J. Neurol..

[201] Shahed J., Poysky J., Kenney C., Simpson R., Jankovic J. (2007). GPi deep brain stimulation for Tourette syndrome improves tics and psychiatric comorbidities. Neurology.

[202] Dehning S., Mehrkens J.H., Muller N., Botzel K. (2008). Therapy-refractory Tourette syndrome: Beneficial outcome with globus pallidus internus deep brain stimulation. Mov. Disord..

[203] Shields D.C., Cheng M.L., Flaherty A.W., Gale J.T., Eskander E.N. (2008). Microelectrode-guided deep brain stimulation for Tourette syndrome: Within-subject comparison of different stimulation sites. Stereotact. Funct. Neurosurg..

[204] Welter M.-L., Mallet L., Houeto J.-L., Karachi C., Czernecki V., Cornu P., Navarro S., Pidoux B., Dormont D., Bardinet E. (2008). Internal Pallidal and Thalamic Stimulation in Patients with Tourette Syndrome. Arch. Neurol..

[205] Martinez-Fernandez R., Zrinzo L., Aviles-Olmos I., Hariz M., Martinez-Torres I., Joyce E., Jahanshahi M., Limousin P., Foltynie T. (2011). Deep brain stimulation for Gilles de la Tourette syndrome: A case series targeting subregions of the globus pallidus internus. Mov. Disord..

[206] Pullen S.J., Wall C.A., Lee K.H., Stead S.M., Klassen B.T., Brown T.M. (2011). Neuropsychiatric Outcome of an Adolescent Who Received Deep Brain Stimulation for Tourette’s Syndrome. Case Rep. Neurol. Med..

[207] Rzesnitzek L., Wachter T., Kruger R., Gharabaghi A., Plewnia C. (2011). Suppression of extrapyramidal side effects of doxepin by thalamic deep brain stimulation for Tourette syndrome. Neurology.

[208] Savica R., Stead M., Mack K.J., Lee K.H., Klassen B.T. (2012). Deep brain stimulation in tourette syndrome: A description of 3 patients with excellent outcome. Mayo Clin. Proc..

[209] Massano J., Sousa C., Foltynie T., Zrinzo L., Hariz M., Vaz R. (2013). Successful pallidal deep brain stimulation in 15-year-old with Tourette syndrome: 2-year follow-up. J. Neurol..

[210] Piedimonte F., Andreani J.C., Piedimonte L., Graff P., Bacaro V., Micheli F., Vilela Filho O. (2013). Behavioral and motor improvement after deep brain stimulation of the globus pallidus externus in a case of Tourette’s syndrome. Neuromodulation.

[211] Dong S., Zhang X., Li J., Li Y. (2014). The benefits of low-frequency pallidal deep brain stimulation in a patient with Tourette syndrome. Parkinsonism Relat. Disord..

[212] Huasen B., McCreary R., Evans J., Potter G., Silverdale M. (2014). Cervical myelopathy secondary to Tourette’s syndrome managed by urgent deep brain stimulation. Mov. Disord..

[213] Nair G., Evans A., Bear R.E., Velakoulis D., Bittar R.G. (2014). The anteromedial GPi as a new target for deep brain stimulation in obsessive compulsive disorder. J. Clin. Neurosci..

[214] Patel N., Jimenez-Shahed J. (2014). Simultaneous improvement of tics and parkinsonism after pallidal DBS. Parkinsonism Relat. Disord..

[215] Wojtecki L., Elben S., Rubenach J., Hartmann C., Vesper J., Schnitzler A. (2016). Subthalamic Deep Brain Stimulation in Obsessive-Compulsive Disorder: First German Experience and Future Outlook. World Neurosurg..

[216] Kano Y., Matsuda N., Nonaka M., Fujio M., Kono T., Kaido T. (2018). Sensory phenomena and obsessive-compulsive symptoms in Tourette syndrome following deep brain stimulation: Two case reports. J. Clin. Neurosci..

[217] Kakusa B., Saluja S., Tate W.J., Espil F.M., Halpern C.H., Williams N.R. (2019). Robust clinical benefit of multi-target deep brain stimulation for treatment of Gilles de la Tourette syndrome and its comorbidities. Brain Stimul..

[218] Rossi M., Cerquetti D., Cammarota A., Merello M. (2019). Tourette syndrome: Clinical benefit with unilateral stimulation after bilateral pallidal implant. Mov. Disord..

[219] Zhu G.Y., Geng X.Y., Zhang R.L., Chen Y.C., Liu Y.Y., Wang S.Y. (2019). Deep brain stimulation modulates pallidal and subthalamic neural oscillations in Tourette’s syndrome. Brain Behav..

[220] Liebrand L.C., Caan M.W.A., Schuurman P.R., van den Munckhof P., Figee M., Denys D., van Wingen G.A. (2019). Individual white matter bundle trajectories are associated with deep brain stimulation response in obsessive-compulsive dis-order. Brain Stimul..

[221] Baldermann J.C., Melzer C., Zapf A., Kohl S., Timmermann L., Tittgemeyer M., Huys D., Visser-Vandewalle V., Kuhn A.A., Horn A. (2019). Connectivity Profile Predictive of Effective Deep Brain Stimulation in Obsessive-Compulsive Disorder. Biol. Psychiatry.

[222] van Westen M., Rietveld E., Bergfeld I.O., de Koning P., Vullink N., Ooms P., Graat I., Liebrand L., van den Munckhof P., Schuurman R. (2020). Optimizing Deep Brain Stimulation Parameters in Obsessive-Compulsive Disorder. Neuromodulation Technol. Neural Interface.

[223] Koeglsperger T., Palleis C., Hell F., Mehrkens J.H., Bötzel K. (2019). Deep Brain Stimulation Programming for Movement Disorders: Current Concepts and Evidence-Based Strategies. Front. Neurol..

[224] Haber S.N., Yendiki A., Jbabdi S. (2020). Four Deep Brain Stimulation Targets for Obsessive-Compulsive Disorder: Are They Different?. Biol. Psychiatry.

[225] Suetens K., Nuttin B., Gabriels L., Van Laere K. (2014). Differences in metabolic network modulation between capsulotomy and deep-brain stimulation for refractory obses-sive-compulsive disorder. J. Nucl. Med..

[226] Denys D., Graat I., Mocking R., de Koning P., Vulink N., Figee M., Ooms P., Mantione M., van den Munckhof P., Schuurman R. (2020). Efficacy of Deep Brain Stimulation of the Ventral Anterior Limb of the Internal Capsule for Refractory Obsessive-Compulsive Disorder: A Clinical Cohort of 70 Patients. Am. J. Psychiatry.

[227] Moro E., Poon Y.-Y.W., Lozano A.M., Saint-Cyr J.A., Lang A.E. (2006). Subthalamic Nucleus Stimulation: Improvements in Outcome with Reprogramming. Arch. Neurol..

[228] Farris S., Giroux M. (2013). Retrospective review of factors leading to dissatisfaction with subthalamic nucleus deep brain stimulation during long-term management. Surg. Neurol. Int..

[229] Li N., Baldermann J.C., Kibleur A., Treu S., Akram H., Elias G.J.B., Boutet A., Lozano A.M., Al-Fatly B., Strange B. (2020). A unified connectomic target for deep brain stimulation in obsessive-compulsive disorder. Nat. Commun..

[230] Horn A., Fox M.D. (2020). Opportunities of connectomic neuromodulation. Neuroimage.

[231] Middlebrooks E.H., Domingo R.A., Vivas-Buitrago T., Okromelidze L., Tsuboi T., Wong J.K., Eisinger R.S., Almeida L., Burns M.R., Horn A. (2020). Neuroimaging Advances in Deep Brain Stimulation: Review of Indications, Anatomy, and Brain Connectomics. AJNR Am. J. Neuroradiol..

[232] Hartmann C.J., Lujan J.L., Chaturvedi A., Goodman W.K., Okun M.S., McIntyre C.C., Haq I.U. (2015). Tractography Activation Patterns in Dorsolateral Prefrontal Cortex Suggest Better Clinical Responses in OCD DBS. Front. Neurosci..

[233] Chabardes S., Polosan M., Krack P., Bastin J., Krainik A., David O., Bougerol T., Benabid A.L. (2013). Deep brain stimulation for obsessive-compulsive disorder: Subthalamic nucleus target. World Neurosurg..

[234] Lee P.S., Weiner G.M., Corson D., Kappel J., Chang Y.F., Suski V.R., Berman S.B., Homayoun H., Van Laar A.D., Crammond D.J. (2018). Outcomes of Interventional-MRI Versus Microelectrode Recording-Guided Subthalamic Deep Brain Stimulation. Front. Neurol..

[235] Johnson K.A., Duffley G., Anderson D.N., Ostrem J.L., Welter M.L., Baldermann J.C., Kuhn J., Huys D., Visser-Vandewalle V., Foltynie T. (2020). Structural connectivity predicts clinical outcomes of deep brain stimulation for Tourette syndrome. Brain.

[236] Martinez-Ramirez D., Jimenez-Shahed J., Leckman J.F., Porta M., Servello D., Meng F.G., Kuhn J., Huys D., Baldermann J.C., Foltynie T. (2018). Efficacy and Safety of Deep Brain Stimulation in Tourette Syndrome: The International Tourette Syndrome Deep Brain Stimulation Public Database and Registry. JAMA Neurol..

[237] Johnson K.A., Fletcher P.T., Servello D., Bona A., Porta M., Ostrem J.L., Bardinet E., Welter M.L., Lozano A.M., Baldermann J.C. (2019). Image-based analysis and long-term clinical outcomes of deep brain stimulation for Tourette syndrome: A multisite study. J. Neurol. Neurosurg. Psychiatry.

[238] Baldermann J.C., Schuller T., Huys D., Becker I., Timmermann L., Jessen F., Visser-Vandewalle V., Kuhn J. (2016). Deep Brain Stimulation for Tourette-Syndrome: A Systematic Review and Meta-Analysis. Brain Stimul..

[239] Pedroarena-Leal N., Ruge D. (2017). Toward a Symptom-Guided Neurostimulation for Gilles de la Tourette Syndrome. Front. Psychiatry.

[240] Fineberg N.A., Hollander E., Pallanti S., Walitza S., Grunblatt E., Dell’Osso B.M., Albert U., Geller D.A., Brakoulias V., Janardhan Reddy Y.C. (2020). Clinical advances in obsessive-compulsive disorder: A position statement by the International College of Obsessive-Compulsive Spectrum Disorders. Int. Clin. Psychopharmacol..

[241] da Silva R.M.F., Batistuzzo M.C., Shavitt R.G., Miguel E.C., Stern E., Mezger E., Padberg F., D’Urso G., Brunoni A.R. (2019). Transcranial direct current stimulation in obsessive-compulsive disorder: An update in electric field modeling and investigations for optimal electrode montage. Expert Rev. Neurother..

[242] D’Urso G., Mantovani A., Patti S., Toscano E., de Bartolomeis A. (2018). Transcranial Direct Current Stimulation in Obsessive-Compulsive Disorder, Posttraumatic Stress Disorder, and Anxiety Disorders. J. ECT.

[243] Jacobson L., Koslowsky M., Lavidor M. (2012). tDCS polarity effects in motor and cognitive domains: A meta-analytical review. Exp. Brain Res..

[244] da Silva R.M.F., Brunoni A.R., Miguel E.C., Shavitt R.G. (2019). Transcranial direct current stimulation for Obsessive-Compulsive Disorder: Patient selection and perspectives. Neuropsychiatr. Dis. Treat..

[245] Zhou D.D., Wang W., Wang G.M., Li D.Q., Kuang L. (2017). An updated meta-analysis: Short-term therapeutic effects of repeated transcranial magnetic stimulation in treating obsessive-compulsive disorder. J. Affect. Disord..

[246] Morishita T., Fayad S.M., Goodman W.K., Foote K.D., Chen D., Peace D.A., Rhoton A.L., Okun M.S. (2014). Surgical neuroanatomy and programming in deep brain stimulation for obsessive compulsive disorder. Neuromodulation.

[247] Karas P.J., Lee S., Jimenez-Shahed J., Goodman W.K., Viswanathan A., Sheth S.A. (2018). Deep Brain Stimulation for Obsessive Compulsive Disorder: Evolution of Surgical Stimulation Target Parallels Changing Model of Dysfunctional Brain Circuits. Front. Neurosci..

[248] Deeb W., Rossi P.J., Porta M., Visser-Vandewalle V., Servello D., Silburn P., Coyne T., Leckman J.F., Foltynie T., Hariz M. (2016). The International Deep Brain Stimulation Registry and Database for Gilles de la Tourette Syndrome: How Does It Work?. Front. Neurosci..

[249] Bosanac P., Hamilton B.E., Lucak J., Castle D. (2018). Identity challenges and ‘burden of normality’ after DBS for severe OCD: A narrative case study. BMC Psychiatry.

[250] Nam S.K., Yoo D., Lee W.-W., Jang M., Kim H.J., Kim Y.E., Park H.R., Ehm G., Yang H.-J., Yun J.Y. (2020). Patient selected goals and satisfaction after bilateral subthalamic nucleus deep brain stimulation in Parkinson’s disease. J. Clin. Neurosci..

