# Correction: Acevedo et al. Therapeutic Neurostimulation in Obsessive-Compulsive and Related Disorders: A Systematic Review. *Brain Sci.* 2021, *11*, 948

**DOI:** 10.3390/brainsci12040450

**Published:** 2022-03-28

**Authors:** Nicola Acevedo, Peter Bosanac, Toni Pikoos, Susan Rossell, David Castle

**Affiliations:** 1 Centre for Mental Health, Swinburne University of Technology, John Street, Melbourne, VIC 3122, Australia; tpikoos@swin.edu.au (T.P.); srossell@swin.edu.au (S.R.); 2 St Vincent’s Hospital Melbourne, 41 Victoria Parade, Melbourne, VIC 3065, Australia; peter.bosanac@svha.org.au (P.B.); david.castle@camh.ca (D.C.); 3 Department of Psychiatry, University of Melbourne, Melbourne, VIC 3010, Australia; 4 Centre for Addiction and Mental Health, 252 College Street, Toronto, ON M5T 1R7, Canada

The authors wish to correct the following error in this paper [1].

**Text Correction**

There were errors in the original publication. **It was stated that 73 articles were included, comprising 30 investigations for OCD DBS patients, the manuscript has been corrected to 71 articles comprising 28 investigations for OCD DBS patients. Therefore, 47** (**not 45 at stated**) **articles were excluded; 11** (**not 9 as stated**) **were excluded for reporting of the primary outcomes in another article, resulting in 9** (**not 10 RCTs as stated**)**, and a total 153** (**not 181 as stated**) **OCD DBS cases**. The graphical abstract has been updated accordingly, please see Figure 1.

A correction has been made to **3.7 DBS Results**:

One hundred and eighteen articles were screened for eligibility: 71 were included in the final synthesis, comprising 28 investigations for OCD, 42 for TS, and 1 for BDD. Forty-seven articles were excluded due to a lack of standardized assessment of primary symptoms (*n* = 16), reporting of primary outcomes in another article (*n* = 11), lack of pre- to post- operative outcomes (*n* = 5), adjunct therapy having been implemented (*n* = 4), the primary diagnosis not being an OCRD or was unclear (*n* = 4), previous DBS for Parkinson’s (*n* = 1), or presence of comorbid psychosis (*n* = 1). OCD investigations included 9 RCTs (six with an open-label extension), five open-label trials, two follow-up reports, one pilot study, seven case series, and four case reports. TS investigations included three RCTs with open-label extension, five open-label trials, five follow-up reports, one pilot study, three retrospective reports, seven case series, and 17 case reports. BDD investigations included a single case study. The final sample included, 153 OCD patients, 175 TS patients, and one BDD patient.

**As a result of the overlap of cohorts, a retrospective report was removed** (**reference Suetens et al., 2014**)**, and the outcomes relating to the BNST DBS target**.

A correction has been made to **3.7.1 DBS Results for OCD**:

One RCT implanted two targets (four electrodes) per patient; DBS of the VC/VS, amSTN, and both targets achieved 53%, 45% and 60% mean improvement, respectively [168]. Two open-label studies compared two targets: NAc DBS led to 12%–23% improvement, where-as BNST DBS led to 24–39% improvement [163,164].

Investigations of NAc DBS included three RCTs, one open-label trial, and three case reports. RCTs led to 13% and 51% symptom improvement [161,165], and long-term treatment (6–12 months) led to 12–33% improvement [161,162,167].

Investigations of ALIC DBS encompassed two RCTs, one trial with a staggered switch on, one long-term follow-up report, and two case reports. Closed-label investigations led to 20% and 43% improvement [153,157]; and long-term (1–9 years) treatment led to 43–67% improvement [153,159,160]. The ALIC was also targeted in the cohort of Mantione et al., (2014) through shifts in targeting, and achieved 43% improvement at 1 year [220].

Investigations into VC/VS DBS involved one RCT, one open-label trial with a long-term follow-up report, and three case reports. The RCT originally implanted the ALIC [153], and implemented a posterior shift in target to the VC/VS. A larger cohort from the same site as Nuttin (2003) achieved 42% mean improvement from closed-label treatment, and at three-year follow up, 39% symptom improvement was maintained [156].

Investigations of amSTN DBS involved a multisite RCT that resulted in 25% median improvement, and 51% mean improvement was reached at four-year follow up [158].

DBS of the ITP, slMFB, and thalamus were also targeted for OCD, 52%, 42%, and 9% mean change was respectively achieved per target [166,173,175].

**The manuscript stated that the RoB assessment for OCD DBS articles rated 47 as low risk, 17 as medium risk and nine as high risk. The manuscript has been revised to include 46 as low risk, and 16 as medium risk. The quality assessment for OCD DBS articles rated 46 as moderate, the manuscript has been revised to include 44 as moderate. The manuscript reported that 18** (**9.9%**) **OCD patients had their devices switched off, the proportion of patients has been revised to 11.7%. Lastly, it was reported that comorbidities in OCD DBS patients were not reported in 72 cases, this has been amended to include 44 cases**.

A correction has been made to **3.8 DBS Discussion**:

The RoB assessment rated 46 articles as low risk, 16 as medium risk, and nine as high risk (S2). The quality assessment rated 19 articles as good, 44 as moderate and eight as poor (S3). Only 11 out of 71 articles were RCTs, and 35 were case reports, which meant a randomized control aspect and group level analysis was not present in almost half of the patients included here. Furthermore, only half (36) of the articles reported on more than one time-point, which limits interpretations regarding the duration and pattern of response. Within the bias assessment, there were multiple deviations from the intended protocol, including DBS explants or switch off, and closed-label conditions ending early. It was reported that 18 (11.7%) OCD patients and 12 (6.8%) TS patients had their devices switched off or explanted due to limited/no efficacy or even worsening in some instances; a further three (1.7%) TS patients underwent repositioning. Also, five RCTs had patients that ended the closed-label phase early. It is possible that not all cases of device switch off, explant, or repositioning were captured.

Adverse events included transient psychiatric symptoms, particularly hypomania, increased anxiety, deterioration of mood and suicidal thoughts, which were generally resolved with programming adjustments. There were seven suicide attempts, and one completed suicide [157]. Battery depletion was rarely reported on but seemed to occur between 5–22 months in OCD cohorts [153,154,170] and was reported to occur at 24-months for one TS patient [210].

There was large heterogeneity in protocols, and no comparable protocols were identified, making a meta-analysis not possible. To elaborate, surgical procedure, target trajectory, programming method, stimulation location, comorbidities, follow-up duration, and closed-label conditions varied greatly. Comorbidities reported in OCD trials included MDD (*n* = 17), personality disorder (*n* = 4), bipolar (*n* = 3), PD (*n* = 2), GAD (*n* = 1), panic disorder (*n* = 1), BDD (*n* = 1), TS (*n* = 1), yet was most often not reported (*n* = 44). Comorbidities reported in TS trials included OCD (*n* = 37), MDD (*n* = 29), ADHD (*n* = 15), GAD (*n* = 5), dystonia (*n* = 3), panic disorder (*n* = 1), personality disorder (*n* = 1), and was not reported in a further 26 cases.

**It was stated that the extent of DBS programming for two RCTs was not clear, this has been corrected to one RCT**.

A correction has been made to **3.8.8 Optimized Stimulation Parameters**:

Although implementing predefined stimulation parameters during closed-label phases is advantageous for blinding, it likely limits efficacy. Across targets, it was identified that a lack of programming was a major determinant of suboptimal therapy [157,161]. Trials that had an extensive optimization phase in the weeks prior to closed-label conditions all achieved high efficacy [153,156,165,168]. The extent of programming for one RCT was not clear [158].

**Error in Figure/Table**

The corrected Figure 1 appears below.

**Figure 1:**
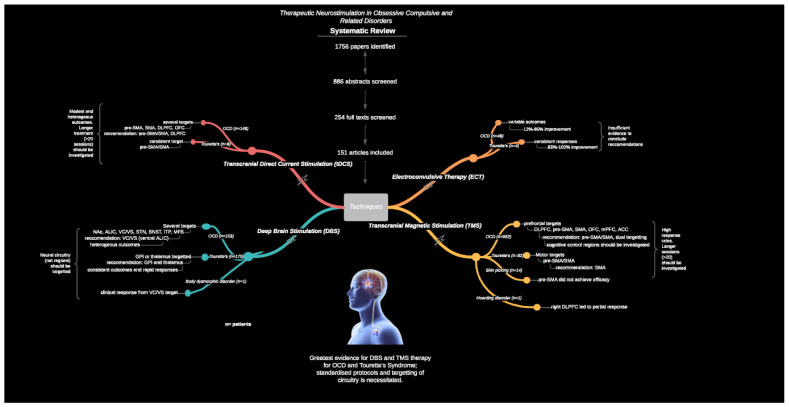
Graphical abstract.

In the original publication, there was a mistake in **Table 12** as published. **The article Suetens et al., (2014) was considered a distinct cohort to the article by Greenberg et al., (2010), and the article by Liebrand et al., (2019) was considered a distinct cohort to the article by Mantione et al., (2014); however, the cases are in fact the same, thus the article by Suetens et al., (2014) and Liebrand et al., (2019) have been removed from Table 12 to avoid duplication of data.** All the reference number have been updated accordingly.

The corrected **Table 12** appears below.

**Table 12:** Summary results of deep brain stimulation for obsessive compulsive disorder.

Study (Country)	N (m:f)	Study Design	Baseline YBOCS	Rx	Stimulation Parameters			YBOCS Outcomes % Change from Pre-Treatment			Comments/Conclusions
					Target (span of trajectory if reported)	Pulse width, Frequency	Stimulation intensity, and configuration (n)	Post treatment (≤6 months, or phase 1)	Follow up (>6 months, or phase 2)	Responders (Criterion, if reported)	
Gabriels et al., 2003 (Belgium) [152]	3 (1;2)	Case series	P1: 38 P2: 33 P3: 30	✓	ALIC	✗	9–10.5 V ✗	✗	12 months: P2: ~27% P3: ~46% + 32 months: P2: ~45% + P3: ~73% +	12 months: 33.3% 32 months: 66.6% (35%)	12 months of ALIC DBS led to response in one patient, partial response in another, and the other had DBS explanted. At 32 months of treatment, efficacy increased and 2 reached response.
Nuttin et al., 2003 (Belgium) [153]	4(✗)	Phase 1: RCT, cross over design Phase 2: OL trial	35 ± 4	✓	ALIC (E0 in NAc)	210/450 µs 100 Hz	4–10.5 V Multipolar (4) Bipolar (1)	A: 43.4% + S: 7.7%	21 months: 56% +	Phase 1: A: 75% S: 0% Phase 2: ✗ (35%)	3 months of closed label ALIC DBS achieved a mean improvement of 43%, and response in 3/4 patients compared to 7.7% improvement and no responders in sham. 2 patients reached phase 2 and improved by 56% at 21 month FU.
Greenberg et al., 2006 (USA) [154]	10(6;4)	OL trial	34.6 ± 0.6		VC/VS	90–210 µs 100–130 Hz	8–17 mA Monopolar (4) Bipolar (6) Unilateral (2)	27.7%	36 months: 35.5% +,*	50% full 75% partial (35% full, 25% partial)	3 months of ALIC DBS led to a mean improvement of 28% and 36 months led to 36% improvement. 20% achieved response at 6 months (2/10), and 50% (4/8) at 36 months.
Greenberg et al., 2010 (Belgium, USA) [155] Long-term FU of Gabriels (2003), Nuttin (2003) and Greenberg (2006) cohorts 26(14;12) Multi-site OL follow up			34 ± 0.5	✓	VC/VS (E0 in Nac)	✗ 100–130 Hz	≤10.5 V ✗	38.2% +,*	36 months: 38.5% +,*	1 month: 28% Last FU: 61.5% (35%)	3 months of VC/VS DBS led to mean improvement of 38%, and no further change at 36 months. 12 patients reached 36 month FU, all were included in the last FU (average 34 months), in which 62% reached response. Depression, anxiety and global functioning significantly improved by 53%, 50% and 69%, respectively, at last FU. CBT was resumed or initiated after 6–12 months. Outcomes of this cohort led to FDA and CE approval or ALIC DBS for TR-OCD.
Luyten et al., 2016 (Belgium, USA) [156] RCT and long-term follow up of Nuttin (2003), Gabriels (2003), Greenberg (2006; 2010) cohorts 24(12;12) Phase 1: Multi-site RCT, cross over design Phase 2: OL follow up			35 ^	✓	ALIC (6), BNST (15) ALIC + BNST (3)	90–450 µs 85–130 Hz	3–10.5 V Multipolar (5) Monopolar (4) Bipolar (8)	A: 42% #,*,+ S: 11% *	48 months: BNST: 50% + ALIC: 22% ALL: 66% +,*	Phase 1: A: 70% S: 26% Phase 2: BNST: 80% ALIC: 16.6% ALIC + BNST: 100% (35%)	3 months of closed label ALIC-BNST DBS (*n* = 17) led to 42% improvement compared to 11% in sham. 18 patients reached the 4-year FU, in which 66% improvement occurred. The optimised target shifted posterior with E0 in the BNST. BNST DBS led to an average of 50% improvement, compared to 22% from ALIC DBS, and 66% from both BNST and ALIC DBS. Anxiety, depression and global functioning improved by 45%, 49%, and 86%, respectively at last FU (54–171 months).
Abelson et al., 2005 (France) [157]	4 (2;2)	Phase 1: RCT, cross over design Phase 2: OL	32.75 ± 5.8	✓	ALIC (E0 in NAc)	60/210 μs 130/150 Hz	4–10.5 V Monopolar (1) Bipolar (3)	A: 19.8% S: 10.5%	Phase 2: 30.2%	Phase 1: A: 25% S: 0% Phase 2: 50% (35%)	Average improvement from two 3-week cycles of ALIC DBS was 20% compared to 11% from sham. The best outcome was reported in phase 2 (4–23 months), individually these were 0% (device explanted), 44% (committed suicide), 73%, and 4%. 2 reached response in phase 2.
Mallet et al., 2008 (France) [158]	16 (9;7)	Multi-site RCT, cross over design	On-off: 30–28 ^ Off-on: 28–31 ^	14/16	amSTN	60 µs 130 Hz	2.0 ± 0.8 V Monopolar (14) Bipolar (1) Mono- and bipolar (1) Unilateral (1)	A: 25.4% #,+ S: 4.1%	✗	A: 75% S: 37.5% (25%)	3 months of closed label amSTN DBS led to median improvement of 25% compared to 4% from sham. Global functioning (but not depression and anxiety) significantly improved in active compared to sham.
Mallet et al., 2019 (France) [13] Long-term FU of Mallet (2009) cohort 14 (6;8) OL follow up			32.4 ± 3.6	✗	amSTN	60 µs 130 Hz	1.2–4 V Monpolar (all)	✗	16 months: 35.4% + 48 months: 51.2% +	48 months: 75% full 92% partial (35% full, 25% partial)	16 and 48 months of amSTN led to mean improvement of 35% and 52%, respectively. Depression and anxiety improved by 53% and 61%, respectively at 4 years. 2 withdrew from the previous report.
Goodman et al., 2010 (USA) [159]	6 (2;4)	Phase 1: Pilot trial, staggered switch on (30 or 60 days post-op) Phase 2: OL	33.2 ± 2.1	✓	ALIC (E0 in VC/VS)	90–210 µs 130/135 Hz	2.5–8.5 V Monopolar (6)	Phase 1: ✗	12 months: 52.8% +,*	Phase 1: 50% Phase 2: 66.6% (35%)	2 or 3 months of ALIC DBS led to response in 3/6 patients (values not reported). At 12 months, mean improvement was 53%, which was not affected by staggered switch on. 2 remained as severe on the CGI, but requested DBS be maintained due to subjective relief of anxiety, depression and tic symptoms.
Fayad et al., 2016 (USA) [160] Long-term follow of Goodman (2010) cohort 6 (2;4) OL follow up			✗	5/6	VC/VS	150–210 µs 130/135 Hz	4–8.5 V Multipolar (2) Monopolar (1) Bipolar (1)	✗	✗	Last FU: 66.6% (35%)	6–9 years of VC/VS DBS led to response in the same 4 patients that achieved response from 12 months of treatment. 1 patient reached partial response of 26% improvement, and the other patient had the device switched off.
Huff et al., 2010 (Germany) [161]	10 (6;4)	Phase 1: RCT, cross over design Phase 2: OL	32.2 ± 4	✓	NAc (E2,3 in ALIC)	90 µs 145 Hz	4.5 V Multipolar (all)	A: 13.3% * S: 3.4%	6 months: 21.1%*	12 months: 10% full 50% partial (35% full, 25% partial)	3 months of closed label, unilateral NAc DBS led to mean improvement of 13.3% compared to 3.4% from sham. Following 3 and 6 months of open label DBS, improvements were 12.4% and 21.1%, respectively. At 12 month FU, 1 patient reached full response.
Mantione et al., 2014 (Netherlands) [162]	16 (9;7)	Phase 1: OL trial, then CBT added Phase 2: RCT, cross over design	33.7 ± 3.6	12/16	NAc (E3 in ALIC)	90 μs 130 Hz	Up to 5 V ✗	Phase 1: 24.6% * Phase 1, CBT: 46% +,*	Phase 2: A: 1.9% (deterioration) S: 44.9% (deterioration) 21 months: 52% +	Phase 1: 37.5% Phase 1, CBT: 56% (35%)	8 months of open label NAc DBS led to 25% improvement. A subsequent 24-week cycle with adjunct CBT led to a further significant improvement, reaching 46% change from pre-op, yet no significant change in depression or anxiety. The subsequent 4 week closed label phase (with CBT) led to deterioration of 1.9% from active and 44.9% from and sham. At 21 months post-op, mean improvement for OCD, anxiety and depression scores were 52%, 57%, and 46%, respectively.
Islam et al., 2015 (Italy) [163]	8 (7;1)	OL trial of 2 targets	Nac: 34.6 ± 4.1 BNST: 35.8 ± 2.2	✗	NAc (3) BNST (5)	90/210 µs 130/180 Hz	4.5–5.5 V Monopolar (4) Bipoar (4)	✗	6 months: Nac: 11.6% BNST: 38.5% +	✗	6 months of BNST DBS led to individual improvements of 25%, 10%, 0% in 3 patients, and NAc DBS led to improvements of 27.5%, 55%, 56%, 25%, 29% in 5 patients. Responders are reported from the last FU (6 months–5 years); 1 NAc patient had the device switched off, the other 2 reached 75% and 60% change at 5 years, 1 BNST patient was reported at 5 years with 30% change, the other 4 reached 6 month FU.
Farrand et al., 2018 (Australia) [164]	7 (3;4)	OL trial	32.4 ± 3.8	✓	NAc (3) BNST (3) NAc-left, BNST-right (1)	✗	✗ Monopolar (all)	✗	Last FU: BNST: 24.4% NAc: 23.4% BNST/NAc: 47.1% + All: 27.3% *	Last FU: BNST: 33.3% NAc: 33% BNST/NAc: 100% ALL: 42.8% (35% full)	Long-term (8–54 months) DBS of the BNST, NAc or both led to an average improvement of 24%, 23%, 47%, respectively. Individual change varied between 7–47%. Depression improved by 23% and anxiety deteriorated by 54% on average.
Barcia et al., 2019 (Spain) [165]	7 (3;4)	RCT, cross over design	32.2 ± 5	✓	NAc (E2-3 in caudate)	60 µs 130 Hz	4.5 V ✗	A: 51.3% +,* S: 25% *	✗	A: 85% S: ✗ (35%)	3 months of closed label NAc DBS with the optimal contact, achieved mean improvement of 52% compared to 25% from sham. The non-responder had a partial response of 25% improvement. 1 patient reached 93% improvement after 3 months (YBOCS = 1). Anxiety did not significantly change from any contact.
Lee et al., 2019 (USA) [166]	5 (2;3)	OL pilot study	35 ± 1.9	✓	ITP	90 µs 130 Hz	5–8.5 V Monopolar (all)	✗	12 months: 52% +,* Last FU: 54% +,*	12 months: 100% (35%)	1 year of ITP DBS led to 52% improvement in OC symptoms and response in all 5 patients, and 54% improvement at last FU (duration was not specified). Anxiety symptoms had a significant improvement at 2 year FU (but not 1 year).
Huys et al., 2019 (Germany) [167]	20 (10;10)	OL trial	30.9 ^	✗	NAc (E0,1), ALIC (E2,3)	90–210 µs 120–180 Hz	3–6 V Multipolar (all)	11.5% *	12 months: 33.3% *	12 months: 40% full 70% partial (35% full, 25% partial)	6 and 12 months of NAc-ALIC DBS led to median improvement of 12% and 33%, respectively. A further significant improvement at 6 and 12 months occurred. Anxiety and depressive symptoms did not significantly improve, and no predictors of response were identified.
Tyagi et al., 2019 (UK) [168]	6 (5;1)	Phase 1: RCT, cross over design of 2 targets Phase 2: OL trial; amSTN, VC/VS amSTN + VC/VS DBS (COMB), optimised settings (OPT), OPT + CBT	36.17 ± 0.75	✓	VC/VS (NAc-ALIC) + amSTN	60 µs 130 Hz	amSTN:1.4–2.6 V VC/VS: 5.4–7 V Monopolar (all)	Phase 1: amSTN: 45.2% +,* VC/VS: 52.9% +,*	Phase 2: COMB: 60.1% +,* OPT: 60.3% +,* OPT + CBT: 74.2% +,*	amSTN: 50% VC/VS: 83.3% COMB: 83.3% OPT: 100% OPT + CBT: 100% (35%)	3 months of closed label amSTN and VC/VS DBS led to mean improvement of 45% and 53%, respectively. There was no statistical effect of conditions (amSTN vs. VC/VS, single vs. both targets, COMB vs. OPT + CBT) on OC symptoms, however the optimised stimulation condition, and adjunct CBT had clinical superiority. Depressive symptoms significantly improved from VC/VS DBS and set shifting significantly improved from amSTN DBS.

A, active; CGI, clinical global impression; E, electrode; Hz, Hertz; P, participant; pre-op, pre-operative; SIB, self-injurious behavior; S, sham; V, Volts; µs, microsecond. + = clinically significant change from baseline; * = statistically significant change from baseline; # = statistically significant change compared to control condition; ✓ = criterion applies; ✗ = not reported; ~ = outcomes were not reported and inferred from graphical reporting. ^ Mallet et al., 2008 [158], Luyten et al., 2016 [156], and Huys et al., 2019 [167], reported median scores rather than mean scores.

The authors apologize for any inconvenience caused and state that the scientific conclusions are unaffected. The original publication has also been updated.
