# Supplementary material for: Therapeutic Neurostimulation in Obsessive-Compulsive and Related Disorders: A Systematic Review
Source: Brain Sci. 2021 Jul 19;11(7):948. doi: 10.3390/brainsci11070948 (PMC8307974; doi:10.3390/brainsci11070948)
Supplement: Supplementary file 1 [file brainsci-11-00948-s001.zip › S2 Risk of Bias Table.pdf]

Risk of Bias Criteria

i) **Deviations from the intended protocol:** Was blinding considered effective for participants and assessors? Were there issues in the methodology that suggest concerns with blinding? Was ’intention-to-treat’ analysis implemented? Was ‘per-protocol effect’ adhered to? did the protocol consider effective blinding? Did participants withdraw during treatment? Was a condition ceased early? Was there appropriate analysis to estimate the effect of assignment to conditions? Were deviations from the protocol balanced between groups? Were deviations of the protocol likely to have affected the outcome?

ii) **Missing outcome data:** Were data for the primary outcome available for all, or nearly all participants randomized? Did missing data indicate that results were biased, i.e. do reasons for missing data differ between intervention groups or specifically relate to the condition? Could missing data depend on its true value? Was analysis conducted appropriately in accordance with missing data?

iii) **Selection of reported results:** Was there selective reporting of a particular outcome measurement? Are the results likely selected from multiple analyses or multiple outcome measurements?

<u>Each bias criteria was evaluated as:</u>

Low risk (✓): if present, bias is unlikely to alter the results

Unknown risk (?): bias may be present, but it is not determined if it alters the results

High risk (!): bias may alter the results seriously

<u>The overall risk of bias for each article was assessed as:</u>

Low: low risk of bias for all criteria

Some: bias is present in one criterion, or unknown bias is present in two or more criteria

High: high risk of bias is present in two or more criteria, or some concerns for all criteria

Risk of Bias Assessment

Study	i	Assessment	ii	Assessment	iii	Assessment	Overall assessment
			**ECT Risk of Bias assessment**				
**Maletzky 1994 (USA)**	✓		✓		✓		Low
**Tomruk et al., 2010 ((Turkey)**	✓		✓		✓		Low
**Liu et al., 2014 (China)**	✓		✓		✓		Low
**Manhas et al., 2016 (India)**	✓		✓		✓		Low
**Aggarwal et al., 2019 (India)**	✓		✓		✓		Low
**Das et al., 2019 (India)**	✓		✓		✓		Low
**Morais et al., 2007 (Brazil)**	✓		✓		✓		Low
**Dehning et al., 2011 (Germany)**	✓		✓		✓		Low
**Rajashree et al., 2014 (India)**	✓		✓		✓		Low
**Guo et al., 2016 (USA)**	✓		✓		✓		Low
			**tDCS risk of bias assessment**				
**Bation et al., 2016 (France)**	✓		✓		✓		Low
**Dinn et al., 2016 (Turkey)**	✓		✓		✓		Low
**Klimke et al., 2016 (Germany)**	✓		✓		✓		low
**D'urso et al., 2016b (Italy)**	?	2 switched from the anodal to cathodal condition	✓		!	Mean change per each condition not reported, statistics conducted on grouped conditions only.	Medium

**Najafi et al., 2017 (Iran)**	✓		✓		!	Statistical analysis does not provide meaningful results	Medium
**Bation et al., 2019 (France)**	?	Blinding not assessed	✓		✓		Low
**Godwa et al., 2019 (France)**	?	Group means for secondary outcomes not reported. Blinding not assessed.	✓		✓		Low
**Kumar et al., 2019 (India)**	✓		✓		✓		Low
**Volpato et al., 2013 (Italy)**	?	Blinding not assessed	✓		✓		Low
**D'urso et al., 2016a (Italy)**	✓		✓		✓		Low
**Mondino et al., 2015 (France)**	✓		✓		✓		Low
**Narayanaswamy et al., 2015 (India)**	✓		!	Follow up YBOCS not reported	✓		Medium
**Alizadeh Goradel et al., 2016 (Iran)**	✓		✓		✓		Low
**Hazari et al., 2016 (India)**	✓		✓		✓		Low
**Silva et al., 2016 (Brazil)**	?	Blinding not assessed	✓		✓		Low
**Palm et al., 2017 (Germany)**	✓		✓		✓		Low
**Mrakic-Sposta et al. 2008 (Italy)**	?	Blinding not assessed	✓		✓		Low
**Carvalho et al., 2015 (India)**	✓		✓		✓		Low
**Eapen et al., 2017 (Australia)**	?	Blinding not assessed	✓		!	YGTSS assessment in protocol, but not reported	Medium

TMS bias assessment

**Alonso et al., 2001 (Spain)**	?	Sham- 90 degree tilt method, possible neural effects. Blinding not assessed.	✓		!	Outcomes immediately post treatment not reported.	Medium
**Sachdev et al., 2001 (Australia)**	?	Sham method not reported. Blinding not assessed.	✓		✓		Low
**Mantovani et al., 2006 (Italy)**	!	Comorbid OCD and TS patients (n=2) withdrew after 5 treatments due to lack of efficacy.	✓	Intention to treat analysis.	!	3 month FU conducted, outcomes not reported.	High
**Prasko et al., 2006 (Czech Republic)**	!	Sham- 90 degree tilt method, possible neural effects. Blinding not assessed. 3 drop outs prior to treatment. Baseline YBOCS was significantly higher in active group.	✓		✓		Medium
**Sachdev et al., 2007 (Australia)**	✓	Sham coil, and active coil used. Blinding effective.	✓		!	Only graphical reporting for primary and secondary outcomes.	Medium
**Kang et al., 2009 (Korea)**	!	Sham- 45 degree tilt method, likely neural effects. Blinding was effective. 1 withdrew from active after 5 treatments.	✓		✓		Medium
**Ruffini et al., 2009 (Italy)**	?	Sham- 90 degree tilt method, possible neural effects. Blinding not assessed.	✓		?	Change in secondary outcomes not reported.	Medium
**Badawy et al., 2010 (Egypt)**	?	Sham- tilt method, angle not specified, likely neural effects.	✓		✓		Low

		Blinding not assessed.					
**Mantovani et al., 2010 (USA)**	!	Sham coil, no sensation effects. 3 withdrew before treatment. Blinding not assessed.	✓		✓		Medium
**Sarkhel et al., 2010 (India)**	?	Sham- 45 degree tilt method, likely neural effects. Blinding not assessed. Baseline anxiety scores were significantly different between groups.	✓		✓		Low
**Kumar & Chadda 2011 (India)**	!	1 patient excluded due to manic symptoms after the 3rd session.	✓		?	Standard deviations not reported.	Medium
**Mansur et al., 2011 (Brazil)**	!	Sham- deactivated coil, no sensation effects. Blinding was effective. 1 withdrew from each group, 1 in the active group was lost to FU.	✓		✓		Medium
**Gomes et al., 2012 (Brazil)**	✓	Sham coil, no sensation effects.	✓		✓		Low
**Nauczyciel et al., 2014 (France)**	✓	Sham coil, no sensation effects. Blinding not assessed.	✓		?	Standard deviations not reported.	Low
**Xiaoyan et al., 2014 (China)**	!	Sham method not reported. Blinding not assessed. 2 patients in each group withdrew after 2 and 3 sessions.	✓		✓		Medium
**Elbeh et al., 2015 (Egypt)**	?	Sham- 90 degree tilt method, possible	✓		✓		Low

		neural effects. Blinding not assessed				
**Haghighi et al., 2015 (Iran)**	?	Sham- 45-90 degree tilt method, possible neural effects.	✓	✓		Low
**Modirrousta et al., 2014 (Canada)**	✓		✓	!	Change in secondary outcomes not reported. Follow up YBOCS reported in graphical form only. Claim 100% response rate, but criteria not defined.	Medium
**Dunlop et al., 2016 (Canada)**	!	1 withdrew after 14 sessions due to non-response.	✓	✓		Medium
**Hawken et al., 2016 (Turkey, Bulgaria)**	!	Sham- 90 degree tilt method, possible neural effects. Blinding not assessed. 1 withdrew after first visit, 1 lost to FU. Reporting of dropouts is inconsistent.	✓	!	YBOCS statistics do not provide meaningful results. The conditions with dropouts was not reported.	High
**Pallanti et al., 2016 (Italy)**	✓		✓	!	Mistakes in reporting, the number of responders reported is inconsistent with values reported.	Medium
**Pelissolo et al, 2016 (France)**	!	Sham coil, no sensation effects. Blinding not assessed. 8 drop outs, 3 before treatment (sham), 1 during treatment in	✓	✓		Medium

		each group, 3 lost to FU in active.					
**Seo et al., 2016 (Korea)**	!	Sham coil, no sensation effects. 1 drop out before treatment. Stated that patients completed ≥70% of sessions, no further details provided.	✓		!	Baseline YBOCS scores not reported, % change not obtainable.	High
**Donse et al., 2017 (Netherlnds)**	!	3 did not complete treatment, 2 due to lack of efficacy, 1 unknown.	✓		!	The number of participants in each stimulation protocol was not specified.	High
**Lee et al., 2017 (Korea)**	✓		✓		!	State significant change in YBOCS, yet inconsistent with statistics reported.	Medium
**Arumugham et al., 2018 (India)**	!	Sham coil, no sensation effects. Blinding not assessed. 2 withdrew before treatment, 1 excluded from analysis due to comorbid bipolar and 1 did not follow protocol.	✓		!	Follow up conducted, outcomes not reported.	High
**Carmi et al., 2018 (Israel)**	!	Sham coil, no sensation effects. Blinding was effective. Deterioration in LF group, thus recruitment stopped and omitted from analysis. 1 withdrew in sham due to conflicting schedule, 2 in active due to	✓		!	YBOCS outcomes presented in graphical form only.	High

		'inconvenience with treatment'.					
**Kumar et al., 2018 (India)**	✓		✓		✓		Low
**Carmi et al., 2019 (US, Israel, Canada)**	!	Sham coil, no sensation effects. Blinding was effective. 2 withdrew from sham during treatment and 5 from active, 3 lost to FU.	✓		✓		Medium
**Harika-Germaneau et al., 2019 (France)**	!	Sham coil, no sensation effects. Blinding not assessed. 2 withdrew before treatment.	✓		✓		Medium
**Singh et al., 2019 (India)**	✓		✓		✓		Low
**Chae et al., 2004 (US)**	?	Sham- 45 degree tilt method, possible neural effects.	✓		✓		Low
**Orth et al., 2005 (UK)**	✓		✓		✓		Low
**Kwon et al., 2011 (South Korea)**	✓		✓		✓		Low
**Le et al., 2013 (China)**	✓		✓		✓		Low
**Wu et al., 2014 (USA)**	!	Sham method not reported. Blinding not assessed.	✓		✓		Medium
**Landeros-Weisenberger et al., 2015 (USA)**	?	Sham coil, no sensation effects. 1 withdrew from each group due to time commitment.	✓	Intention to treat analysis (last outcome carried forward).	✓		Low

		Blinding was effective.					
**Bloch et al., 2016 (Israel)**	!	2 withdrew after 7 and 13 sessions.	✓	Intention to treat analysis.	?	Some secondary outcomes not reported.	Medium
**Aydin et al., 2019 (Turkey)**	!	Sham- 45 degree tilt method, likely neural effects. Blinding not assessed. 1 drop out in sham, 2 lost to FU in active (responders).	✓		!	Incomplete reporting of outcomes, including 1 in sham that deteriorated.	High
**Mantovani et al., 2007 (USA)**	✓		✓		✓		Low
**Talaei et al., 2009 (Iran)**	✓		✓		!	Outcomes not reported following a specific stimulation protocol that led to deterioration.	Medium
**Mantovani et al., 2010 (USA)**	✓		✓		✓		Low
**Wu et al., 2010 (UK)**	✓		✓		✓		Low
**Volpato et al., 2013 (Italy)**	?	Sham coil, no sensation effects. Blinding not assessed.	✓		✓		Low
**Salatino et al., 2014 (Italy)**	!	The patient dropped out after 2 sessions.	✓		✓		Medium
**Diefenbach et al. 2015 (USA)**	✓		✓		✓		Low
**Winkelbeiner et al., 2018 (Switzerland)**	✓		✓		?	Raw scores of outcomes not reported, only	Low

						stated as >25% change.	
**Kar et al., 2019 (India)**	✓		✓		!	Outcomes not reported at follow up.	Medium
			**DBS risk of bias assessment**				
**Nuttin et al., 2003 (Belgium)**	!	Blinding effective. 2/6 not included in RCT phase. 1 explanted in OL phase.	?	Incomplete data for 1 in OL phase.	✓		Medium
**Abelson et al., 2005 (USA)**	!	1 explant, 1 suicide.	✓		✓		Medium
**Greenberg et al., 2006 (USA)**	✓		✓		✓		Low
**Mallet et al., 2008 (France)**	?	24/33 active contacts were within the target, 4 electrodes not placed within the target.	✓		✓		Low
**Goodman et al., 2010 (USA)**	✓		✓		!	Primary outcome during the closed label phase not reported or analysed, graphically reported only.	Medium
**Greenberg et al., 2010 (USA, Belgium)**	✓	Target shifted across implants	✓		✓		Low
**Huff et al, 2010 (Germany)**	✓		✓		✓		Low
**Mantione et al., 2014 (Netherlands)**	✓		✓		✓		Low
**Suetens et al., 2014 (Belgium)**	?	Sham condition ended early for some, reasons not specified.	✓		!	Duration of intervention, and primary outcomes for sham condition not reported.	Medium

**Islam et al., 2015 (Italy)**	✓		!	Errors and inconsistencies in reporting.	!	Primary outcome not reported, graphical form only. Significant change reported, yet statistics not.	High
**Fayad et al., 2016 (USA)**	!	1 patient had DBS switched off, reasoning not reported.	!	1 lost to FU	!	Primary outcome not reported, graphical form only.	High
**Luyten et al., 2016 (Belgium)**	!	14 ended the sham phase early, 3 ended the active phase early. 6 had DBS switched off or explanted at long term FU.	!	17/24 completed closed label phase, reasons not reported. 6 were not receiving DBS at long term FU.	✓		High
**Farrand et al., 2018 (Australia)**	✓		✓		✓		Low
**Barcia et al., 2019 (Spain)**	?	Wash-out implemented during closed label phase.	✓		✓		Low
**Lee et al., 2019 (USA)**	!	1 patient had DBS explanted at 21 months.	✓		!	Short-term follow up scores not reported.	High
**Huys et al., 2019 (Germany)**	✓		✓		✓		Low
**Mallet et al., 2019 (France)**	!	2 had infection and explant (1 before blinding), 2 switched off (10, 22 months), 1 explanted (20 months).	✓		✓		Medium
**Tyagi et al., 2019 (UK)**	?	1 patient ended a condition early due to worsening and switched targets.	✓		✓		Low

**Liebrand et al., 2019 (Netherlands)**	✓		✓	✓		Low
**Gabriels et al., 2003 (Belgium)**	!	1 patient explanted at 15 months.	✓	!	Primary outcome scores not reported, graphical form only.	High
**Franzini et al., 2010 (Italy)**	✓		✓	✓		Low
**Grant et al., 2011 (USA)**	✓		✓	✓		Low
**Roh et al., 2012 (South Korea)**	✓		✓	✓		Low
**Coenen et al., 2014 (Germany)**	✓		✓	?	Some secondary outcomes not reported	Low
**2Tsai et al., 2014 (China)**	?	1 had allergy to battery at 16 months	✓	✓		Low
**Maarouf et al., 2016 (Germany)**	!	2 had DBS explanted, 1 had DBS switched off.	✓	!	Primary outcome not reported for 1 patient.	High
**Chang et al., 2017 (China)**	?	Device explanted (and re-implanted) at 12 months due to skin picking and anxiety.	✓	✓		Low
**Choudhury et al., 2017 (USA)**	✓		✓	✓		Low
**Gupta et al., 2019 (India)**	✓		✓	✓		Low
**Maciunas et al., 2007 (USA)**	✓		✓	✓		Low
**Servello et al., 2008 (UK)**	✓		✓	✓		Low
**Porta et al., 2009 (UK)**	!	3 had DBS switched off, 1 required additional leads within the GPi.	✓	✓		Medium

**Ackermans et al., 2011 (Netherlands)**	!	Dropouts prior to randomization, specifics not reported. Conditions ended early for 6, reasons not reported.	✓		!	Inconsistent reporting in primary outcome.	High
**Martinez-Fernandez et al., 2011 (USA)**	?	1 had lead re-positioned due to limited efficacy. 1 had re-implant due to infection.	✓		✓		Low
**Cannon et al., 2012 (Australia)**	!	3 had hardware malfunction, 1 discontinued DBS at 3 months due to worsening.	✓		!	Outcomes not reported at final FU.	High
**Porta et al., 2012 (Italy)**	!	2 had DBS switched off and remained in FU, 1 deceased from unrelated cause.	✓		✓		Medium
**Motlagh et al., 2013 (USA)**	!	2 had DBS explanted at 3 years, due to infection and lack of efficacy. 1 had additional leads placed in the GPi.	✓		✓		Medium
**Okun et al., 2013 (US)**	✓		✓		✓		Low
**Sachdev et al., 2014 (Australia)**	!	1 had device malfunction and relapse of substance abuse. 1 had DBS switched off at 3 months due to worsening.	?	Missing data for 1 participant.	✓		Medium
**Zhang et al., 2014 (China)**	!	1 had explant at 1 week. 3 had device explanted, 2 due to resumed normality, 1 due to side effects.	!	2 lost to FU at 18 months.	✓		High

**Kefalopoulou et al., 2015 (UK)**	!	2 withdrew before randomisation/ switch on. 1 withdrew from closed label phase from increased anxiety. 2 had programming and medication adjustments during closed label phase.	?	2 incomplete assessments due to fatigue.	✓		Medium
**Huys et al., 2016 (Germany)**	✓		✓		✓		Low
**Rossi et al., 2016 (USA)**	!	1 lost to FU at 18 months.	✓		✓		Medium
**Testini et al., 2016 (USA)**	?	1 trialled DBS switched off due to lack of efficacy.	!	1 lost to FU. YGTSS was not administered for every patient at every follow up.	✓		Medium
**Welter et al., 2017 (France)**	!	3 withdrew before randomisation; 2 due to infection and device removal, 1 due to alcoholism. 1 had misplaced leads and underwent re-implant.<br><br>Post-op/ switch on outcomes used as baseline as opposed to pre-op outcomes.	✓		✓	.	Medium
**Azimi et al., 2018 (Iran)**	✓		✓		✓		Low
**Brito et al., 2019 (Brazil)**	✓		✓		✓		Low
**Welter et al., 2019 (France)**	!	5 lost to FU, 4 at 30 months, 1 at 48 months.	✓		✓		Medium
**Diederich et al., 2005 (Australia)**	✓		✓		✓		Low

**Flaherty et al., 2005 (USA)**	✓		✓	✓		Low
**Houeto et al., 2005 (France)**	✓		✓	✓		Low
**Kuhn et al., 2007 (Germany)**	✓		✓	✓		Low
**Shahed et al., 2007 (USA)**	✓		✓	✓		Low
**Shields et al., 2008 (USA)**	?	Leads replaced due to head jerks and malfunction, target changed due to side effects.	✓	✓		Low
**Dehning et al., 2008 (Germany)**	✓		✓	✓		Low
**Welter et al., 2008 (France)**	✓		✓	!	Sham outcomes not reported, graphical form only.	Medium
**Dehning et al., 2011 (Germany)**	!	1 had repositioning at 8 months, and then switch off 5 months later.	✓	✓		Medium
**Pullen et al., 2011 (USA)**	✓		✓	✓		Low
**Rzesnitzek et al., 2011 (USA)**	✓		✓	✓		Low
**Piedimonte et al., 2013 (Argentina)**	✓		✓	✓		Low
**Savica et al, 2012 (USA)**	✓		✓	✓		Low
**Massano et al., 2013 (Portugal)**	✓		✓	✓		Low
**Dong et al., 2014 (China)**	✓		✓	✓		Low
**Huasen et al., 2014 (UK)**	✓		✓	✓		Low
**Nair et al., 2014 (Australia)**	✓		✓	✓		Low
**Patel et al., 2014 (USA)**	?	Hardware failure at 14 months.	✓	✓		Low

**Wojtecki et al., 2016 (Germany)**	✓		✓	✓	Low
**Kano et al., 2018 (Japan)**	✓		✓	✓	Low
**Kakusa et al., 2019 (USA)**	✓		✓	✓	Low
**Rossi et al., 2019 (Argentina)**	?	Unilateral lead failure at 14 months, no worsening of symptoms	✓	✓	Low
**Zhu et al., 2019 (China)**	!	1 withdrew due to lack of clinical efficacy	✓	✓	Medium
**Burdick et al., 2010 (USA)**	✓		✓	✓	Low
**Baldermann et al., 2016 (Germany)**	✓		✓	✓	Low
