# Supplementary material for: Therapeutic Neurostimulation in Obsessive-Compulsive and Related Disorders: A Systematic Review
Source: Brain Sci. 2021 Jul 19;11(7):948. doi: 10.3390/brainsci11070948 (PMC8307974; doi:10.3390/brainsci11070948)
Supplement: Supplementary file 1 [file brainsci-11-00948-s001.zip › S3 Quality assessment table.pdf]

Quality criteria

i. Appropriate baseline clinical characteristics reported

0= no baseline data reported

1= one or more baseline clinical characteristics missing

2= all clinical characteristics reported: diagnostic method, disease severity, treatment resistance, comorbidities, and symptom profile and disease duration for case report/series.

ii. Prospective and/ or cohort study

0= retrospective case report/series

1= prospective case report/series or retrospective cohort study (n>5)

2= prospective cohort (n≥5) study

iii. Randomised control trial

0= no randomization, blinding or control condition

1= single blinded sham controlled trial, or double blinded randomized trial

2= double blinded, sham controlled randomized trial

iv. Stimulation parameters reported

0= no stimulation parameters reported

1= some stimulation parameters missing

2= all stimulation parameters reported, and frequency of treatment sessions

v. Clinically significant responders reported

0= responders not reported

1= responders not reported in a treatment condition, or at follow-up (if applicable)

2= responders reported in all conditions, and at follow-up (if applicable)

vi. Follow-up assessment of primary outcome

0= follow-up (≥2 weeks) not conducted, 1 time point reported for DBS articles.

1= follow-up outcomes (≥2 weeks) reported, 2 time points reported for DBS articles.

The overall quality assessment per article was assessed as:

Good: 8-11

Moderate: 4-7

Poor: 0-3

Quality assessment

Study	i	ii	iii	iv	v	vi	Overall rating
**ECT Quality assessment**							
Maletzky 1994 (USA)	✓✓	✓	✗	✓	✗	✓	5- moderate
Tomruk et al., 2010 ((Turkey)	✓✓	✓	✗	✗	✗	✗	3- poor
Liu et al., 2014 (China)	✓	✓	✗	✓	✗	✗	3- poor
Manhas et al., 2016 (India)	✓	✓	✗	✓	✓	✓	5- moderate
Aggarwal et al., 2019 (India)	✓	✓	✗	✓	✗	✗	3- poor
Das et al., 2019 (India)	✓✓	✓	✗	✓	✗	✗	4-mderate
Morais et al., 2007 (Brazil)	✓✓	✓	✗	✓✓	✗	✗	5-moderate
Dehning et al., 2011 (Germany)	✓	✓	✗	✓✓	✗	✗	4-moderate
Rajashree et al., 2014 (India)	✓✓	✓	✗	✓	✗	✗	4-moderate
Guo et al., 2016 (USA)	✓✓	✓	✗	✓✓	✗	✗	5-moderate
**tDCS quality assessment**							
Bation et al., 2016 (France)	✓✓	✓✓	✗	✓✓	✓	✓	8-good
Dinn et al., 2016 (Turkey)	✓	✓✓	✗	✓✓	✗	✓	6-moderate
Klimke et al., 2016 (Germany)	✓	✓✓	✗	✓✓	✓✓	✗	7-moderate
D'urso et al., 2016b (Italy)	✓✓	✓✓	✓✓	✓✓	✗	✗	8-good
Najafi et al., 2017 (Iran)	✓✓	✓✓	✗	✓✓	✗	✗	6-moderate
Bation et al., 2019 (France)	✓✓	✓✓	✓✓	✓✓	✓✓	✓	11-good
Godwa et al., 2019 (France)	✓✓	✓✓	✓✓	✓✓	✓✓	✗	10-good
Kumar et al., 2019 (India)	✓✓	✓✓	✗	✓✓	✓✓	✓	9-good
Volpato et al., 2013 (Italy)	✓	✓	✓	✓✓	✗	✗	5-moderate
D'urso et al., 2016a (Italy)	✓✓	✓	✗	✓✓	✗	✓	6-moderate
Mondino et al., 2015 (France)	✓✓	✓	✗	✓✓	✗	✓	6-moderate
Narayanaswamy et al., 2015 (India)	✓✓	✓	✗	✓✓	✗	✗	5-moderate

**Alizadeh Goradel et al., 2016 (Iran)**	✓✓	✓	✗	✓✓	✗	✓	6-moderate
**Hazari et al., 2016 (India)**	✓	✓	✗	✓	✗	✗	3-poor
**Silva et al., 2016 (Brazil)**	✓✓	✓	✓✓	✓✓	✗	✓	8-good
**Palm et al., 2017 (Germany)**	✓	✓	✗	✓✓	✗	✗	4-moderate
**Mrakic-Sposta et al. 2008 (Italy)**	✓	✓	✓	✓✓	✗	✗	5-moderate
**Carvalho et al., 2015 (India)**	✓	✓	✗	✓✓	✗	✓	5-moderate
**Eapen et al., 2017 (Australia)**	✓	✓	✓	✓	✗	✓	5-moderate
**Behler et al., 2018 (Germany)**	✓	✓	✗	✓✓	✗	✗	4-moderate
**TMS quality assessment**							
**Alonso et al., 2001 (Spain)**	✓	✓✓	✓✓	✓✓	✓✓	✓	10- good
**Sachdev et al., 2001 (Australia)**	✓✓	✓✓	✓✓	✓✓	✓✓	✓	11- good
**Mantovani et al., 2006 (Italy)**	✓	✓✓	✗	✓✓	✓✓	✓	8- good
**Prasko et al., 2006 (Czech Republic)**	✓✓	✓✓	✓✓	✓✓	✗	✓	9- good
**Sachdev et al., 2007 (Australia)**	✓✓	✓✓	✓✓	✓✓	✓✓	✗	10- good
**Kang et al., 2009 (Korea)**	✓✓	✓✓	✓✓	✓✓	✓✓	✓	11- good
**Ruffini et al., 2009 (Italy)**	✓	✓✓	✓✓	✓✓	✓✓	✓	10- good
**Badawy et al., 2010 (Egypt)**	✓	✓✓	✓✓	✓	✓✓	✗	8- good
**Mantovani et al., 2010 (USA)**	✓✓	✓✓	✓✓	✓✓	✓✓	✗	10- good
**Sarkhel et al., 2010 (India)**	✓	✓✓	✓	✓✓	✗	✓	7- moderate
**Kumar & Chadda 2011 (India)**	✓	✓✓	✗	✓✓	✗	✗	5- moderate
**Mansur et al., 2011 (Brazil)**	✓	✓✓	✓✓	✓✓	✓✓	✓	10- good
**Gomes et al., 2012 (Brazil)**	✓	✓✓	✓✓	✓✓	✓✓	✓	10- good
**Nauczyciel et al., 2014 (France)**	✓✓	✓✓	✓✓	✓✓	✗	✓	9- good
**Xiaoyan et al., 2014 (China)**	✓	✓✓	✓✓	✓✓	✓✓	✗	9- good
**Elbeh et al., 2015 (Egypt)**	✓	✓✓	✓✓	✓✓	✗	✓	8- good

Haghighi et al., 2015 (Iran)	✓	✓✓	✓	✓✓	✓✓	✗	8- good
Modirrousta et al., 2014 (Canada)	✓✓	✓✓	✗	✓✓	✗	✗	6- moderate
Dunlop et al., 2016 (Canada)	✓✓	✓✓	✗	✓✓	✓✓	✗	8- good
Hawken et al., 2016 (Turkey, Bulgaria)	✓✓	✓✓	✓✓	✓✓	✓✓	✓	11- good
Pallanti et al., 2016 (Italy)	✓✓	✓✓	✗	✓✓	✓✓	✗	8- good
Pelissolo et al, 2016 (France)	✓✓	✓✓	✓✓	✓✓	✓✓	✗	10- good
Seo et al., 2016 (Korea)	✓✓	✓✓	✓✓	✓✓	✓✓	✗	10- good
Donse et al., 2017 (Netherlnds)	✓✓	✓✓	✗	✓✓	✓✓	✗	8- good
Lee et al., 2017 (Korea)	✓✓	✓✓	✗	✓✓	✗	✗	6- moderate
Arumugham et al., 2018 (India)	✓✓	✓✓	✓✓	✓✓	✓✓	✗	10- good
Carmi et al., 2018 (Israel)	✓✓	✓✓	✓✓	✓✓	✓✓	✗	10- moderate
Kumar et al., 2018 (India)	✓✓	✓	✗	✓✓	✓✓	✓	8- good
Carmi et al., 2019 (US, Israel, Canada)	✓✓	✓✓	✓✓	✓✓	✓✓	✓	11- good
Harika-Germaneau et al., 2019 (France)	✓✓	✓✓	✓✓	✓✓	✓✓	✓	11- good
Singh et al, 2019 (India)	✓✓	✓	✗	✓✓	✓✓	✗	7-moderate
Chae et al., 2004 (US)	✓	✓	✗	✓✓	✓	✗	5- moderate
Orth et al., 2005 (UK)	✓	✓	✓	✓✓	✗	✗	5- moderate
Kwon et al., 2011 (South Korea)	✓	✓✓	✗	✓✓	✗	✓	6- moderate
Le et al., 2013 (China)	✓	✓✓	✗	✓✓	✗	✓	6- moderate
Wu et al., 2014 (USA)	✓	✓✓	✓✓	✓✓	✓✓	✗	9- good
Landeros-Weisenberger et al., 2015 (USA)	✓✓	✓✓	✓✓	✓✓	✓✓	✗	10- good
Bloch et al., 2016 (Israel)	✓✓	✓✓	✗	✓✓	✗	✗	6- moderate
Aydin et al., 2019 (Turkey)	✓	✓✓	✓✓	✓✓	✓✓	✗	9- good
Mantovani et al., 2007 (USA)	✓	✓	✗	✓✓	✗	✓	5- moderate
Talaei et al., 2009 (Iran)	✓	✓	✗	✓✓	✗	✗	4- moderate

Mantovani et al., 2010 (USA)	✓	✓	✗	✓✓	✓✓	✗	6- moderate
Wu et al., 2010 (UK)	✓	✓	✓✓	✓✓	✗	✗	6- moderate
Volpato et al., 2013 (Italy)	✓	✓	✗	✓✓	✗	✗	4- moderate
Salatino et al., 2014 (Italy)	✓	✓	✗	✓✓	✗	✗	4- moderate
Diefenbach et al. 2015 (USA)	✓	✓	✗	✓✓	✗	✓	5- moderate
Winkelbeiner et al., 2018 (Switzerland)	✓	✗	✗	✓✓	✗	✗	3- poor
Kar et al., 2019 (India)	✓	✓	✗	✓✓	✓✓	✗	6- moderate
**DBS quality assessment**							
Nuttin et al., 2003 (Belgium)	✓	✓✓	✓✓	✓✓	✓	✓	9- good
Abelson et al., 2005 (USA)	✓	✓	✓✓	✓✓	✓✓	✓	9- good
Greenberg et al., 2006 (USA)	✓✓	✓✓	✗	✓✓	✓✓	✓	9- good
Mallet et al., 2008 (France)	✓✓	✓✓	✓✓	✓✓	✓✓	✗	10- good
Goodman et al., 2010 (USA)	✓✓	✓✓	✓✓	✓✓	✓✓	✗	10- good
Greenberg et al., 2010 (USA, Belgium)	✓✓	✓✓	✗	✓	✓✓	✓	8- good
Huff et al, 2010 (Germany)	✓✓	✓✓	✓✓	✓✓	✓	✓	10- good
Mantione et al., 2014 (Netherlands)	✓	✓✓	✓✓	✓	✓✓	✓	9- good
Suetens et al., 2014 (Belgium)	✓	✓✓	✓✓	✗	✓	✗	6- moderate
Islam et al., 2015 (Italy)	✓✓	✓✓	✗	✓✓	✓✓	✓	9- good
Fayad et al., 2016 (USA)	✓	✓✓	✗	✓✓	✓	✓	7- moderate
Luyten et al., 2016 (Belgium)	✓✓	✓✓	✓	✓✓	✓✓	✓	10- good
Farrand et al., 2018 (Australia)	✓✓	✓✓	✗	✗	✓✓	✗	6- moderate
Barcia et al., 2019 (Spain)	✓✓	✓✓	✓✓	✓	✓✓	✓	10- good
Lee et al., 2019 (USA)	✓✓	✓✓	✗	✓✓	✓✓	✓	9- good
Huys et al., 2019 (Germany)	✓	✓✓	✗	✓✓	✓✓	✓	8- good
Mallet et al., 2019 (France)	✓✓	✓✓	✗	✓✓	✓✓	✓	9- good

Tyagi et al., 2019 (UK)	✓✓	✓✓	✓	✓✓	✓✓	✓	10- good
Liebrand et al., 2019 (Netherlands)	✓✓	✓	✗	✓✓	✓✓	✗	7- moderate
Gabriels et al., 2003 (Belgium)	✓✓	✓	✗	✗	✗	✗	3- poor
Franzini et al., 2010 (Italy)	✓✓	✓	✗	✓✓	✗	✗	5- moderate
Grant et al., 2011 (USA)	✓	✓	✗	✗	✗	✗	2- poor
Roh et al., 2012 (South Korea)	✓✓	✓	✗	✓✓	✓✓	✗	7- moderate
Coenen et al., 2014 (Germany)	✓	✓	✗	✓✓	✓✓	✗	6- moderate
Tsai et al., 2014 (China)	✓✓	✓	✗	✓	✗	✗	4- moderate
Maarouf et al., 2016 (Germany)	✓✓	✗	✗	✓✓	✓✓	✗	6- moderate
Chang et al., 2017 (China)	✓✓	✓	✗	✓✓	✗	✓	6- moderate
Choudhury et al., 2017 (USA)	✓✓	✓	✗	✓✓	✗	✓	6- moderate
Gupta et al., 2019 (India)	✓✓	✓	✗	✗	✓✓	✓	6- moderate
Maciunas et al., 2007 (USA)	✓	✓✓	✓✓	✓✓	✓✓	✗	9- moderate
Servello et al., 2008 (UK)	✓✓	✓✓	✗	✓✓	✗	✗	6- moderate
Porta et al., 2009 (UK)	✓	✓✓	✗	✗	✗	✗	3- poor
Ackermans et al., 2011 (Netherlands)	✓✓	✓✓	✓✓	✓✓	✓✓	✓	11- good
Martinez-Fernandez et al., 2011 (USA)	✓	✓✓	✗	✓✓	✗	✗	5- moderate
Cannon et al., 2012 (Australia)	✓	✓✓	✗	✓✓	✓✓	✓	8- good
Porta et al., 2012 (Italy)	✓✓	✓✓	✗	✓✓	✗	✗	6- moderate
Motlagh et al., 2013 (USA)	✓✓	✓✓	✗	✓✓	✓✓	✓	9- moderate
Okun et al., 2013 (USA)	✓✓	✓✓	✓✓	✓✓	✓✓	✗	10- moderate
Sachdev et al., 2014 (Australia)	✓✓	✓✓	✗	✓	✓✓	✗	7- moderate
Zhang et al., 2014 (China)	✓	✓	✗	✓	✓✓	✓	6- moderate
Kefalopoulou et al., 2015 (UK)	✓✓	✓✓	✓✓	✓✓	✓✓	✗	10- good
Huys et al., 2016 (Germany)	✓✓	✓✓	✗	✓✓	✗	✓	7- moderate

**Rossi et al., 2016 (USA)**	✓	✓✓	✗	✓	✓✓	✗	6- moderate
**Testini et al., 2016 (USA)**	✓	✓✓	✗	✓	✓✓	✗	6- moderate
**Welter et al., 2017 (France)**	✓✓	✓✓	✗	✓✓	✓✓	✓	9- good
**Azimi et al., 2018 (Iran)**	✓✓	✓✓	✗	✓✓	✗	✗	6- moderate
**Brito et al., 2019 (Brazil)**	✓	✓✓	✗	✗	✓✓	✗	5- moderate
**Welter et al., 2019 (France)**	✓	✓✓	✗	✗	✓✓	✓	6- moderate
**Diederich et al., 2005 (Australia)**	✓✓	✗	✗	✓✓	✗	✗	4- moderate
**Flaherty et al., 2005 (USA)**	✓	✗	✗	✓✓	✗	✗	3- poor
**Houeto et al., 2005 (France)**	✓✓	✗	✗	✓✓	✗	✓	5- moderate
**Kuhn et al., 2007 (Germany)**	✓	✗	✗	✓✓	✗	✗	3- poor
**Shahed et al., 2007 (USA)**	✓✓	✗	✗	✓✓	✗	✗	4- moderate
**Shields et al., 2008 (USA)**	✓✓	✗	✗	✓	✗	✓	4- moderate
**Dehning et al., 2008 (Germany)**	✓	✗	✗	✓✓	✗	✓	4- moderate
**Welter et al., 2008 (France)**	✓	✗	✗	✓	✗	✗	2- poor
**Dehning et al., 2011 (Germany)**	✓	✗	✗	✓	✓✓	✓	5- moderate
**Pullen et al., 2011 (USA)**	✓	✓	✗	✗	✗	✗	2- poor
**Rzesnitzek et al., 2011 (USA)**	✓✓	✗	✗	✓✓	✗	✗	4- moderate
**Piedimonte et al., 2013 (Argentina)**	✓	✗	✗	✓✓	✓✓	✓	6- moderate
**Savica et al, 2012 (USA)**	✓	✗	✗	✓✓	✓✓	✗	5- moderate
**Massano et al., 2013 (Portugal)**	✓✓	✗	✗	✓✓	✓✓	✓	7- moderate
**Dong et al., 2014 (China)**	✓✓	✗	✗	✓✓	✗	✓	5- moderate
**Huasen et al., 2014 (UK)**	✓✓	✗	✗	✓✓	✗	✗	4- moderate
**Nair et al., 2014 (Australia)**	✓	✓	✗	✓✓	✓✓	✗	6- moderate
**Patel et al., 2014 (USA)**	✓✓	✗	✗	✓✓	✗	✗	4- moderate
**Wojtecki et al., 2016 (Germany)**	✓	✓	✗	✓✓	✗	✓	5- moderate

Kano et al., 2018 (Japan)	✓	✗	✗	✓✓	✗	✓	4- moderate
Kakusa et al., 2019 (USA)	✓✓	✗	✗	✓✓	✓✓	✓	7- moderate
Rossi et al., 2019 (Argentina)	✓	✗	✗	✓✓	✗	✓	4- moderate
Zhu et al., 2019 (China)	✓	✗	✗	✓✓	✗	✗	3- poor
Burdick et al., 2010 (USA)	✓✓	✗	✗	✓✓	✗	✓	5- moderate
Baldermann et al., 2016 (Germany)	✓✓	✗	✗	✓✓	✗	✗	4- moderate
