# Supplementary material for: Therapeutic Neurostimulation in Obsessive-Compulsive and Related Disorders: A Systematic Review
Source: Brain Sci. 2021 Jul 19;11(7):948. doi: 10.3390/brainsci11070948 (PMC8307974; doi:10.3390/brainsci11070948)
Supplement: Supplementary file 1 [file brainsci-11-00948-s001.zip › S4 DBS targets.pdf]

The following targets have been investigated for OCRD:

Caudate nucleus regions

- ALIC (anterior limb of the internal capsule): situated between the head of the caudate nucleus and lenticular nucleus. The ALIC is a fiber bundle of myelinated axons that carry PFC/ ACC fibers to the thalamus and brainstem that are involved in motivation, cognitive processing and decision-making.
- VC/VS (ventral capsule/ventral striatum): includes the ventral caudate nucleus and the nucleus accumbens, and the ventral capsule. The VC/VS is targeted for its role in reward, motivation, and decision-making.
- BNST (bed nucleus of stria terminalis): is a structure of the amygdala, located behind the NAc, and is targeted for its role in reward, fear, and anxiety.
- NAc (nucleus accumbens): is the most ventral extension of the striatum, located below the ALIC, between the anterior putamen and head of the caudate nucleus, and is also part of the basal ganglia. The NAc and ventral striatum (VS) may be referred to interchangeably as the confluence between the putamen and caudate. The NAc has a central role in reward circuits.

Basal ganglia regions

- STN (subthalamic nucleus): is part of the basal ganglia, and has three functionally subdivisions of dorsal (motor), ventral (associative) and medial (limbic and associative) regions, which receive topographic innervations from the PFC.
- GPi (globus pallidus internus): The GPi is also part of the basal ganglia, and has three functional areas that are organized topographically to receive prefrontal innervations. The subdivisions include the anteromedial (limbic), posterior (motor and sensorimotor) and the dorsal part is the associative region.
- GPe (external globus pallidus): is lateral to the GPi, and receives inhibition from the caudate and sends inhibitory innervations to the STN. The GPe functions to control conscious and proprioceptive movements.

Other regions

- ITP (inferior thalamic peduncle): is a connection structure between the OFC and thalamus and is targeted for its role in motor and cognitive components of selective attention.
- CM (centromedian) thalamus: is part of the intralaminar nucleus of the thalamus and has reciprocal connections to the basal ganglia. The CM thalamus processes sensory, arousal and executive information that is important for motor planning.
- MFB (medial forebrain bundle): is a neural pathway with fibers passing through the lateral hypothalamus and basal forebrain, and has widespread projections to prefrontal and subcortical regions. The MFB is targeted for its role in reward and pleasure.
