# Supplementary material for: Therapeutic Neurostimulation in Obsessive-Compulsive and Related Disorders: A Systematic Review
Source: Brain Sci. 2021 Jul 19;11(7):948. doi: 10.3390/brainsci11070948 (PMC8307974; doi:10.3390/brainsci11070948)
Supplement: Supplementary file 1 [file brainsci-11-00948-s001.zip › Supplementary results.pdf]

Supplementary descriptive results

tDCS results for OCD

Pre-SMA/SMA

One randomised trial implemented polarity as the randomized aspect without a sham control [1]: if symptoms deteriorated from the randomized condition of anodal (A) or cathodal (C), polarity was switched (A-C or C-A), otherwise the condition was repeated (A-A or C-C). Two patients received A-A and improved by 6.6% on average, two received A-C due to deterioration from A, and then improved by 15% on average. All five patients randomized to cathodal received C-C and had a 20% improvement on average. Cathodal tDCS of the SMA in an open label cohort study led to 17% improvement and 15% response rate [2]. Cathodal tDCS of the pre-SMA led to either a 17% or 0% change in two patients [3].

A RCT of anodal tDCS over the pre-SMA led to improvement of 22% vs 12% from sham and a response rate of 33% vs 9% in sham [4]. An additional five sessions (open label) were provided for non-responders; those who entered from sham (n=11) did not improve further, and those from active (n=7) had a further statistically significant improvement, but there were no additional responders. Anodal tDCS over the pre-SMA and SMA led to individual symptom improvements of 40%, 46.7%, 69.5% [5,6]; one patient deteriorated by 12% and switching polarity to cathodal led to partial response with 29% improvement and 32% improvement at three month follow-up [7].

DLPFC

The open label trial of cathodal tDCS of bilateral DLPFC led to 65% improvement [8]. After five and ten days of treatment, mean improvements were 27% and 51% respectively, with further improvement to 81% at three-month follow-up. A case report showed 9% change from cathodal tDCS over the left DLPFC in a blinded and sham controlled protocol [9]. Anodal stimulation of the left DLPFC and cathodal over the right DLPFC led to 22% improvement in a single patient [10].

OFC

An open label study of five patients used cathodal stimulation of the right OFC and anodalof the left DLPFC. There was an initial 23% improvement in OC symptoms but a 2% deterioration was noted at one month follow-up, yet improvements in depression (31%) remained [11].

Cathodal tDCS of the left OFC assessed in a RCT led to 5% improvement and 2% deterioration in sham, and response in 20% vs 9% in sham [12]. The same montage within an open label study, was associated with a 22% improvement and 26.5% response rate, which increased to 38% at three-month follow-up [13]. Cathodal stimulation of the left OFC and anodal stimulation of the right OFC has been investigated in two case studies, with one patient recording no response immediately following treatment, and a partial response (26%) two weeks later [14]; the other had a 56% improvement in symptoms post-treatment, and 65% two weeks later [15].

tDCS results for TS

Motor targets

tDCS targeting the pre-SMA led to individual improvement of 41% (partial response of 23% was reached after just 5 days of therapy), and efficacy was maintained six-months later [16]. tDCS of the SMA in two patients led to improvements of 34% and 20.5%, which increased to 43% and 29% at three-month follow-up [17]. When tDCS was applied to both the pre-SMA and SMA, YGTSS score improvements of 34%, 13% and 5% occurred in three patients, yet tic counts increased in all patients by up to 200% in one of them [18]. tDCS of the primary motor cortex in two patients within a randomized controlled protocol led to 30% and 11% vs 5% and 5% from sham [19].

TMS results for OCD

DLPFC

Two RCTs targeted bilateral DLPFC, with HF active rTMS being associated with 37% and 27% symptom improvement when administered prior to, and following sham, respectively; sham led to no change, and 3% deterioration when administered prior to and following active stimulation, respectively [20].

Also, alpha guided frequency rTMS of bilateral DLPFC led to improvement of 32% vs 15% with sham; response rates were 36% vs 0% [21]. When laterality of HF rTMS of DLPFC was examined in an RCT, improvements of 26% from left and 37% from right rTMS were achieved with a comparable response rate of 33% [22].

A RCT of LF rTMS targeting left DLPFC showed improvements of 24% vs 16% with sham, yet at two-week follow-up 28% change from baseline was found in both groups [23]. The pre-treatment severity scores were significantly higher in the active group, such that the absolute but not relative change was greater in those patients. A case report achieved response from LF rTMS of the left DLPFC, but the degree of change was not reported [24].

A RCT of HF rTMS of the left DLPFC led to response in 30% vs 25% in sham. All patients received an extra 10 sessions (open label) with 33% of the entire sample achieving response [25]. Badawy and colleagues performed a non-randomised trial with three arms; medication naïve, and medicated (but SSRI resistant) patients both received HF active rTMS targeting the left DLPFC, while medication naïve patients received sham [26]. Response was achieved in 55% of those who received rTMS combined with SSRI treatment, 25% who received rTMS and were un-medicated, and 5% who were delivered sham; mean change was 20%, 8%, and 5% respectively.

Three RCTs administered LF rTMS of the right DLPFC. Response rates following active and sham were 20% vs 12.5% [27], 50% vs 23% [28], and 20 vs 20% when combined with LF rTMS of SMA [29]. Mean symptom improvements were 14% in active, 1% in sham [27]; 8.6% in active, 6.8% in sham [29], or 11 points in active and 4 points in sham [28]. In a single patient, 10 sessions (4 weeks) of continuous TBS

of the right DLPFC led to a 58% improvement, but subsequent intermittent TBS led to no further improvement in OC symptoms [30].

A RCT of HF rTMS of the right DLPFC led to response rates of 31% vs 14% in sham [31]. The same montage in a single blind non-randomised, sham-control trial led to improvements of 9.4% vs 7.3% from sham, which increased to 19.4% and 17.6% at one-month [32].

When frequency of the right DLPFC was examined within a RCT, symptom improvement reached 45% for LF, 27% for HF and 5.6% for sham conditions; at three-month follow-up, respective improvements from baseline were 41%, 10% and 8% [33].

Pre-SMA/SMA

RCTs achieved response rates from active and sham of 22% and 11% [34], 42% and 12% [35], 80% and 8.3% [36], 10% and 20% [37], and 32% and 18% [38]. Mean symptom changes associated with active and sham were 25% and 12% [34], 42% and 16% [35], 40% and 6% [36], 8% and 10% [37], 23% and 16% [38]. Mantovani (2010) showed that an additional 20 rTMS sessions (open label) following 20 closed label active sessions within a subgroup of four patients, achieved an overall symptom improvement of 49% and all four met response criteria, maintained at three-month follow-up.

LF rTMS targeting the SMA was compared to treatment as usual (TAU) in a randomised open label trial, and led to response in 68% vs 24% from TAU, with symptom change of 30% and 19%, respectively [39]. Open label investigations of the SMA reported response in 60% [40], and mean improvement of 34% [41], and 17% [42]. Case studies of pre-SMA stimulation showed improvements of 27%, 35% and 54% [43-45]; and 76% from right SMA stimulation [43].

In an open label trial, LF rTMS study, the SMA was targeted for OCD symptoms and right DLPFC for comorbid MDD (in 22/25 patients), achieving a response rate of 83% with mean symptom improvement of 42% [46].

One RCT investigated continuous TBS of the pre-SMA, with response in 29% vs 36% in sham; symptom improvements were 13% and 17%, respectively [47].

OFC

Left OFC treatment led to response in 25% vs 0% in sham, and symptom improvement of 19% vs 7% in sham [48]. At 10-week follow-up, improvements were 21% and 8% in sham. Right OFC treatment using deep TMS led to improvements of 19% vs 6% in sham, but improvements had abated at one-month follow-up [49]. A retrospective investigation of left OFC showed response in 44%, and mean symptom improvement of 24%, which were maintained at one-month follow-up [50].

A large retrospective report used LF rTMS to target the SMA (n=46) or OFC (n=33), with the choice of target resting with the patient. Across the whole cohort, 27% improvement and 41% response was reached; there was no statistical effect of target, with 39% response from SMA, and 43% response from OFC [51].

Other frontal targets

Randomised controlled investigations of HF deep TMS targeting the medial PFC and ACC led to response in 44% vs 7% in sham [52] and 45% vs 12% in sham [53]. Open label applications of LF rTMS of the medial PFC led to mean symptom improvement of 39% [54] whilst 40% improvement was achieved in a study of HF rTMS of the dorsal medial PFC [55]. There was no effect of LF rTMS of the primary motor cortex in a single patient who received rTMS and tDCS treatment [9].

TMS results for TS

SMA

Response for active and sham conditions within RCTs were 50% vs 50% using TBS [56], and 33% vs 18% with rTMS [57]. In the latter study, an additional 15 sessions (open label) were administered to 7 participants from the active condition, of which 71% responded; and 9 patients from the sham condition, 44% responded [57]. Open label investigations showed improvements of 34%, 30% and 4.6% [58-60]; in the two former studies, efficacy was sustained at three and six months.

Two case series of LF rTMS of the SMA reported mean improvement of 67% in four patients [40], with individual improvements of 36% and 68% [61].The patient with 36% improvement relapsed and required additional treatments for efficacy to be maintained one-month later, and the patient with 68% improvement sustained 57% improvement at four month follow-up. Another patient improved by 18% from two rTMS sessions of the pre-SMA [62].

Motor cortex

A small RCT of five patients [63] applied two LF treatments to the pre-motor cortex; bilateral or left stimulation did not affect OC symptoms (≤2.5% change). Another pilot RCT applied a different protocol each day to assess high and low frequency treatment of the motor cortex, or PFC [64]: high frequency stimulation of the PFC showed superiority, with 29% improvement. However, sham led to 22% improvement.

TMS results for OCRD

One RCT investigated LF rTMS of the pre-SMA for skin picking, symptom improvement of 36% was achieved, but two of the five patients in the active group were lost to follow-up and the remaining three worsened between 70-110% at three-month follow-up [65]. A case study of LF rTMS of right DLPFC achieved a 30% symptom improvement for one patient with hoarding disorder, that maintained at two-month follow-up [66].

DBS results for OCD

Multiple targets within a cohort

Once RCT systematically compared two targets within six patients by implanting leads in the VC/VS and amSTN; the stimulation target was the control rather than a sham condition [67]. DBS of the VC/VS led to 53% improvement and 83% response rate; DBS of the amSTN led to 45% improvement and 50% response rate; and DBS of both the VC/VS and amSTN led to 60% improvement and 83% response rate.

Open label comparison of NAc or BNST DBS within a cohort led to 12% and 39% improvement at six months, respectively [68], and in a different cohort there was 23% and 24% improvement at long term (8-54 months) follow-up [69].

NAc

Randomised controlled investigations led to improvements of 13% vs 3% from sham [70], and 51% vs 25% from sham [71]. The most recent RCT achieved 85% response, yet response from sham was not reported [71]. Outcomes of one RCT are addressed in the Discussion, as adjunct CBT was involved; rather the open label outcomes are reported here.

Across three cohorts, follow-up improvements reached 12% (6-months), 21% (6-months), 25% (8-months) and 33% (12-months) [70,72,73]. Response rates of 40% (70% partial) and 10% (50% partial) were achieved from 12 months of DBS [70,73].

Case reports of three patients led to individual improvements of 69% at 8 months, 34% and 42% at 24 months [74,75]. Another patient cycled through periods of no change and deterioration by 13% across 30 months [76]. No studies reported that patients had DBS explanted or switched off.

ALIC

Closed label treatment led to improvements of 43% vs 8% with sham, response in 75% vs 0% with sham [77], also improvements of 20% vs 11% with sham, response in 25% vs 0% with sham [78]. Staggered switch on, at 30- or 60-days following surgery did not affect 12-month outcomes and led to 50% response [79].

Long-term outcomes showed improvements of 43%, 53% (12-months), 56% (21-months) and response in 58%, 67% (12-months) and 67% (6-9 years) across three cohorts [77,79-81]. When the best outcome was reported in the cohort of Abelson (2005) between 4-23-months, 30% mean change was achieved. Case reports showed individual improvements of 19%, 68% and 79% after 12-months [82,83].

Out of 19 patients, two had their devices explanted at 4 and 15-months following surgery, one had their device switched off (timing not reported), and one patient completed suicide 9-months following surgery [77,78,80].

VC/VS

The multi-site RCT originally targeted the ALIC, and the first few implants are reported in Nuttin (2003) within the ALIC outcomes outlined above. Across implants the target was refined and shifted more posterior, targeting the VC/VS. The closed label phase with these latter implants led to improvements of 42% vs 11% from sham, and response in 70% vs 26% from sham [84]. Other reports of the same cohort as Luyten (2016) showed improvements of 28% (n=10) at three-months, 36% (n=10) and 39% (n=26) at three-years [85,86]. Across the entire cohort, 28% reached response at one-month, and 62% reached response at three-years [86].

Four patients showed mean improvement of 33% at 15-months [87], and in a separate study all four participants achieved response, with a mean improvement of 60% at two-years [88]. An additional patient had 31% improvement at one-year yet developed compulsive picking around the battery eventuating in re-implantation; eight-months later, improvement from initial pre-op baseline, was 11% [89].

Out of 41 patients, six were no longer receiving DBS at four-years within the Luyten (2016) cohort, three had their DBS switched off, and three had DBS explanted and subsequently underwent capsulotomy. Out of these six patients, 50% had chronic active contacts within the ALIC and 20% had chronic contacts within the BNST.

amSTN

In the closed label phase, there was a median improvement of 25% vs 4% with sham, and response rates of 75% vs 38% with sham [90]. There was a significant order effect, active-sham conditions led to 37% improvement and then 8% deterioration; and sham-active conditions led to 16% improvement, then an additional 14% improvement. At 16 and 48-months follow-up mean improvements were 35% and 51%, respectively [91], the response rate was comparable to closed label phase (75%); however, the criterion increased from 25% to 35% YBOCS reduction between the open and closed label phases. Within this study, two patients withdrew due to lack of efficacy [91].

Other targets

Investigations of BNST DBS encompassed one RCT, in which closed label treatment was associated with 43% improvement (50% response), but sham outcomes were not reported [92]. Investigations of ITP DBS involved one open label trial, in which 12-months of treatment led to 52% improvement and all 5 patients were considered responders [93]. One patient had the DBS electrodes explanted despite 51% improvement. The superolateral MFB was targeted in two patients, with 33% and 50% improvements at 12 months [94]. The thalamus (medial dorsal and ventral anterior nucleus) was targeted in four patients: outcomes for one patient were not reported; one had no symptom change and the device was explanted at 6-months; and the other two did not reach response (11% and 17% improvement) [95].

DBS results for TS

Multiple targets within a cohort

Within a cohort, midline thalamic nuclei DBS achieved mean improvement of 45% (n=4), pv-GPi DBS achieved mean improvement of 32% (n=2), and DBS implanted in both targets achieved mean improvement of 36% (n=2) [96]. There were 37.5% responders, all of whom had been implanted in the thalamus; follow-up periods varied between 6 and 95-months.

A randomised controlled case series targeted the CM-Pf thalamus, GPi, and both targets, each leading to 45%, 78%, and 60% improvement respectively; sham outcomes were not reported [97]. Similarly, in

one patient, DBS of the CM-Pf thalamus, amGPi, and both targets achieved 62%, 59%, and 63% improvement respectively, and 21% deterioration from sham [98].

Four patients were implanted with both GPi and lateral STN DBS and improved by 15-49% at six-months, although one patient withdrew due to limited efficacy [99]. Lastly, one patient implanted with CM-Pf thalamic and NAc DBS led to 84% improvement at 1-year [100].

Thalamus

(Centromedian (CM) thalamus; centromedian-parafascicular complex (CM-Pfc) thalamic nuclei; centromedian nucleus- substantia periventricularis - nucleus-ventro-oralis (CM- SP- VOA); centromedian-parafascicular and ventralis oralis complex (CM-Pf- VOA)

The RCT showed CM-SP-VOA thalamic DBS led to improvements of 40% vs 3% with sham, and response in 66% vs 0% sham [101].

Open label CM-Pf thalamic DBS led to improvement of 44% and response in 60% (3 out of 5) patients at three-months [102,103]. CM thalamic DBS led to improvement of 19% and no responders at 6-months [103]. Another investigation of ventral anterior and ventrolateral thalamic DBS led to improvement of 55% at 6-months, which was maintained at 12-months [104].

A long-term investigation of CM-Pf-VOA thalamic DBS showed improvements of 52% at two-years, increasing to 73% at 5-6 years [105-107]. Two-years of CM thalamic DBS led to 30% improvements and 40% response in a pilot study [108]. The RCT targeting the CM-SP-VOA achieved 49% improvements at one-year, with response in all 6 patients [101]. Two retrospective reports of CM-Pfc thalamic DBS led to 30% improvement and 60% response at one-year [109], and 54% improvement and 63% response at last follow-up (2-91 months, mean 26-months) in another cohort [110].

Case studies showed that seven out of eight patients reached response, with long term improvements (8-months to 35-months) ranging from 49% to 81% [111-115]. Overall, of the 76 patients, three had the DBS switched off, one had additional leads implanted within the GPi, and one had initial leads placed within the ALIC and re-implanted in the thalamus [106,111].

Globus pallidus internus

(anterior globus pallidus internus (a-GPi); anterio-medial globus pallidus internus (am-GPi); posterolateral globus pallidus internus (pl-GPi); posteroventral globus pallidus internus (pv-GPi)).

Randomised controlled investigations of a-GPi DBS (including two patients with comorbid dystonia who instead received pv-GPi DBS) showed mean improvement of 22% vs 8% with sham [116]. Another investigation led to 10% improvement vs deterioration of 4% from sham, and response in 29% compared to 22% in sham, although the response criterion (25% improvement) was relatively liberal [117].

Open label am-GPi DBS led to 47% and 50% improvement at one and three-months respectively, and response in 55% [118]. When follow-up outcomes were reported in the same cohort with an additional six patients, improvements reached 44% at six-months and 54% at last follow-up (8-46-months) with 71% responders [119]. Two cohorts who received 12 months of a-GPi DBS achieved 63% [120] and 40% improvements [117]; the latter cohort reached 48% improvement and 75% response at 30 months [121]. A retrospective report of pl-GPi DBS led to 14% and 55% improvement at 6 and 36-months, respectively, and response in 69% [122].

Across 11 case reports 20 patients were included; with the exception of five patients from two sites [123,124] all achieved response, and improvements were between 47%-95%. Non-responders had implants within the posterior or anterior GPi.

Out of 87 patients, one was explanted at one week, one had DBS switched off following repositioning, two withdrew prior to switch on, three withdrew prior to the closed label phase, and one underwent repositioning at 18 months due to limited efficacy [116,117,124].

Other targets

One patient was implanted with globus pallidus externus (GPe) DBS; at three-months 58% improvement was achieved, which increased to 71% at six-months [125]. The battery depleted at a year, with the patient experiencing a partial relapse (to 38% improvement from baseline).

One patient achieved 25% symptom reduction after 18 months of ALIC DBS [126]. Another patient achieved 41% improvement and remission from 30-months of NAc DBS, but short term outcomes were not reported [127]. amSTN was targeted in one patient, in whom three-months of therapy led to 29% improvement; after three-years, 92% improvement was reached [128].

DBS results for BDD

One BDD patient received VC/VS DBS, and 21% improvement was reached after three-months. Leads were replaced at six-months due to inflammation, and three-months after re-implantation improvement relative to initial baseline was recorded as 35% [129].

References

1. D'Urso, G.; Brunoni, A.R.; Mazzaferro, M.P.; Anastasia, A.; de Bartolomeis, A.; Mantovani, A. Transcranial direct current stimulation for obsessive-compulsive disorder: A randomized, controlled, partial crossover trial. *Depress Anxiety* **2016**, *33*, 1132-1140, doi:10.1002/da.22578.
2. Kumar, S.; Kumar, N.; Verma, R. Safety and efficacy of adjunctive transcranial direct current stimulation in treatment-resistant obsessive-compulsive disorder: An open-label trial. *Indian J Psychiatry* **2019**, *61*, 327-334, doi:10.4103/psychiatry.IndianJPsychiatry_509_18.
3. Silva, R.M.; Brunoni, A.R.; Miguel, E.C.; Shavitt, R.G. Transcranial direct current stimulation for treatment-resistant obsessive-compulsive disorder: report on two cases and proposal for a randomized, sham-controlled trial. *Sao Paulo Med J* **2016**, *134*, 446-450, doi:10.1590/1516-3180.2016.0155010716.
4. Gowda, S.M.; Narayanaswamy, J.C.; Hazari, N.; Bose, A.; Chhabra, H.; Balachander, S.; Bhaskarapillai, B.; Shivakumar, V.; Venkatasubramanian, G.; Reddy, Y.C.J. Efficacy of pre-supplementary motor area transcranial direct current stimulation for treatment resistant obsessive compulsive disorder: A randomized, double blinded, sham controlled trial. *Brain Stimul* **2019**, *12*, 922-929, doi:10.1016/j.brs.2019.02.005.
5. Narayanaswamy, J.C.; Jose, D.; Chhabra, H.; Agarwal, S.M.; Shrinivasa, B.; Hegde, A.; Bose, A.; Kalmady, S.V.; Venkatasubramanian, G.; Reddy, Y.C. Successful Application of Add-on Transcranial Direct Current Stimulation (tDCS) for Treatment of SSRI Resistant OCD. *Brain Stimul* **2015**, *8*, 655-657, doi:10.1016/j.brs.2014.12.003.
6. Hazari, N.; Narayanaswamy, J.C.; Chhabra, H.; Bose, A.; Venkatasubramanian, G.; Reddy, Y.C.J. Response to Transcranial Direct Current Stimulation in a Case of Episodic Obsessive Compulsive Disorder. *The Journal of ECT* **2016**, *32*, 144-146, doi:10.1097/yct.0000000000000309.
7. D'Urso, G.; Brunoni, A.R.; Anastasia, A.; Micillo, M.; de Bartolomeis, A.; Mantovani, A. Polarity-dependent effects of transcranial direct current stimulation in obsessive-compulsive disorder. *Neurocase* **2016**, *22*, 60-64, doi:10.1080/13554794.2015.1045522.
8. Najafi, K.; Fakour, Y.; Zarrabi, H.; Heidarzadeh, A.; Khalkhali, M.; Yeganeh, T.; Farahi, H.; Rostamkhani, M.; Najafi, T.; Shabafroz, S.; et al. Efficacy of Transcranial Direct Current Stimulation in the Treatment:

Resistant Patients who Suffer from Severe Obsessive-compulsive Disorder. *Indian J Psychol Med* **2017**, *39*, 573-578, doi:10.4103/IJPSYM.IJPSYM_388_16.
9. Volpato, C.; Piccione, F.; Cavinato, M.; Duzzi, D.; Schiff, S.; Foscolo, L.; Venneri, A. Modulation of affective symptoms and resting state activity by brain stimulation in a treatment-resistant case of obsessive-compulsive disorder. *Neurocase* **2013**, *19*, 360-370, doi:10.1080/13554794.2012.667131.
10. Palm, U.; Leitner, B.; Kirsch, B.; Behler, N.; Kumpf, U.; Wulf, L.; Padberg, F.; Hasan, A. Prefrontal tDCS and sertraline in obsessive compulsive disorder: a case report and review of the literature. *Neurocase* **2017**, *23*, 173-177, doi:10.1080/13554794.2017.1319492.
11. Dinn, W.M.; Aycicegi-Dinn, A.; Göral, F.; Karamursel, S.; Yildirim, E.A.; Hacioglu-Yildirim, M.; Gansler, D.A.; Doruk, D.; Fregni, F. Treatment-resistant obsessive-compulsive disorder: Insights from an open trial of transcranial direct current stimulation (tDCS) to design a RCT. *Neurology, Psychiatry and Brain Research* **2016**, *22*, 146-154, doi:https://doi.org/10.1016/j.npbr.2016.08.003.
12. Bation, R.; Mondino, M.; Le Camus, F.; Saoud, M.; Brunelin, J. Transcranial direct current stimulation in patients with obsessive compulsive disorder: A randomized controlled trial. *Eur Psychiatry* **2019**, *62*, 38-44, doi:10.1016/j.eurpsy.2019.08.011.
13. Bation, R.; Poulet, E.; Haesebaert, F.; Saoud, M.; Brunelin, J. Transcranial direct current stimulation in treatment-resistant obsessive-compulsive disorder: An open-label pilot study. *Prog Neuropsychopharmacol Biol Psychiatry* **2016**, *65*, 153-157, doi:10.1016/j.pnpbp.2015.10.001.
14. Mondino, M.; Haesebaert, F.; Poulet, E.; Saoud, M.; Brunelin, J. Efficacy of Cathodal Transcranial Direct Current Stimulation Over the Left Orbitofrontal Cortex in a Patient With Treatment-Resistant Obsessive-Compulsive Disorder. *J ECT* **2015**, *31*, 271-272, doi:10.1097/YCT.0000000000000218.
15. Alizadeh Goradel, J.; Pouresmali, A.; Mowlaie, M.; Sadeghi Movahed, F. The Effects of Transcranial Direct Current Stimulation on Obsession-compulsion, Anxiety, and Depression of a Patient Suffering from Obsessive-compulsive Disorder. *Practice in Clinical Psychology* **2016**, *4*, 75-80, doi:10.15412/j.Jpcp.06040201.
16. Carvalho, S.; Goncalves, O.F.; Soares, J.M.; Sampaio, A.; Macedo, F.; Fregni, F.; Leite, J. Sustained Effects of a Neural-based Intervention in a Refractory Case of Tourette Syndrome. *Brain Stimul* **2015**, *8*, 657-659, doi:10.1016/j.brs.2014.12.008.
17. Eapen, V.; Baker, R.; Walter, A.; Raghupathy, V.; Wehrman, J.J.; Sowman, P.F. The Role of Transcranial Direct Current Stimulation (tDCS) in Tourette Syndrome: A Review and Preliminary Findings. *Brain Sci* **2017**, *7*, doi:10.3390/brainsci7120161.
18. Behler, N.; Leitner, B.; Mezger, E.; Weidinger, E.; Musil, R.; Blum, B.; Kirsch, B.; Wulf, L.; Lohrs, L.; Winter, C.; et al. Cathodal tDCS Over Motor Cortex Does Not Improve Tourette Syndrome: Lessons Learned From a Case Series. *Front Behav Neurosci* **2018**, *12*, 194, doi:10.3389/fnbeh.2018.00194.
19. Mrakic-Sposta, S.; Marceglia, S.; Mameli, F.; Dilena, R.; Tadini, L.; Priori, A. Transcranial direct current stimulation in two patients with Tourette syndrome. *Movement Disorders* **2008**, *23*, 2259-2261, doi:10.1002/mds.22292.
20. Haghighi, M.; Shayganfard, M.; Jahangard, L.; Ahmadpanah, M.; Bajoghli, H.; Pirdehghan, A.; Holsboer-Trachsler, E.; Brand, S. Repetitive Transcranial Magnetic Stimulation (rTMS) improves symptoms and reduces clinical illness in patients suffering from OCD--Results from a single-blind, randomized clinical trial with sham cross-over condition. *J Psychiatr Res* **2015**, *68*, 238-244, doi:10.1016/j.jpsychires.2015.06.020.
21. Xiaoyan, M.; Yueqin, H.; Liwei, L.; Yi, J. A randomized double-blinded sham-controlled trial of α electroencephalogram-guided transcranial magnetic stimulation for obsessive-compulsive disorder. *Chinese Medical Journal* **2014**, *127*, 601-606, doi:10.3760/cma.j.issn.0366-6999.20131994.
22. Sachdev, P.S.; McBride, R.; Loo, C.K.; Mitchell, P.B.; Malhi, G.S.; Croker, V.M. Right versus left prefrontal transcranial magnetic stimulation for obsessive-compulsive disorder: a preliminary investigation. *The Journal of clinical psychiatry* **2001**, *62*, 981-984, doi:10.4088/jcp.v62n1211.
23. Prasko, J.; Pasková, B.; Záleský, R.; Novák, T.; Kopecek, M.; Bares, M.; Horácek, J. The effect of repetitive transcranial magnetic stimulation (rTMS) on symptoms in obsessive compulsive disorder. A randomized, double blind, sham controlled study. *Neuro Endocrinol Lett* **2006**, *27*, 327-332.
24. Winkelbeiner, S., M.Sc. ,; Suker, S., M.D. ,; Bachofner, H., M.Sc. ,; Eisenhardt, S., M.D. ,; Steinau, S., M.D. ,; Walther, S., M.D. ,; Federspiel, A., Ph.D. ,; Dierks, T., M.D. ,; Strik, W., M.D. ,; Homan, P., M.D., Ph.D. Targeting Obsessive-Compulsive Symptoms With rTMS and Perfusion Imaging. *American Journal of Psychiatry* **2018**, *175*, 81-83, doi:10.1176/appi.ajp.2017.17060634.

25. Sachdev, P.S.; Loo, C.K.; Mitchell, P.B.; McFarquhar, T.F.; Malhi, G.S. Repetitive transcranial magnetic stimulation for the treatment of obsessive compulsive disorder: a double-blind controlled investigation. *Psychol Med* **2007**, *37*, 1645-1649, doi:10.1017/S0033291707001092.
26. Badawy, A.; El-Sawy, H.; Abdelhay, M. Efficacy of repetitive transcranial magnetic stimulations in the management of obsessive compulsive disorder. *European Neuropsychopharmacology* **2010**, *20*, S541-S542, doi:10.1016/s0924-977x(10)70814-2.
27. Alonso, P., M.D. ,; Pujol, J., M.D., Ph.D. ,; Cardoner, N., M.D. ,; Benlloch, L., M.D. ,; Deus, J., Ph.D. ,; Menchón, J.M., M.D., Ph.D. ,; Capdevila, A., M.D., Ph.D. , and; Vallejo, J., M.D., Ph.D. Right Prefrontal Repetitive Transcranial Magnetic Stimulation in Obsessive-Compulsive Disorder: A Double-Blind, Placebo-Controlled Study. *American Journal of Psychiatry* **2001**, *158*, 1143-1145, doi:10.1176/appi.ajp.158.7.1143.
28. Seo, H.J.; Jung, Y.E.; Lim, H.K.; Um, Y.H.; Lee, C.U.; Chae, J.H. Adjunctive Low-frequency Repetitive Transcranial Magnetic Stimulation over the Right Dorsolateral Prefrontal Cortex in Patients with Treatment-resistant Obsessive-compulsive Disorder: A Randomized Controlled Trial. *Clin Psychopharmacol Neurosci* **2016**, *14*, 153-160, doi:10.9758/cpn.2016.14.2.153.
29. Kang, J.I.; Kim, C.H.; Namkoong, K.; Lee, C.I.; Kim, S.J. A randomized controlled study of sequentially applied repetitive transcranial magnetic stimulation in obsessive-compulsive disorder. *J Clin Psychiatry* **2009**, *70*, 1645-1651, doi:10.4088/JCP.08m04500.
30. Wu, C.C.; Tsai, C.C.; Lu, M.K.; Chen, C.M.; Shen, W.C.; Su, K.P. Theta-Burst Repetitive Transcranial Magnetic Stimulation for Treatment-Resistant Obsessive-Compulsive Disorder with Concomitant Depression. *Journal of Clin Psychiatry* **2010**, *71*, 504-506.
31. Mansur, C.G.; Myczkowki, M.L.; de Barros Cabral, S.; Sartorelli Mdo, C.; Bellini, B.B.; Dias, A.M.; Bernik, M.A.; Marcolin, M.A. Placebo effect after prefrontal magnetic stimulation in the treatment of resistant obsessive-compulsive disorder: a randomized controlled trial. *Int J Neuropsychopharmacol* **2011**, *14*, 1389-1397, doi:10.1017/S1461145711000575.
32. Sarkhel, S.; Sinha, V.K.; Praharaj, S.K. Adjunctive high-frequency right prefrontal repetitive transcranial magnetic stimulation (rTMS) was not effective in obsessive-compulsive disorder but improved secondary depression. *J Anxiety Disord* **2010**, *24*, 535-539, doi:10.1016/j.janxdis.2010.03.011.
33. Elbeh, K.A.M.; Elserogy, Y.M.B.; Khalifa, H.E.; Ahmed, M.A.; Hafez, M.H.; Khedr, E.M. Repetitive transcranial magnetic stimulation in the treatment of obsessive-compulsive disorders: Double blind randomized clinical trial. *Psychiatry Res* **2016**, *238*, 264-269, doi:10.1016/j.psychres.2016.02.031.
34. Mantovani, A.; Simpson, H.B.; Fallon, B.A.; Rossi, S.; Lisanby, S.H. Randomized sham-controlled trial of repetitive transcranial magnetic stimulation in treatment-resistant obsessive-compulsive disorder. *Int J Neuropsychopharmacol* **2010**, *13*, 217-227, doi:10.1017/S1461145709990435.
35. Gomes, P.V., M.D. ,; Brasil-Neto, J.P., M.D., Ph.D. ,; Allam, N., M.D., Ph.D. , and; de Souza, E.R., D.Psy. A Randomized, Double-Blind Trial of Repetitive Transcranial Magnetic Stimulation in Obsessive-Compulsive Disorder With Three-Month Follow-Up. *The Journal of Neuropsychiatry and Clinical Neurosciences* **2012**, *24*, 437-443, doi:10.1176/appi.neuropsych.11100242.
36. Hawken, E.R.; Dilkov, D.; Kaludiev, E.; Simek, S.; Zhang, F.; Milev, R. Transcranial Magnetic Stimulation of the Supplementary Motor Area in the Treatment of Obsessive-Compulsive Disorder: A Multi-Site Study. *Int J Mol Sci* **2016**, *17*, 420, doi:10.3390/ijms17030420.
37. Pelissolo, A.; Harika-Germaneau, G.; Rachid, F.; Gaudeau-Bosma, C.; Tanguy, M.L.; BenAdhira, R.; Bouaziz, N.; Popa, T.; Wassouf, I.; Saba, G.; et al. Repetitive Transcranial Magnetic Stimulation to Supplementary Motor Area in Refractory Obsessive-Compulsive Disorder Treatment: a Sham-Controlled Trial. *Int J Neuropsychopharmacol* **2016**, *19*, doi:10.1093/ijnp/pyw025.
38. Arumugham, S.S.; Vs, S.; Hn, M.; B, V.; Ravi, M.; Sharma, E.; Thirthalli, J.; Reddy, Y.C.J. Augmentation Effect of Low-Frequency Repetitive Transcranial Magnetic Stimulation Over Presupplementary Motor Area in Obsessive-Compulsive Disorder: A Randomized Controlled Trial. *J ECT* **2018**, *34*, 253-257, doi:10.1097/YCT.0000000000000509.
39. Pallanti, S.; Marras, A.; Salerno, L.; Makris, N.; Hollander, E. Better than treated as usual: Transcranial magnetic stimulation augmentation in selective serotonin reuptake inhibitor-refractory obsessive-compulsive disorder, mini-review and pilot open-label trial. *J Psychopharmacol* **2016**, *30*, 568-578, doi:10.1177/0269881116628427.
40. Mantovani, A.; Lisanby, S.H.; Pieraccini, F.; Ulivelli, M.; Castrogiovanni, P.; Rossi, S. Repetitive transcranial magnetic stimulation (rTMS) in the treatment of obsessive-compulsive disorder (OCD) and Tourette's syndrome (TS). *Int J Neuropsychopharmacol* **2006**, *9*, 95-100, doi:10.1017/S1461145705005729.

41. Kumar, N.; Chadda, R.K. Augmentation effect of repetitive transcranial magnetic stimulation over the supplementary motor cortex in treatment refractory patients with obsessive compulsive disorder. *Indian J Psychiatry* **2011**, *53*, 340-342, doi:10.4103/0019-5545.91909.
42. Lee, Y.J.; Koo, B.H.; Seo, W.S.; Kim, H.G.; Kim, J.Y.; Cheon, E.J. Repetitive transcranial magnetic stimulation of the supplementary motor area in treatment-resistant obsessive-compulsive disorder: An open-label pilot study. *J Clin Neurosci* **2017**, *44*, 264-268, doi:10.1016/j.jocn.2017.06.057.
43. Talaei, A.; Morteza-Nia, M.; Jafar-Zadeh, M.; Saghebi, A.; Rezaei Ardani, A. Dramatic Response of Resistant Obsessive Compulsive Disorder to Repeated Transcranial Magnetic Stimulation on Right Supplementary Motor Area. *Iranian Journal of Medical Sciences* **2009**, *34*, 295-298.
44. Mantovani, A.; Westin, G.; Hirsch, J.; Lisanby, S.H. Functional magnetic resonance imaging guided transcranial magnetic stimulation in obsessive-compulsive disorder. *Biol Psychiatry* **2010**, *67*, e39-40, doi:10.1016/j.biopsych.2009.08.009.
45. Kar, S.K.; Dwivedi, S.; Agarwal, V. Relevance of extended protocol and maintenance TMS in obsessive-compulsive disorder: A case report. *Asian J Psychiatr* **2019**, *42*, 32-33, doi:10.1016/j.ajp.2019.03.014.
46. Donse, L.; Sack, A.T.; Fitzgerald, P.B.; Arns, M. Sleep disturbances in obsessive-compulsive disorder: Association with non-response to repetitive transcranial magnetic stimulation (rTMS). *J Anxiety Disord* **2017**, *49*, 31-39, doi:10.1016/j.janxdis.2017.03.006.
47. Harika-Germaneau, G.; Rachid, F.; Chatard, A.; Lafay-Chebassier, C.; Solinas, M.; Thirioux, B.; Millet, B.; Langbour, N.; Jaafari, N. Continuous theta burst stimulation over the supplementary motor area in refractory obsessive-compulsive disorder treatment: A randomized sham-controlled trial. *Brain Stimul* **2019**, *12*, 1565-1571, doi:10.1016/j.brs.2019.07.019.
48. Ruffini, C.; Locatelli, M.; Lucca, A.; Benedetti, F.; Insacco, C.; Smeraldi, E. Augmentation effect of repetitive transcranial magnetic stimulation over the orbitofrontal cortex in drug-resistant obsessive-compulsive disorder patients: a controlled investigation. *Prim Care Companion J Clin Psychiatry* **2009**, *11*, 226-230, doi:10.4088/PCC.08m00663.
49. Nauczyciel, C.; Le Jeune, F.; Naudet, F.; Douabin, S.; Esquevin, A.; Verin, M.; Dondaine, T.; Robert, G.; Drapier, D.; Millet, B. Repetitive transcranial magnetic stimulation over the orbitofrontal cortex for obsessive-compulsive disorder: a double-blind, crossover study. *Transl Psychiatry* **2014**, *4*, e436, doi:10.1038/tp.2014.62.
50. Kumar, S.; Singh, S.; Chadda, R.K.; Verma, R.; Kumar, N. The Effect of Low-Frequency Repetitive Transcranial Magnetic Stimulation at Orbitofrontal Cortex in the Treatment of Patients With Medication-Refractory Obsessive-Compulsive Disorder: A Retrospective Open Study. *J ECT* **2018**, *34*, e16-e19, doi:10.1097/YCT.0000000000000462.
51. Singh, S.; Kumar, S.; Gupta, A.; Verma, R.; Kumar, N. Effectiveness and Predictors of Response to 1-Hz Repetitive Transcranial Magnetic Stimulation in Patients With Obsessive-Compulsive Disorder. *J ECT* **2019**, *35*, 61-66, doi:10.1097/YCT.0000000000000530.
52. Carmi, L.; Alyagon, U.; Barnea-Ygael, N.; Zohar, J.; Dar, R.; Zangen, A. Clinical and electrophysiological outcomes of deep TMS over the medial prefrontal and anterior cingulate cortices in OCD patients. *Brain Stimul* **2018**, *11*, 158-165, doi:10.1016/j.brs.2017.09.004.
53. Carmi, L.; Tendler, A.; Bystritsky, A.; Hollander, E.; Blumberger, D.M.; Daskalakis, J.; Ward, H.; Lapidus, K.; Goodman, W.; Casuto, L.; et al. Efficacy and Safety of Deep Transcranial Magnetic Stimulation for Obsessive-Compulsive Disorder: A Prospective Multicenter Randomized Double-Blind Placebo-Controlled Trial. *Am J Psychiatry* **2019**, *176*, 931-938, doi:10.1176/appi.ajp.2019.18101180.
54. Modirrousta, M.; Shams, E.; Katz, C.; Mansouri, B.; Moussavi, Z.; Sareen, J.; Enns, M. The efficacy of deep repetitive transcranial magnetic stimulation over the medial prefrontal cortex in obsessive compulsive disorder: results from an open-label study. *Depress Anxiety* **2015**, *32*, 445-450, doi:10.1002/da.22363.
55. Dunlop, K.; Woodside, B.; Olmsted, M.; Colton, P.; Giacobbe, P.; Downar, J. Reductions in Cortico-Striatal Hyperconnectivity Accompany Successful Treatment of Obsessive-Compulsive Disorder with Dorsomedial Prefrontal rTMS. *Neuropsychopharmacology* **2016**, *41*, 1395-1403, doi:10.1038/npp.2015.292.
56. Wu, S.W.; Maloney, T.; Gilbert, D.L.; Dixon, S.G.; Horn, P.S.; Huddleston, D.A.; Eaton, K.; Vannest, J. Functional MRI-navigated repetitive transcranial magnetic stimulation over supplementary motor area in chronic tic disorders. *Brain Stimul* **2014**, *7*, 212-218, doi:10.1016/j.brs.2013.10.005.
57. Landeros-Weisenberger, A.; Mantovani, A.; Motlagh, M.G.; de Alvarenga, P.G.; Katsovich, L.; Leckman, J.F.; Lisanby, S.H. Randomized Sham Controlled Double-blind Trial of Repetitive Transcranial Magnetic

Stimulation for Adults With Severe Tourette Syndrome. *Brain Stimul* **2015**, *8*, 574-581, doi:10.1016/j.brs.2014.11.015.

58. Kwon, H.J.; Lim, W.S.; Lim, M.H.; Lee, S.J.; Hyun, J.K.; Chae, J.H.; Paik, K.C. 1-Hz low frequency repetitive transcranial magnetic stimulation in children with Tourette's syndrome. *Neurosci Lett* **2011**, *492*, 1-4, doi:10.1016/j.neulet.2011.01.007.
59. Le, K.; Liu, L.; Sun, M.; Hu, L.; Xiao, N. Transcranial magnetic stimulation at 1 Hertz improves clinical symptoms in children with Tourette syndrome for at least 6 months. *J Clin Neurosci* **2013**, *20*, 257-262, doi:10.1016/j.jocn.2012.01.049.
60. Bloch, Y.; Arad, S.; Levkovitz, Y. Deep TMS add-on treatment for intractable Tourette syndrome: A feasibility study. *World J Biol Psychiatry* **2016**, *17*, 557-561, doi:10.3109/15622975.2014.964767.
61. Mantovani, A.; Leckman, J.F.; Grantz, H.; King, R.A.; Sporn, A.L.; Lisanby, S.H. Repetitive Transcranial Magnetic Stimulation of the Supplementary Motor Area in the treatment of Tourette Syndrome: report of two cases. *Clin Neurophysiol* **2007**, *118*, 2314-2315, doi:10.1016/j.clinph.2007.07.011.
62. Salatino, A.; Momo, E.; Nobili, M.; Berti, A.; Ricci, R. Awareness of symptoms amelioration following low-frequency repetitive transcranial magnetic stimulation in a patient with Tourette syndrome and comorbid obsessive-compulsive disorder. *Brain Stimul* **2014**, *7*, 341-343, doi:10.1016/j.brs.2014.01.002.
63. Orth, M.; Kirby, R.; Richardson, M.P.; Snijders, A.H.; Rothwell, J.C.; Trimble, M.R.; Robertson, M.M.; Munchau, A. Subthreshold rTMS over pre-motor cortex has no effect on tics in patients with Gilles de la Tourette syndrome. *Clin Neurophysiol* **2005**, *116*, 764-768, doi:10.1016/j.clinph.2004.10.003.
64. Chae, J.-H.; Nahas, Z.; Wassermann, E.; Li, X.; Sethuraman, G.; Gilbert, D.; Sallee, F.R.; George, M.S. A Pilot Safety Study of Repetitive Transcranial Magnetic Stimulation (rTMS) in Tourette's Syndrome. *Cognitive and Behavioral Neurology* **2004**, *17*, 109-117, doi:10.1097/01.wnn.0000116253.78804.3a.
65. Aydin, E.P.; Kenar, J.G.; Altunay, I.K.; Kaymak, D.; Ozer, O.A.; Karamustafalioglu, K.O. Repetitive Transcranial Magnetic Stimulation in the Treatment of Skin Picking Disorder: An Exploratory Trial. *J ECT* **2020**, *36*, 60-65, doi:10.1097/YCT.0000000000000616.
66. Diefenbach, G.J.; Tolin, D.F.; Hallion, L.S.; Zertuche, L.; Rabany, L.; Goethe, J.W.; Assaf, M. A case study of clinical and neuroimaging outcomes following repetitive transcranial magnetic stimulation for hoarding disorder. *Am J Psychiatry* **2015**, *172*, 1160-1162, doi:10.1176/appi.ajp.2015.15050602.
67. Tyagi, H.; Apergis-Schoute, A.M.; Akram, H.; Foltynie, T.; Limousin, P.; Drummond, L.M.; Fineberg, N.A.; Matthews, K.; Jahanshahi, M.; Robbins, T.W.; et al. A Randomized Trial Directly Comparing Ventral Capsule and Anteromedial Subthalamic Nucleus Stimulation in Obsessive-Compulsive Disorder: Clinical and Imaging Evidence for Dissociable Effects. *Biol Psychiatry* **2019**, *85*, 726-734, doi:10.1016/j.biopsych.2019.01.017.
68. Islam, L.; Franzini, A.; Messina, G.; Scarone, S.; Gambini, O. Deep brain stimulation of the nucleus accumbens and bed nucleus of stria terminalis for obsessive-compulsive disorder: a case series. *World Neurosurg* **2015**, *83*, 657-663, doi:10.1016/j.wneu.2014.12.024.
69. Farrand, S.; Evans, A.H.; Mangelsdorf, S.; Loi, S.M.; Mocellin, R.; Borham, A.; Bevilacqua, J.; Blair-West, S.; Walterfang, M.A.; Bittar, R.G.; et al. Deep brain stimulation for severe treatment-resistant obsessive-compulsive disorder: An open-label case series. *Australian & New Zealand Journal of Psychiatry* **2018**, *52*, 699-708, doi:10.1177/0004867417731819.
70. Huff, W.; Lenartz, D.; Schormann, M.; Lee, S.H.; Kuhn, J.; Koulousakis, A.; Mai, J.; Daumann, J.; Maarouf, M.; Klosterkotter, J.; et al. Unilateral deep brain stimulation of the nucleus accumbens in patients with treatment-resistant obsessive-compulsive disorder: Outcomes after one year. *Clin Neurol Neurosurg* **2010**, *112*, 137-143, doi:10.1016/j.clineuro.2009.11.006.
71. Barcia, J.A.; Avecillas-Chasin, J.M.; Nombela, C.; Arza, R.; Garcia-Albea, J.; Pineda-Pardo, J.A.; Reneses, B.; Strange, B.A. Personalized striatal targets for deep brain stimulation in obsessive-compulsive disorder. *Brain Stimul* **2019**, *12*, 724-734, doi:10.1016/j.brs.2018.12.226.
72. Mantione, M.; Nieman, D.H.; Figee, M.; Denys, D. Cognitive-behavioural therapy augments the effects of deep brain stimulation in obsessive-compulsive disorder. *Psychol Med* **2014**, *44*, 3515-3522, doi:10.1017/S0033291714000956.
73. Huys, D.; Kohl, S.; Baldermann, J.C.; Timmermann, L.; Sturm, V.; Visser-Vandewalle, V.; Kuhn, J. Open-label trial of anterior limb of internal capsule-nucleus accumbens deep brain stimulation for obsessive-compulsive disorder: insights gained. *J Neurol Neurosurg Psychiatry* **2019**, *90*, 805-812, doi:10.1136/jnnp-2018-318996.
74. Franzini, A.; Messina, G.; Gambini, O.; Muffatti, R.; Scarone, S.; Cordella, R.; Broggi, G. Deep-brain stimulation of the nucleus accumbens in obsessive compulsive disorder: clinical, surgical and

electrophysiological considerations in two consecutive patients. *Neurol Sci* **2010**, *31*, 353-359, doi:10.1007/s10072-009-0214-8.
75. Grant, J.E., J.D., M.D. ,; Odlaug, B.L., B.A. , and; Chamberlain, S.R., M.D., Ph.D. Neurocognitive Response to Deep Brain Stimulation for Obsessive-Compulsive Disorder: A Case Report. *American Journal of Psychiatry* **2011**, *168*, 1338-1339, doi:10.1176/appi.ajp.2011.11071108.
76. Burdick, A.; Foote, K.D.; Goodman, W.; Ward, H.E.; Ricciuti, N.; Murphy, T.; Haq, I.; Okun, M.S. Lack of benefit of accumbens/capsular deep brain stimulation in a patient with both tics and obsessive-compulsive disorder. *Neurocase* **2010**, *16*, 321-330, doi:10.1080/13554790903560422.
77. Nuttin, B.J.; Gabriels, L.A.; Cosyns, P.R.; Meyerson, B.A.; Andreewitch, S.; Sunaert, S.G.; Maes, A.F.; Dupont, P.J.; Gybels, J.M.; Gielen, F.; et al. Long-term electrical capsular stimulation in patients with obsessive-compulsive disorder. *Neurosurgery* **2003**, *52*, 1263-1272; discussion 1272-1264, doi:10.1227/01.neu.0000064565.49299.9a.
78. Abelson, J.L.; Curtis, G.C.; Sagher, O.; Albucher, R.C.; Harrigan, M.; Taylor, S.F.; Martis, B.; Giordani, B. Deep brain stimulation for refractory obsessive-compulsive disorder. *Biol Psychiatry* **2005**, *57*, 510-516, doi:10.1016/j.biopsych.2004.11.042.
79. Goodman, W.K.; Foote, K.D.; Greenberg, B.D.; Ricciuti, N.; Bauer, R.; Ward, H.; Shapira, N.A.; Wu, S.S.; Hill, C.L.; Rasmussen, S.A.; et al. Deep brain stimulation for intractable obsessive compulsive disorder: pilot study using a blinded, staggered-onset design. *Biol Psychiatry* **2010**, *67*, 535-542, doi:10.1016/j.biopsych.2009.11.028.
80. Fayad, S.M.; Guzick, A.G.; Reid, A.M.; Mason, D.M.; Bertone, A.; Foote, K.D.; Okun, M.S.; Goodman, W.K.; Ward, H.E. Six-Nine Year Follow-Up of Deep Brain Stimulation for Obsessive-Compulsive Disorder. *PLOS ONE* **2016**, *11*, e0167875, doi:10.1371/journal.pone.0167875.
81. Liebrand, L.C.; Caan, M.W.A.; Schuurman, P.R.; van den Munckhof, P.; Figee, M.; Denys, D.; van Wingen, G.A. Individual white matter bundle trajectories are associated with deep brain stimulation response in obsessive-compulsive disorder. *Brain Stimul* **2019**, *12*, 353-360, doi:10.1016/j.brs.2018.11.014.
82. Choudhury, T.K.; Davidson, J.E.; Viswanathan, A.; Strutt, A.M. Deep brain stimulation of the anterior limb of the internal capsule for treatment of therapy-refractory obsessive compulsive disorder (OCD): a case study highlighting neurocognitive and psychiatric changes. *Neurocase* **2017**, *23*, 138-145, doi:10.1080/13554794.2017.1319958.
83. Gupta, A.; Khanna, S.; Jain, R. Deep brain stimulation of ventral internal capsule for refractory obsessive-compulsive disorder. *Indian J Psychiatry* **2019**, *61*, 532-536, doi:10.4103/psychiatry.IndianJPsychiatry_222_16.
84. Luyten, L.; Hendrickx, S.; Raymaekers, S.; Gabriels, L.; Nuttin, B. Electrical stimulation in the bed nucleus of the stria terminalis alleviates severe obsessive-compulsive disorder. *Mol Psychiatry* **2016**, *21*, 1272-1280, doi:10.1038/mp.2015.124.
85. Greenberg, B.D.; Malone, D.A.; Friehs, G.M.; Rezai, A.R.; Kubu, C.S.; Malloy, P.F.; Salloway, S.P.; Okun, M.S.; Goodman, W.K.; Rasmussen, S.A. Three-year outcomes in deep brain stimulation for highly resistant obsessive-compulsive disorder. *Neuropsychopharmacology* **2006**, *31*, 2384-2393, doi:10.1038/sj.npp.1301165.
86. Greenberg, B.D.; Gabriels, L.A.; Malone, D.A., Jr.; Rezai, A.R.; Friehs, G.M.; Okun, M.S.; Shapira, N.A.; Foote, K.D.; Cosyns, P.R.; Kubu, C.S.; et al. Deep brain stimulation of the ventral internal capsule/ventral striatum for obsessive-compulsive disorder: worldwide experience. *Mol Psychiatry* **2010**, *15*, 64-79, doi:10.1038/mp.2008.55.
87. Tsai, H.C.; Chang, C.H.; Pan, J.I.; Hsieh, H.J.; Tsai, S.T.; Hung, H.Y.; Chen, S.Y. Acute stimulation effect of the ventral capsule/ventral striatum in patients with refractory obsessive-compulsive disorder - a double-blinded trial. *Neuropsychiatr Dis Treat* **2014**, *10*, 63-69, doi:10.2147/NDT.S54964.
88. Roh, D.; Chang, W.S.; Chang, J.W.; Kim, C.H. Long-term follow-up of deep brain stimulation for refractory obsessive-compulsive disorder. *Psychiatry Res* **2012**, *200*, 1067-1070, doi:10.1016/j.psychres.2012.06.018.
89. Chang, C.H.; Chen, S.Y.; Tsai, S.T.; Tsai, H.C. Compulsive skin-picking behavior after deep brain stimulation in a patient with refractory obsessive-compulsive disorder: A case report. *Medicine (Baltimore)* **2017**, *96*, e8012, doi:10.1097/MD.0000000000008012.
90. Mallet, L.; Polosan, M.; Jaafari, N.; Baup, N.; Welter, M.-L.; Fontaine, D.; Montcel, S.T.d.; Yelnik, J.; Chéreau, I.; Arbus, C.; et al. Subthalamic Nucleus Stimulation in Severe Obsessive–Compulsive Disorder. *New England Journal of Medicine* **2008**, *359*, 2121-2134, doi:10.1056/NEJMoa0708514.

91. Mallet, L.; Du Montcel, S.T.; Clair, A.H.; Arbus, C.; Bardinet, E.; Baup, N.; Chabardes, S.; Chereau, I.; Czernecki, V.; Fontaine, D.; et al. Long-term effects of subthalamic stimulation in Obsessive-Compulsive Disorder: Follow-up of a randomized controlled trial. *Brain Stimul* **2019**, *12*, 1080-1082, doi:10.1016/j.brs.2019.04.004.
92. Suetens, K.; Nuttin, B.; Gabriels, L.; Van Laere, K. Differences in metabolic network modulation between capsulotomy and deep-brain stimulation for refractory obsessive-compulsive disorder. *J Nucl Med* **2014**, *55*, 951-959, doi:10.2967/jnumed.113.126409.
93. Lee, D.J.; Dallapiazza, R.F.; De Vloo, P.; Elias, G.J.B.; Fomenko, A.; Boutet, A.; Giacobbe, P.; Lozano, A.M. Inferior thalamic peduncle deep brain stimulation for treatment-refractory obsessive-compulsive disorder: A phase 1 pilot trial. *Brain Stimulation* **2019**, *12*, 344-352, doi:https://doi.org/10.1016/j.brs.2018.11.012.
94. Coenen, V.A.; Schlaepfer, T.E.; Sajonz, B.; Dobrossy, M.; Kaller, C.P.; Urbach, H.; Reisert, M. Tractographic description of major subcortical projection pathways passing the anterior limb of the internal capsule. Corticopetal organization of networks relevant for psychiatric disorders. *Neuroimage Clin* **2020**, *25*, 102165, doi:10.1016/j.nicl.2020.102165.
95. Maarouf, M.; Neudorfer, C.; El Majdoub, F.; Lenartz, D.; Kuhn, J.; Sturm, V. Deep Brain Stimulation of Medial Dorsal and Ventral Anterior Nucleus of the Thalamus in OCD: A Retrospective Case Series. *PLoS One* **2016**, *11*, e0160750, doi:10.1371/journal.pone.0160750.
96. Motlagh, M.G.; Smith, M.E.; Landeros-Weisenberger, A.; Kobets, A.J.; King, R.A.; Miravite, J.; de Lotbinière, A.C.; Alterman, R.L.; Mogilner, A.Y.; Pourfar, M.H.; et al. Lessons Learned from Open-label Deep Brain Stimulation for Tourette Syndrome: Eight Cases over 7 Years. *Tremor Other Hyperkinet Mov (N Y)* **2013**, *3*, doi:10.7916/d8m32tgm.
97. Welter, M.-L.; Mallet, L.; Houeto, J.-L.; Karachi, C.; Czernecki, V.; Cornu, P.; Navarro, S.; Pidoux, B.; Dormont, D.; Bardinet, E.; et al. Internal Pallidal and Thalamic Stimulation in Patients With Tourette Syndrome. *Archives of Neurology* **2008**, *65*, 952-957, doi:10.1001/archneur.65.7.952.
98. Houeto, J.L.; Karachi, C.; Mallet, L.; Pillon, B.; Yelnik, J.; Mesnage, V.; Welter, M.L.; Navarro, S.; Pelissolo, A.; Damier, P.; et al. Tourette's syndrome and deep brain stimulation. *J Neurol Neurosurg Psychiatry* **2005**, *76*, 992-995, doi:10.1136/jnnp.2004.043273.
99. Zhu, G.Y.; Geng, X.Y.; Zhang, R.L.; Chen, Y.C.; Liu, Y.Y.; Wang, S.Y.; Zhang, J.G. Deep brain stimulation modulates pallidal and subthalamic neural oscillations in Tourette's syndrome. *Brain Behav* **2019**, *9*, e01450, doi:10.1002/brb3.1450.
100. Kakusa, B.; Saluja, S.; Tate, W.J.; Espil, F.M.; Halpern, C.H.; Williams, N.R. Robust clinical benefit of multi-target deep brain stimulation for treatment of Gilles de la Tourette syndrome and its comorbidities. *Brain Stimul* **2019**, *12*, 816-818, doi:10.1016/j.brs.2019.02.026.
101. Ackermans, L.; Duits, A.; van der Linden, C.; Tijssen, M.; Schruers, K.; Temel, Y.; Kleijer, M.; Nederveen, P.; Bruggeman, R.; Tromp, S.; et al. Double-blind clinical trial of thalamic stimulation in patients with Tourette syndrome. *Brain* **2011**, *134*, 832-844, doi:10.1093/brain/awq380.
102. Maciunas, R.J.; Maddux, B.N.; Riley, D.E.; Whitney, C.M.; Schoenberg, M.R.; Ogrocki, P.J.; Albert, J.M.; Gould, D.J. Prospective randomized double-blind trial of bilateral thalamic deep brain stimulation in adults with Tourette syndrome. *J Neurosurg* **2007**, *107*, 1004-1014, doi:10.3171/JNS-07/11/1004.
103. Okun, M.S.; Foote, K.D.; Wu, S.S.; Ward, H.E.; Bowers, D.; Rodriguez, R.L.; Malaty, I.A.; Goodman, W.K.; Gilbert, D.M.; Walker, H.C.; et al. A trial of scheduled deep brain stimulation for Tourette syndrome: moving away from continuous deep brain stimulation paradigms. *JAMA Neurol* **2013**, *70*, 85-94, doi:10.1001/jamaneurol.2013.580.
104. Huys, D.; Bartsch, C.; Koester, P.; Lenartz, D.; Maarouf, M.; Daumann, J.; Mai, J.K.; Klosterkotter, J.; Hunsche, S.; Visser-Vandewalle, V.; et al. Motor Improvement and Emotional Stabilization in Patients With Tourette Syndrome After Deep Brain Stimulation of the Ventral Anterior and Ventrolateral Motor Part of the Thalamus. *Biol Psychiatry* **2016**, *79*, 392-401, doi:10.1016/j.biopsych.2014.05.014.
105. Servello, D.; Porta, M.; Sassi, M.; Brambilla, A.; Robertson, M.M. Deep brain stimulation in 18 patients with severe Gilles de la Tourette syndrome refractory to treatment: the surgery and stimulation. *J Neurol Neurosurg Psychiatry* **2008**, *79*, 136-142, doi:10.1136/jnnp.2006.104067.
106. Porta, M.; Brambilla, A.; Cavanna, A.E.; Servello, D.; Sassi, M.; Rickards, H.; Robertson, M.M. Thalamic deep brain stimulation for treatment-refractory Tourette syndrome. *Two-year outcome* **2009**, *73*, 1375-1380, doi:10.1212/WNL.0b013e3181bd809b.
107. Porta, M.; Servello, D.; Zanaboni, C.; Anasetti, F.; Menghetti, C.; Sassi, M.; Robertson, M.M. Deep brain stimulation for treatment of refractory Tourette syndrome: long-term follow-up. *Acta Neurochir (Wien)* **2012**, *154*, 2029-2041, doi:10.1007/s00701-012-1497-8.

108. Rossi, P.J.; Opri, E.; Shute, J.B.; Molina, R.; Bowers, D.; Ward, H.; Foote, K.D.; Gunduz, A.; Okun, M.S. Scheduled, intermittent stimulation of the thalamus reduces tics in Tourette syndrome. *Parkinsonism Relat Disord* **2016**, *29*, 35-41, doi:10.1016/j.parkreldis.2016.05.033.
109. Brito, M.; Teixeira, M.J.; Mendes, M.M.; Franca, C.; Iglesio, R.; Barbosa, E.R.; Cury, R.G. Exploring the clinical outcomes after deep brain stimulation in Tourette syndrome. *J Neurol Sci* **2019**, *402*, 48-51, doi:10.1016/j.jns.2019.05.011.
110. Testini, P.; Zhao, C.Z.; Stead, M.; Duffy, P.S.; Klassen, B.T.; Lee, K.H. Centromedian-Parafascicular Complex Deep Brain Stimulation for Tourette Syndrome: A Retrospective Study. *Mayo Clin Proc* **2016**, *91*, 218-225, doi:10.1016/j.mayocp.2015.11.016.
111. Shields, D.C.; Cheng, M.L.; Flaherty, A.W.; Gale, J.T.; Eskandar, E.N. Microelectrode-guided deep brain stimulation for Tourette syndrome: within-subject comparison of different stimulation sites. *Stereotact Funct Neurosurg* **2008**, *86*, 87-91, doi:10.1159/000112429.
112. Pullen, S.J.; Wall, C.A.; Lee, K.H.; Stead, S.M.; Klassen, B.T.; Brown, T.M. Neuropsychiatric Outcome of an Adolescent Who Received Deep Brain Stimulation for Tourette's Syndrome. *Case Rep Neurol Med* **2011**, *2011*, 209467, doi:10.1155/2011/209467.
113. Rzesnitzek, L.; Wachter, T.; Kruger, R.; Gharabaghi, A.; Plewnia, C. Suppression of extrapyramidal side effects of doxepin by thalamic deep brain stimulation for Tourette syndrome. *Neurology* **2011**, *77*, 1708-1709, doi:10.1212/WNL.0b013e318236485f.
114. Savica, R.; Stead, M.; Mack, K.J.; Lee, K.H.; Klassen, B.T. Deep brain stimulation in tourette syndrome: a description of 3 patients with excellent outcome. *Mayo Clin Proc* **2012**, *87*, 59-62, doi:10.1016/j.mayocp.2011.08.005.
115. Kano, Y.; Matsuda, N.; Nonaka, M.; Fujio, M.; Kono, T.; Kaido, T. Sensory phenomena and obsessive-compulsive symptoms in Tourette syndrome following deep brain stimulation: Two case reports. *J Clin Neurosci* **2018**, *56*, 199-201, doi:10.1016/j.jocn.2018.06.046.
116. Kefalopoulou, Z.; Zrinzo, L.; Jahanshahi, M.; Candelario, J.; Milabo, C.; Beigi, M.; Akram, H.; Hyam, J.; Clayton, J.; Kass-Iliyya, L.; et al. Bilateral globus pallidus stimulation for severe Tourette's syndrome: a double-blind, randomised crossover trial. *The Lancet Neurology* **2015**, *14*, 595-605, doi:10.1016/s1474-4422(15)00008-3.
117. Welter, M.-L.; Houeto, J.-L.; Thobois, S.; Bataille, B.; Guenot, M.; Worbe, Y.; Hartmann, A.; Czernecki, V.; Bardinet, E.; Yelnik, J.; et al. Anterior pallidal deep brain stimulation for Tourette's syndrome: a randomised, double-blind, controlled trial. *The Lancet Neurology* **2017**, *16*, 610-619, doi:10.1016/s1474-4422(17)30160-6.
118. Cannon, E., M.B.B.S. ,; Silburn, P., M.B.B.S., Ph.D. ,; Coyne, T., M.B.B.S., F.R.A.C.S. ,; O'Maley, K., R.N., M.N. ,; Crawford, J.D., Ph.D. , and; Sachdev, P.S., M.D., Ph.D. Deep Brain Stimulation of Anteromedial Globus Pallidus Interna for Severe Tourette's Syndrome. *American Journal of Psychiatry* **2012**, *169*, 860-866, doi:10.1176/appi.ajp.2012.11101583.
119. Sachdev, P.S.; Mohan, A.; Cannon, E.; Crawford, J.D.; Silberstein, P.; Cook, R.; Coyne, T.; Silburn, P.A. Deep brain stimulation of the antero-medial globus pallidus interna for Tourette syndrome. *PLoS One* **2014**, *9*, e104926, doi:10.1371/journal.pone.0104926.
120. Azimi, A.; Parvaresh, M.; Shahidi, G.; Habibi, A.; Rohani, S.; Safdarian, M.; Fattahi, A.; Taheri, M.; Rohani, M. Anteromedial GPi deep brain stimulation in Tourette syndrome: The first case series from Iran. *Clin Neurol Neurosurg* **2018**, *172*, 116-119, doi:10.1016/j.clineuro.2018.06.045.
121. Welter, M.L.; Houeto, J.L.; Worbe, Y.; Diallo, M.H.; Hartmann, A.; Tezenas du Montcel, S.; Ansquer, S.; Thobois, S.; Fontaine, D.; Rouaud, T.; et al. Long-term effects of anterior pallidal deep brain stimulation for tourette's syndrome. *Mov Disord* **2019**, *34*, 586-588, doi:10.1002/mds.27645.
122. Zhang, J.G.; Ge, Y.; Stead, M.; Zhang, K.; Yan, S.S.; Hu, W.; Meng, F.G. Long-term outcome of globus pallidus internus deep brain stimulation in patients with Tourette syndrome. *Mayo Clin Proc* **2014**, *89*, 1506-1514, doi:10.1016/j.mayocp.2014.05.019.
123. Dehning, S.; Feddersen, B.; Mehrkens, J.H.; Muller, N. Long-term results of electroconvulsive therapy in severe Gilles de la Tourette syndrome. *J ECT* **2011**, *27*, 145-147, doi:10.1097/YCT.0b013e3181eb30b3.
124. Martinez-Fernandez, R.; Zrinzo, L.; Aviles-Olmos, I.; Hariz, M.; Martinez-Torres, I.; Joyce, E.; Jahanshahi, M.; Limousin, P.; Foltynie, T. Deep brain stimulation for Gilles de la Tourette syndrome: a case series targeting subregions of the globus pallidus internus. *Mov Disord* **2011**, *26*, 1922-1930, doi:10.1002/mds.23734.
125. Piedimonte, F.; Andreani, J.C.; Piedimonte, L.; Graff, P.; Bacaro, V.; Micheli, F.; Vilela Filho, O. Behavioral and motor improvement after deep brain stimulation of the globus pallidus externus in a

case of Tourette's syndrome. *Neuromodulation* **2013**, *16*, 55-58; discussion 58, doi:10.1111/j.1525-1403.2012.00526.x.
126. Flaherty, A.W.; Williams, Z.M.; Amirnovin, R.; Kasper, E.; Rauch, S.L.; Cosgrove, G.R.; Eskandar, E.N. Deep brain stimulation of the anterior internal capsule for the treatment of Tourette syndrome: technical case report. *Neurosurgery* **2005**, *57*, E403; discussion E403, doi:10.1227/01.neu.0000176854.24694.95.
127. Kuhn, J.; Lenartz, D.; Mai, J.K.; Huff, W.; Lee, S.H.; Koulousakis, A.; Klosterkoetter, J.; Sturm, V. Deep brain stimulation of the nucleus accumbens and the internal capsule in therapeutically refractory Tourette-syndrome. *J Neurol* **2007**, *254*, 963-965, doi:10.1007/s00415-006-0404-8.
128. Wojtecki, L.; Elben, S.; Rubenach, J.; Hartmann, C.; Vesper, J.; Schnitzler, A. Subthalamic Deep Brain Stimulation in Obsessive-Compulsive Disorder: First German Experience and Future Outlook. *World Neurosurg* **2016**, *95*, 607-608, doi:10.1016/j.wneu.2016.07.033.
129. Baldermann, J.C.; Melzer, C.; Zapf, A.; Kohl, S.; Timmermann, L.; Tittgemeyer, M.; Huys, D.; Visser-Vandewalle, V.; Kuhn, A.A.; Horn, A.; et al. Connectivity Profile Predictive of Effective Deep Brain Stimulation in Obsessive-Compulsive Disorder. *Biol Psychiatry* **2019**, *85*, 735-743, doi:10.1016/j.biopsych.2018.12.019.
