# Supplementary figures and images for: Therapeutic Neurostimulation in Obsessive-Compulsive and Related Disorders: A Systematic Review
Source: Brain Sci. 2021 Jul 19;11(7):948. doi: 10.3390/brainsci11070948 (PMC8307974; doi:10.3390/brainsci11070948)

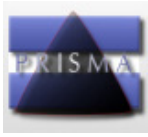

## Neurostimulation PRISMA Flow Diagram

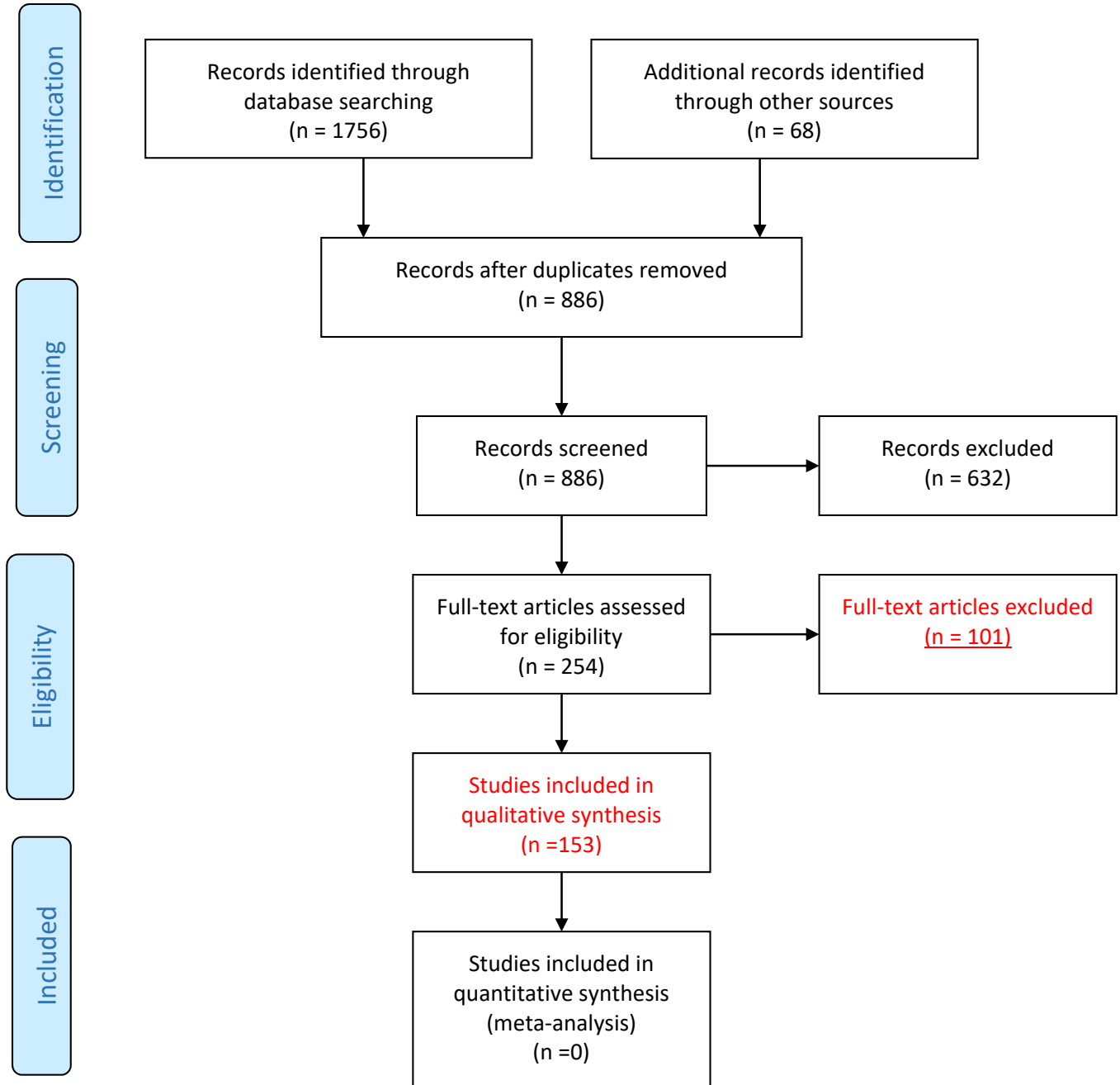

Supplement: Supplementary file 1 [file brainsci-11-00948-s001.zip › S1 PRISMA diagram.pdf]
